# Religious development from adolescence to early adulthood among Muslim and Christian youth in Germany: A person‐oriented approach

**DOI:** 10.1111/cdev.14151

**Published:** 2024-08-27

**Authors:** Olivia Spiegler, Jan O. Jonsson, Chloe Bracegirdle

**Affiliations:** ^1^ Nuffield College University of Oxford Oxford UK; ^2^ Swedish Institute for Social Research (SOFI) Stockholm University Stockholm Sweden; ^3^ Institute for Future Studies Stockholm Sweden; ^4^ Department of Sociology University of Oxford Oxford UK

## Abstract

Religious decline, often observed among North American Christian youth, may not apply universally. We examined this and whether religiosity is associated with well‐being, risk behavior, cultural values, and acculturation among 4080 Muslim and Christian adolescents aged 15–22 in Germany. Utilizing seven waves from the CILS4EU project and a person‐oriented analytical approach, we identified different religious trajectories for Muslim (58% high, 31% low, 11% increasing), immigrant‐origin Christian (68% low, 32% medium), and non‐immigrant Christian (74% low, 17% decreasing, 9% medium) youth. High and medium trajectories were associated with greater well‐being, lower risk behavior, more conservative attitudes, and less sociocultural integration. To fully understand religious development, we must consider diverse national contexts and groups, employing long‐term perspectives and person‐centered analyses.

Religious development is an important dimension of human development (King & Boyatzis, 2015), and religiosity can benefit youths' social and psychological adjustment by providing positive peer networks, moral guidelines, and a sense of purpose (Hardy et al., 2019). Adolescence and early adulthood are, however, developmental stages characterized by declines in religiosity, especially in religious participation (King & Boyatzis, 2015; McNamara Barry et al., 2010; Schnitker et al., 2021). Yet empirical research documenting these declines is largely based on Christian samples in North America. It therefore remains unclear whether the widely observed declines in religiosity during adolescence and early adulthood are a universal or culturally specific trend (King & Boyatzis, 2015; McNamara Barry et al., 2010; Schnitker et al., 2021).

Guided by a framework that contextualizes religious development and stresses the roles of acculturative contexts and intercultural relations (Phalet et al., 2018), we studied the religious development of immigrant‐origin Muslim, immigrant‐origin Christian, and non‐immigrant Christian youth in Germany. Germany is a traditionally Christian, highly secularized Western European country, characterized by a comparatively large and increasing population of Muslims and negative attitudes toward this group (Bell et al., 2021). While we study both Christian and Muslim youth, our focus is on Muslim‐identifying individuals, who, according to one population forecast, will make up 10% of Europe's population by 2050 (PEW, 2017). But demographic trends are not the only reason to focus on Muslim youth. It is far more interesting that the religious development of Muslim youth in Western societies diverges from the religious development of other religious groups. Specifically, studies suggest that religiosity is and continues to be important for European Muslim individuals throughout adolescence (Simsek et al., 2019). This is noteworthy as it contradicts prior findings from Christian samples in North America and assimilation theory's assumption that immigrants gradually adopt to the dominant culture (Alba & Nee, 1997).

We examined within‐person changes in religiosity from age 15 to 22. From a developmental science perspective, it is important to study religious change beyond adolescence, as early adulthood is a distinct developmental period characterized by greater independence and new life tasks that affect religious change (Etengoff & Daiute, 2013; McNamara Barry et al., 2010). We further employed a person‐oriented analytical approach that identifies classes (or subgroups) of adolescents with distinct developmental trajectories because religiosity is a developmental domain that does not follow a uniform trend (e.g., Goodman & Dyer, 2020; Lee & Neblett, 2019; Wright et al., 2018). Finally, we examined whether and how religious trajectories are associated with demographic characteristics, parent religiosity, and long‐term changes in psychological well‐being, risky behavior, cultural values, and sociocultural integration. In so doing, the present research contributes to ongoing debates concerning whether religiosity is a developmental resource or source of distress for acculturating Muslim youth (Goforth et al., 2014; Phalet et al., 2018), a protective resource for religious youth in secularized contexts (Hodapp & Zwingmann, 2019), and whether and how religiosity matters for social and cultural integration (Fleischmann, 2022).

## Religious development during adolescence and early adulthood

Religiosity is defined as the “adherence to beliefs, doctrines, ethics, rituals, texts, traditions, and practices related to a higher power and associated with an organized group” (cf. Lee & Neblett, 2019). It is conceptualized as a multidimensional phenomenon including religious affiliation (membership in a religious group), participation (e.g., service attendance), and identity (e.g., sense of belonging, importance; e.g., Davis & Kiang, 2016).

Adolescence and early adulthood are sensitive periods for religious development marked by increasingly complex experiences and understandings of religious practices and beliefs. During adolescence, developing cognitive skills such as abstract thought, hypothetical reasoning, and meta‐cognition are likely to facilitate religious identity exploration. Moreover, gains in autonomy paired with decreases in parental control affect youths' religious choices and participation in religious activities (King & Boyatzis, 2015; Schnitker et al., 2021). The transition to early adulthood is marked by further significant changes in life circumstances (e.g., moving away from home, developing long‐term romantic relationships) and new responsibilities (e.g., work, college), which are likely to contribute to more individualized religious beliefs (Etengoff & Daiute, 2013) and lower levels of religious involvement (McNamara Barry et al., 2010).

Empirical studies investigating within‐person changes mostly point toward declines in religiosity during adolescence and beyond. Lopez et al. (2011), for example, found declines in religious participation (but not identity) from age 16 to 18 among ethnically (Asian, Latino, White) and religiously (Buddhist, Protestant, Catholic, Jewish, other, non‐affiliated) diverse adolescents in the United States. In the same sample, Chan et al. (2015) found declines in both religious participation and identity from age 18 to 22. Declines in religiosity during early adulthood were also observed among ethnically and religiously diverse U.S. college students (Stoppa & Lefkowitz, 2010; declines in participation, not importance), White Christians from the Minnesota Twin Family Study (Koenig et al., 2008; composite score of religiosity), and Canadians (Hardy et al., 2011; declines in participation, importance, and affiliation not assessed). Dyer et al. (2022) found declines in religiosity from age 12 to 20 among religiously diverse White adolescents in the United States. Importantly, Dyer and colleagues found that changes in religiosity do not necessarily follow a linear trend. Finally, Davis and Kiang (2016) found stable religious identities and *increases* in religious participation from age 14 to 18 among religiously diverse Asian adolescents in the South‐Eastern United States where religious organizations have more social prominence and may therefore retain adolescents in religious institutions.

Taken together, these studies suggest that religiosity, and especially religious participation but less so identity, declines during adolescence and early adulthood. However, many prior studies focused on ethnic/racial groups rather than religious groups. This seems problematic because of the religious diversity within ethnic/racial groups and because religious groups differ in how much commitment and participation they demand from followers (Smith et al., 2002). In addition, many studies seem to have included adolescents who are not affiliated with any religious group. This increases the chance to find overall low levels in religiosity among youth, and thus does not identify religious development among those affiliated. Finally, it appears necessary to examine if changes in religiosity follow a non‐linear trend.

## Heterogeneity in religious development

The aforementioned studies indicate that religiosity declines during adolescence and the transition to early adulthood. All findings were, however, based on variable‐centered analytical approaches (e.g., Latent Growth Curve Modeling, LGCM), which are less well‐suited to uncover groups of adolescents with distinct patterns of religious development (Muthén & Muthén, 2000). To address developmental heterogeneity, it is widely recommended to use person‐oriented instead of variable‐oriented analytical methods. According to Sterba & Bauer (2010), variable‐oriented methods (e.g., LGCM) estimate only one pattern of change for all individuals in a population. It is assumed that all individuals follow more or less the same trajectory with only *quantitative* variation around this population‐level trajectory, but not qualitative variation (aside from a priori groups defined by observed variables, e.g., trajectories of girls and boys). Person‐oriented methods (e.g., Growth Mixture Models, GMM), in contrast, extract latent classes with *qualitatively* different functional forms (e.g., early‐ vs late‐onset decline). In GMM specifically, individuals can further vary quantitatively around their class‐specific trajectories. These characteristics make GMM stand out as the method least prone to ecological fallacy, thus ensuring the validity of conclusions drawn from population‐level data without misrepresenting individual behaviors. Moreover, person‐oriented theory concerns not only identifying potential subgroups of individuals with distinct trajectories in a population, but also identifying the causes and consequences of these trajectories (Nagin, 1999), such as religious upbringing or psychological well‐being.

Several studies have applied person‐oriented methods to study religious development. Petts (2009) identified six groups of ethnically (White, African, Latino) and religiously (Protestant, Catholic, Mormon, Other, non‐affiliated) diverse U.S. adolescents. These groups were characterized by either gradual, early onset, or late onset declines in religious participation from age 10 to 25 or stability at low, moderate, or high levels. Crucially, Petts further showed that family characteristics predicted group membership. Youth in religious family contexts were, for example, more likely to be in the late onset than the early onset decline group. Lee and Neblett (2019) examined changes in religiosity from age 12 to 18 among African American adolescents (Protestant, non‐affiliated, Catholic, other). By using a person‐oriented analytical approach, the authors identified two distinct classes of adolescents characterized by declines in religiosity at either high or low levels. Subsequent analyses showed that adolescents whose religiosity declined at high levels reported lower levels of depressive symptoms in response to stressful life events than adolescents whose religiosity declined at low levels. Goodman and Dyer (2020) identified four classes of religiously diverse White American adolescents with either high stable, high declining, low declining, or low stable religious identities from age 13 to 19 and showed that religious transmission and parent‐adolescent relationships varied across these classes. Finally, Wright et al. (2018) identified three classes of African American adolescents (religious affiliation not assessed) characterized by either high stable, high declining, or low stable religiosity. Being in the high stable religiosity class was related to greater goal‐directedness, life satisfaction, emotion management, and coping strategies than being in the high declining or low stable religiosity classes.

Taken together, these studies show that there is no uniform trend in religious development. Instead, there are classes of adolescents following distinct developmental trajectories including—at the very least—religious decline and stability at different levels. Prior studies further show that these developmental trajectories have unique implications for youth's adjustment and well‐being with higher religiosity being a protective resource.

## Religious development among Muslim‐identifying youth in Western Europe

A major limitation of previous longitudinal studies is that they were largely based on Christian‐identifying youth in North America. Accordingly, critiques continue to highlight the need for more research that investigates religious development among other religious groups and in different national contexts (King & Boyatzis, 2015; McNamara Barry et al., 2010; Schnitker et al., 2021). Contexts shape how youth relate to, understand, and experience their religion (Schnitker et al., 2021). In the context of Indonesia, for example, Muslim adolescents show an increase (rather than a decrease) in religious participation from age 15 to 17 (French et al., 2014). This differs from most North American findings and suggests that declines in religiosity are less likely among religious majority youth in highly religious contexts. Any investigation of religious development must therefore consider the sociocultural context in which youth grow up.

Muslim youth in Western European countries, including Germany, find themselves in highly secularized, traditionally Christian majority contexts in which, despite relatively high levels of nominal affiliation, religiosity is low and mistrust against Muslim people is high (Bell et al., 2021). Most Muslim individuals in Germany are the descendants of young Turkish men who moved to Germany in the 1960s and 1970s as guest workers to address Germany's labor shortage after World War II. While the recruitment of guest workers stopped in 1973, Germany's Turkish population continued to grow because of family reunification, marriage migration, and higher birthrates. For a long time, Germany omitted to integrate guest workers and their families, which contributed to persistent social inequities. Over the past decades, attitudes toward Muslim individuals have become more positive in Germany (especially among younger people), but anti‐Muslim attitudes are still much more prevalent than anti‐immigrant attitudes (Bell et al., 2021).

To maintain a positive social identity in unreceptive contexts, Muslim youth may engage more in their religious identity (Connor, 2010; Peek, 2005). Theoretical approaches support this line of reasoning. Frameworks in developmental psychology, for example, suggest that discrimination experiences stimulate youth's identity exploration and that ethnic/racial identity buffers against negative intergroup experiences (e.g., Umaña‐Taylor et al., 2014). Social identity theory (Tajfel & Turner, 1979) and the rejection‐identification model (Branscombe et al., 1999) also posit that members of devalued groups might identify more strongly with the devalued group to preserve a positive self in light of discrimination, exclusion, and marginalization. Uncertainty identity theory further argues that religious identification is an effective way to reduce general feelings of uncertainty (Hogg et al., 2010). Finally, segmented assimilation theory underscores that immigrants who hold on to aspects of their heritage culture may have greater chances of socioeconomic achievements (Portes & Rumbaut, 2001). Taken together, these theoretical streams suggest that religiosity extends beyond personal beliefs among Muslim individuals in Europe; it is also a marker of differentiation and identity and may therefore remain high (Foner & Alba, 2008).

Comparative cross‐sectional studies show that Muslim youth in Western Europe are as religious or even slightly more religious than their first‐generation parents (Jacob & Kalter, 2013) and peers in the heritage countries (Güngör et al., 2012). Longitudinal studies investigating within‐person changes among Muslim youth are rare. A noticeable exception is Simsek et al. (2019) who showed that non‐immigrant and immigrant‐origin Christians in Western European countries became slightly less religious from age 15 to 17, whereas Muslim adolescents did not exhibit any significant changes in religiosity over the two‐year period. There was, however, variation around this average trajectory of stability among Muslim youth, which points toward *quantitative* differences between individuals. To shed light on this variation, the authors counted the number of Muslim youth who increased, decreased, or did not change over time. This approach is limited as it cannot speak to the level at which change occurs (e.g., decline at higher or lower levels of religiosity), the rate of change (e.g., gradual or sharp decline), or the functional form of change (e.g., linear or curvilinear). In sum, the study provides valuable insights but is limited by only covering a two‐year period and by its variable‐centered analytical approach that is less suited to detect *qualitatively* different developmental trajectories.

Taken together, there is a lack of longitudinal studies investigating within‐person changes in religiosity among acculturating Muslim youth. The evidence that exists is mostly cross‐sectional and the few longitudinal studies cover a limited period of adolescence. We thus do not know whether the declines in religiosity during adolescence and beyond often observed in North American Christian samples apply to other religious groups in different national contexts. Theories and findings suggest that declines in religiosity are less common among Muslim youth in Western societies while stability at higher levels or even increases in religiosity are likely.

## Developmental outcomes of religiosity

Religiosity can provide a protective resource for youths' emotional well‐being and a buffer against risky and unhealthy behavior (Yonker et al., 2012). The positive effects of religiosity are however bound to the value of religiosity in a societal context. In more secularized countries such as Germany, the protective effect of religiosity for Christians is considerably weaker than commonly observed in the United States (Hodapp & Zwingmann, 2019), and the protective effect of religiosity for Muslim youth might be further weakened because of populist public debates typically portraying Muslim people as a cultural threat. The empirical evidence for protective effects among Muslim individuals is mixed and largely based on cross‐sectional associations. Greater religiosity among Muslim individuals has, for example, been linked to lower acculturative stress (Goforth et al., 2014), higher psychological well‐being (Dimitrova & Aydinli‐Karakulak, 2016; Stuart & Ward, 2018), and fewer externalizing problems (Balkaya et al., 2019), but also to lower levels of well‐being (Friedman & Saroglou, 2010; Oberoi & Trickett, 2018) and higher levels of problem behavior (Maes et al., 2014).

Furthermore, religions provide direction and guidance in various life domains including people's attitudes toward sexual liberties (e.g., abortion, homosexuality) and gender roles. While most religions endorse more traditional values, they differ in how strongly they emphasize these values. Muslim individuals, for example, exhibit on average more traditional gender role values (Kretschmer, 2018) and oppose sexual liberties more strongly (e.g., Eskelinen & Verkuyten, 2020) than members of other religious groups in Western Europe.

Finally, religiosity may be associated with immigrant‐origin youth's acculturation processes. This includes belongingness (e.g., national identification), cultural preferences (e.g., attitudes toward heritage culture maintenance), and social ties (e.g., friendships with members of the majority group). While in the United States, religious engagement has been linked to better integration outcomes, in Western Europe it is considered an integration barrier (Foner & Alba, 2008). Accordingly, higher religiosity among Muslim youth has been linked to greater heritage culture maintenance and lower levels of national culture adoption (Dimitrova & Aydinli‐Karakulak, 2016; Friedman & Saroglou, 2010; Goforth et al., 2014).

## The present study

Our research aims were threefold. First, we sought to characterize religious development from adolescence to early adulthood among immigrant‐origin Muslim, immigrant‐origin Christian, and non‐immigrant Christian youth, thus uncovering long‐term religious trajectories. We expected to observe low levels and declines in religiosity among non‐immigrant Christian youth, slightly higher levels and weaker declines among immigrant‐origin Christian youth, and consistently high levels of religiosity among immigrant‐origin Muslim youth.

Second, we wanted to explore if there were classes (subgroups) of adolescents following different developmental trajectories, in order to identify and understand any meaningful heterogeneity between and within religious groups. We expected to find subgroups of adolescents within each group following distinct developmental trajectories. Specifically, we expected to find both decline and stability at comparatively low levels in the Christian samples, and stability, decline, and possibly increases at comparatively high levels in the Muslim sample.

Third, we aimed to assess whether class membership was associated with youth's trajectories of well‐being, behavior, values, and acculturation. We expected to observe weak positive links between religiosity and adolescents' well‐being trajectories in the Christian samples, a negative link between religiosity and involvement in risky and unhealthy behavior in all samples, a positive link between religiosity and traditional cultural values in all samples (with Muslim youth scoring higher on traditionalism), and a negative link between religiosity and sociocultural integration among immigrant‐origin youth.

## METHODS

### Procedure

We used three waves of data from the Children of Immigrants Longitudinal Survey in Four European Countries (CILS4EU, Kalter et al., 2016). CILS4EU is a long‐term project that studies the structural, social, and cultural integration of immigrant‐origin youth in Germany, Sweden, England, and the Netherlands using representative samples of adolescents. We extended the time frame by focusing on Germany where four additional waves of data were collected (CILS4EU‐DE, Kalter et al., 2021). Data were collected at the following time points: Wave 1 in 2010, Wave 2 in 2011, Wave 3 in 2013, Wave 4 in 2014, Wave 5 in 2015, Wave 6 in 2016, and Wave 7 in 2018. The German sample comprised 5013 adolescents, initially in 9th grade, nested in 271 classrooms and 144 schools. Adolescents were recruited through a school‐based sample selection design that oversampled schools with a high proportion of immigrant‐origin adolescents. Although a refreshment sample was added to the panel in Wave 6, our analyses were based exclusively on the original panel sample because of our interest in developmental trajectories from adolescence to early adulthood. Participation rates in the original recruitment were high (school participation = 84%; class participation = 99%; student participation = 85%). At Waves 1 and 2, adolescents participated in school. From Wave 3 onward, they were followed up individually and interviewed via phone, mail, or web.

### Sample description

In light of our research aims, we excluded 28 adolescents with no information on their religious affiliation, 497 adolescents who indicated “no religion” when asked about their religious group, 223 adolescents who indicated “other religion” (including Buddhism, Hinduism, Judaism, and Sikhism), and 173 adolescents who reported different religious affiliations across time (e.g., Muslim at Wave 1 and Christian at Wave 3). We kept adolescents with missing information on up to five out of six measurement occasions and those who changed from a religious group to no religion or vice versa, which can be part of religious development. Finally, we excluded 12 adolescents who could not be categorized as non‐immigrant or immigrant‐origin due to missing data. This resulted in a final sample of 4080 adolescents. Gender, which was only asked with a binary item, was equally distributed (49.5% male, 49.8% female, 0.7% missing). The average age in years across waves was: *M*_*W*1_ = 15.25, *SD*_*W*1_ = 0.68, *M*_*W*2_ = 16.19, *SD*_*W*2_ = 0.68, *M*_*W*3_ = 17.36, *SD*_*W*3_ = 0.70, *M*_*W*4_ = 18.35, *SD*_*W*4_ = 0.65, *M*_*W*5_ = 19.41, *SD*_*W*5_ = 0.66, *M*_*W*6_ = 20.69, *SD*_*W*6_ = 0.71, *M*_*W*7_ = 22.49, *SD*_*W*7_ = 0.64. For the main analyses, we distinguished between non‐immigrant Christian youth (*n* = 2086), immigrant‐origin Christian youth (*n* = 797), and immigrant‐origin Muslim youth (*n* = 1197; 98.2% immigrant‐origin). Immigrant‐origin refers to adolescents who were born abroad or had at least one parent born abroad. Immigrant‐origin Christian youth originated from 85 different countries with Poland, Russia, and Italy accounting for 49.8% of the sample. Immigrant‐origin Muslim youth originated from 47 countries with Turkey alone accounting for 65.5% of the sample.

### Measures

#### Religious self‐categorization

Religious self‐categorization was assessed with a single item “What is your religion?” at Waves 1–3 and 5–7. The response options were 1 (*No religion*), 2 (*Buddhism*), 3 (*Christianity*), 4 (*Christianity: Catholic*), 5 (*Christianity: Protestant*), 6 (*Hinduism*), 7 (*Islam*), 8 (*Judaism*), 9 (*Sikhism*), and 10 (*Other religion*).

#### Religiosity

Religiosity was assessed with three items capturing its key components (i.e., public involvement, private practices, importance). We combined these widely used items and examined overall religiosity which—according to meta‐analyses—is the most common approach to studying religiosity (e.g., Hardy et al., 2019; Yonker et al., 2012). The first item, “How important is religion to you?”, was assessed at Waves 1–7. The response options were 1 (*Very important*), 2 (*Fairly important*), 3 (*Not very important*), and 4 (*Not at all important*). The second item, “How often do you visit a religious meeting place (e.g., a church, a mosque, a synagogue or a temple)?”, was assessed at Waves 1–3 and 5–7. The response options were 1 (*Never*), 2 (*Occasionally (but less than once a month)*), 3 (*At least once a month*), 4 (*At least once a week*), and 5 (*Every day*). The third item, “How often do you pray?”, was assessed at Waves 1–3 and 5–7. The response options were 1 (*Never*), 2 (*Occasionally (but less than once a month)*), 3 (*At least once a month*), 4 (*At least once a week*), 5 (*One to four times a day*), and 6 (*Five times a day or more*). We rescaled the first and third items to a five‐point scale with higher values indicating greater religiosity. Across time, the items loaded on a single factor explaining between 73.8% and 77.8% of the variance. Cronbach's α across waves ranged from .82 to .85. We computed mean scores for Waves 1–3 and 5–7.

#### Psychological well‐being

##### Anxiety and depression

Anxiety and depression were measured with two items each at Waves 1, 3, and 7 (depression was additionally measured at Wave 2). The items were: “How often are each of these statements true about you? I feel very… 1) worried, 2) anxious, 3) depressed, 4) worthless.” The response options were 1 (*Often true*), 2 (*Sometimes tru*e), 3 (*Rarely true*), and 4 (*Never true*). The items were rescaled so that higher values indicate greater anxiety or depressive symptoms. The anxiety (*r*s ≥ .35, *p*s ≤ .001) and depression (*r*s ≥ .49, *p*s ≤ .001) items were positively correlated across waves. We computed mean scores for both.

##### Life satisfaction

Life satisfaction was captured with one item at Waves 1–7: “On a scale from 1 to 10 where 1 is very unsatisfied and 10 is very satisfied, how satisfied are you with your current situation.”

##### Health

Health was measured with a single item at Waves 1, 2, 4, and 6: “How good is your health compared to others of your age?”. The response options were: 1 (*Very good*), 2 (*Good*), 3 (*About the same*), 4 (*Bad*), and 5 (*Very bad*). The item was rescaled, so that higher values indicate better general health.

#### Risk behavior

Drinking and smoking were captured with single items at Waves 1–5 and 7. The items were: “How often do you drink alcohol?” and “How often do you smoke cigarettes?”. Drug use was assessed with one item at Waves 1, 2, 4, 5, and 7: “How often do you use drugs (e.g., hash, paddos, ecstasy pills)?”. The response options ranged from 1 (*Every day*), 2 (*Once or several times a week*), 3 (*Once or several times a month*), 4 (*Less often*), to 5 (*Never*). The items were rescaled, so that higher values indicate more risk behavior.

#### Cultural values

##### Gender role values

Gender role values were assessed with four items at Waves 1, 2, 4, and 6. The items were: “In a family, who should do the following? 1) Take care of the children, 2) Cook, 3) Earn money, 4) Clean the house.” The response categories included 1 (*Mostly the man*), 2 (*Mostly the woman*), and 3 (*Both about the same*). Traditional responses (i.e., mostly women clean, cook, take care of children, and mostly men earn money) were given a score of 0, less traditional (more egalitarian) responses (i.e., mostly men or both about the same clean, cook, take care of the children, and mostly women or both about the same earn money) a score of 1. Cronbach's α across waves ranged from .64 to .70. We computed sum scores which ranged from 0 to 4, with higher values indicating less traditional gender role values.

##### Tolerance of sexual liberties

Tolerance of sexual liberties was assessed with four items at Waves 1, 3, 5, and 7. Adolescents were asked: “Do you think the following are “always OK”, “often OK” “sometimes OK” or “never OK”? 1) Living together as a couple without being married, 2) Divorce, 3) Abortion, 4) Homosexuality.” The response options were 1 (*Always OK*), 2 (*Often OK*), 3 (*Sometimes OK*), and 4 (*Never OK*). The items were recoded so that higher values indicated greater tolerance. Cronbach's α across waves ranged from .72 to .78. Composite scores were computed based on means.

#### Acculturation

##### Attitudes toward culture adoption and maintenance

Attitudes toward German culture adoption and heritage culture maintenance were assessed with a single item each at Waves 1–3, 5, and 7. The adoption item was: “Immigrants should adapt to German society”; the maintenance item was: “Immigrants should do all they can to keep their customs and traditions.” The response options were 1 (*Strongly agree*), 2 (*Agree*), 3 (*Neither agree nor disagree*), 4 (*Disagree*), and 5 (*Strongly disagree*). The items were recoded so that higher values indicate a stronger preference for immigrants' adoption or maintenance.

##### German friends

The number of German friends was assessed with a single item at Waves 1–7. The item was: “Thinking now about all of your friends, how many of them have a German background?”. The response options ranged from 1 (*Almost all or all*), 2 (*A lot*), 3 (*About half*), 4 (*A few*), to 5 (*None or very few*). The item was recoded so that higher values indicate more German friends.

##### National identification

National identification was assessed with a single item at Waves 1–7. The item was: “How strongly do you feel German?”. Response options ranged from 1 (*Very strongly*), 2 (*Fairly strongly*), 3 (*Not very strongly*) to 4 (*Not at all strongly*). The item was recoded so that higher values indicate stronger national identification.

#### Parent religiosity

Parent religiosity was assessed with a single item at Wave 1. Parents responded to the question “How important is religion to you?”. The response options included 1 (*Very important*), 2 (*Fairly important*), 3 (*Not very important*), and 4 (*Not at all important*). The item was rescaled so that higher values indicate greater parental religiosity.

#### Demographic variables

##### Gender

Gender was measured at Waves 1–3 and 5–7 with a binary single item: “Are you a boy or a girl?” The response options were 1 (*boy*) and 2 (*girl*). If information on gender was missing, it was imputed based on the next available wave. The item was recoded into 0 (boy) and 1 (girl).

##### Age

Age in years was computed based on three items: 1) “When were you born? Year,” 2) “When were you born? Month,” and 3) “Date of interview: Day, month, year.” If information was missing, it was imputed based on the next available wave. Items 1 and 2 were assessed at Waves 1–3 and 5–7. Item 3 was recorded at Waves 1–7.

##### Mother and father education

Mother and father education were assessed with three items each at Waves 1–3 and 6. The items were: “Did your mother/father complete… 1) primary school (or a similar foreign education), 2) secondary school (or a similar foreign education), 3) university?”. Response options were 1 (*yes*) and 2 (*no*). If responses were missing, information was imputed based on the next available wave. We recoded mothers' and fathers' education into 1 (*primary education*), 2 (*secondary education*), and 3 (*tertiary education*).

##### Socioeconomic status

Socioeconomic status (SES) was assessed at Waves 1, 3, and 6 using both mothers' and fathers' International Socio‐Economic Index of occupational status (ISEI). The ISEI variables were constructed by the CILS4EU team. The variable combines income and education to capture the status of an occupation (De Ganzeboom et al., 1992) and is widely used in international large‐scale studies, such as PISA. In case of missing information, we used ISEI values from the next available wave. For SES, we used parents' highest ISEI score.

##### Immigrant generation

Immigrant generation is based on a generational status variable constructed by the CILS4EU team using information on child, parent, and grandparent's place of birth. We imputed missing values based on later waves and recoded the generational status variable to distinguish between 1 (*first generation*), 2 (*second generation*), and 3 (*third generation*) immigrant adolescents.

#### Attrition and missing data

As in many longitudinal studies, there was attrition over time. Of the final sample (at W1), 82.5% participated at W2, 67.4% at W3, 60.2% at W4, 55.7% at W5, 45.4% at W6, and 38.5% at W7. To examine if attrition was systematic, we compared adolescents who did versus did not participate in later waves on demographic characteristics, religiosity, and the developmental outcomes. Attrition was unrelated to youth's religiosity but some demographic characteristics, youth's cultural values, and acculturation had significant, albeit small, effects on dropout. Detailed findings and an examination of non‐responses can be found in the supplementary materials (OSM S2). As recommended, we used Full Information Maximum Likelihood (FIML) estimation to handle missing data (Enders & Bandalos, 2001).

### Statistical analyses

Our analyses were conducted in three steps. First, we used LGCM to examine which type of growth function (e.g., linear, quadratic, piecewise) fit the data best and described the overall changes in religiosity separately for each religious group based on this optimal growth function. Second, we estimated GMMs (for an introduction see Jung & Wickrama, 2008) for each religious group to uncover if there were classes (i.e., subgroups) of adolescents with qualitatively different trajectories. Third, we linked class membership to demographic characteristics, parent religiosity, and longitudinal changes in the outcomes. We ran the analyses separately for each religious group because of cross‐cultural and contextual variation in the meaning of religious importance and behavior. Our analyses were conducted in MPlus v8.7 (Muthén & Muthén, 1998‐2017). We used maximum likelihood estimation with robust standard errors (MLR) to account for non‐normality. Model fit was assessed using goodness‐of‐fit indices, including a Root Mean Square Error of Approximation (RMSEA) of less than .05, Comparative Fit Index (CFI) above .90, Tucker‐Lewis index (TLI) greater than .95, and Standardized Root Mean square Residual (SRMR) of less than .08.

## RESULTS

Descriptive statistics are presented in Table A1. The correlations between the study variables can be found in the supplementary materials (OSM S1).

### Overall religious development

Curran and colleagues highlighted that a critical first step in any growth model is to identify the optimal functional form of a growth trajectory because an incorrect functional form would lead to biased results once the model is extended (Curran et al., 2010). To identify this optimal functional form, we first estimated six different growth models separately for each religious group: a linear model, a quadratic model, a piecewise model with two intercepts, two piecewise models with a turning point at either Wave 3 or 5 (religiosity was not assessed at Wave 4), and a latent basis model. A linear change would indicate a continuous change in mean levels of religiosity over time. A quadratic change would indicate that the rate of change differs across time (e.g., decreases following increases). Piecewise models allow us to directly compare changes during adolescence with changes during early adulthood. Latent basis models are flexible non‐linear models with free time scores, in which the mean of the slope growth factor describes an average linear change from one time point to another. A more detailed description of the growth models, model fit indices, and fit comparisons can be found in the supplementary materials (OSM S3).

**FIGURE 1:**
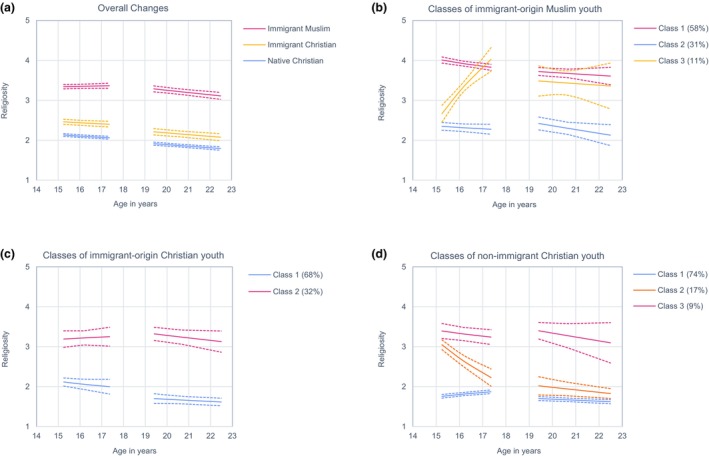
Overall changes in religiosity for each religious group and classes of religious development within each religious group. Dashed lines show 95% confidence intervals.

The model fit indices indicated that the piecewise growth model with two intercepts fit the data well for all three religious groups, χ^2^(7) ≤ 53.95; CFI ≥ .986; TLI ≥ .970; RMSEA ≤ .057; SRMR ≤ .029. The results of adjusted χ^2^ difference tests further showed that this model fit better than all alternative models, χ^2^ (*df*) ≥ 10.90 (5), *p* ≤ .053, indicating that this was the optimal functional form to describe changes in religiosity in this dataset. The piecewise growth model with two intercepts estimates four growth factors: an intercept for adolescence (i.e., initial level of religiosity at Wave 1), a slope for adolescence (i.e., linear rate of change from Wave 1 to Wave 3), an intercept for early adulthood (i.e., initial level of religiosity at Wave 5), and a slope for early adulthood (i.e., linear rate of change from Wave 5 to Wave 7). The results are shown in Panel (a) of Figure 1.

For immigrant‐origin Muslim youth, religiosity was on average medium at age 15 (*b*_1_ = 3.34, *SE* = 0.03, *p* < .001), stable during adolescence (*m*_1_ = 0.09, *SE* = 0.11, *p* = .407), medium at age 18 (*b*_2_ = 3.29, *SE* = 0.04, *p* < .001), and decreased during early adulthood (*m*_2_ = −0.46, *SE* = 0.11, *p* < .001).^1^ For both immigrant‐origin and non‐immigrant Christian youth, religiosity was low at age 15 (immigrant‐origin: *b*_1_ = 2.46, *SE* = 0.03, *p* < .001; non‐immigrant: *b*_1_ = 2.13, *SE* = 0.02, *p* < .001), decreased during adolescence (immigrant‐origin: *m*_1_ = −0.23, *SE* = 0.12, *p* = .046; non‐immigrant: *m*_1_ = −0.27, *SE* = 0.07, *p* < .001), was low at age 19 (immigrant‐origin: *b*_2_ = 2.21, *SE* = 0.04, *p* < .001; non‐immigrant: *b*_2_ = 1.91, *SE* = 0.02, *p* < .001), and decreased during early adulthood (immigrant‐origin: *m*_2_ = −0.36, *SE* = 0.11, *p* = .001; non‐immigrant: *m*_2_ = −0.32, *SE* = 0.05, *p* < .001).

To examine if specific growth factors (e.g., the intercepts for adolescence and early adulthood) were statistically different, we carried out Wald tests with one degree of freedom using the Model Test command of Mplus. A comparison of the intercepts showed that Christian youth were more religious at age 15 than 19 (immigrant‐origin: *W* = 48.18, *p* < .001; non‐immigrant: *W* = 139.85, *p* < .001) while Muslim youth were as religious at age 15 as they were at age 19 (*W* = 2.40, *p* = .122). A comparison of the slopes showed that religiosity decreased similarly during adolescence and early adulthood among Christia youth (immigrant‐origin: *W* = 0.60, *p* = .440; non‐immigrant: *W* = 0.38, *p* = .540) and that religiosity changed more strongly during early adulthood than adolescence among Muslim youth (*W* = 11.99, *p* = .001).

### Classes of religious development

We used GMM to identify distinct classes of religious development and employed a stepwise procedure, whereby one additional class (k) was added to the model at a time. At each step, we compared the fit of the models with and without the additional class using the Bayesian Information Criterion (BIC; lower values indicate better fit), the Lo–Mendell–Rubin Likelihood Ratio Test (LMR‐LRT), and the Bootstrapped Likelihood Ratio Test (BLRT). Significant LMR‐LRTs and BLRTs indicate that the higher‐class solution fits better than the lower‐class solution. Solutions in which classes contained 5% of the total sample or less were not considered. The model fit statistics of the GMMs and class sizes are shown in Table 1.

**TABLE 1:** Model fit statistics of Growth Mixture Models and class sizes for immigrant‐origin Muslim, immigrant‐origin Christian, and non‐immigrant Christian youth.

C	BIC	LMR–LRT	BLRT	Entropy	Class sizes in %
Immigrant Muslims					
2	9377.48	−4664.10**	−4664.10***	0.697	67, 33
3	9338.94	−4600.14*	−4600.14***	0.712	58, 31, 11
4	9333.00	−4563.16	−4563.16***	0.747	2, 31, 10, 57
Immigrant Christian					
2	6232.29	−3085.78*	−3085.78***	0.611	68, 32
3	6219.62	−3046.01	−3046.01***	0.659	65, 29, 6
Non‐immigrant Christians					
2	14,884.65	−7453.72***	−7453.72***	0.755	84, 16
3	14,796.39	−7346.79*	−7346.79***	0.738	74, 9, 17
4	14,717.16	−7283.55	−7283.55***	0.764	71, 3, 12, 14

*Note*: Class sizes are reported based on the estimated posterior probabilities. Higher‐class solutions inadmissible. C = Classes. For immigrant‐origin Christians, we fixed the insignificant within‐class variance of the early adulthood slope to 0.

* *p* < .05;

** *p* < .01;

*** *p* < .001.

#### Immigrant‐origin Muslim youth

For immigrant‐origin Muslim youth, BIC and BLRT indicated a 4‐class solution, which we rejected because one class comprised only 2% of the sample. The LMR‐LRT pointed toward a 3‐class solution. BIC and BLRT also indicated that a 3‐class solution was better than a 2‐class solution. We thus settled on the 3‐class solution.

The three classes of religious development for immigrant‐origin Muslim youth are depicted in Panel B of Figure 1. The largest class (58%) was characterized by high religiosity at age 15 (*b*_1_ = 4.01, *SE* = 0.04, *p* < .001), decreasing religiosity during adolescence (*m*_1_ = −0.70, *SE* = 0.18, *p* < .001), high religiosity at age 19 (*b*_2_ = 3.72, *SE* = 0.05, *p* < .001), and stability during early adulthood (*m*_2_ = −0.30, *SE* = 0.32, *p* = .357). For simplicity, we labeled this class “high religiosity.” A comparison of the intercepts showed that the levels of religiosity in adolescence and early adulthood were significantly different (*W* = 31.01, *p* < .001). A comparison of the slopes showed that changes in religiosity during adolescence and early adulthood did not significantly differ (*W* = 1.42, *p* = .233). The second class of immigrant‐origin Muslim youth (31%) was characterized by low religiosity at age 15 (*b*_1_ = 2.35, *SE* = 0.05, *p* < .001), stability during adolescence (*m*_1_ = −0.28, *SE* = 0.22, *p* = .208), low religiosity at age 19 (*b*_2_ = 2.42, *SE* = 0.08, *p* < .001), and decreases during early adulthood (*m*_2_ = −0.77, *SE* = 0.38, *p* = .046). We labeled this class “low religiosity.” A comparison of the intercepts and slopes showed that the levels and changes in adolescence and early adulthood did not differ (*W*_intercepts_ = 1.03, *p* = .311, *W*_slopes_ = 1.05, *p* = .305). Finally, the third and smallest class of immigrant‐origin Muslim youth (11%) was characterized by medium religiosity at age 15 (*b*_1_ = 2.67, *SE* = 0.11, *p* < .001), sharp increases during adolescence (*m*_1_ = 5.23, *SE* = 0.89, *p* < .001), medium to high religiosity at age 19 (*b*_2_ = 3.48, *SE* = 0.19, *p* < .001), and stability during early adulthood (*m*_2_ = −0.33, *SE* = 0.96, *p* = .732). We labeled this class “increasing religiosity.” Adolescents in the “increasing religiosity” class were more religious at age 19 than they were at age 15 (*W* = 13.04, *p* < .001). The comparison of the slopes showed that adolescence was characterized by stronger developmental changes than early adulthood (*W* = 14.27, *p* < .001).

To confirm distinctiveness, we compared the growth factors across the three classes of religious development. A comparison of the adolescence intercepts showed that religiosity at age 15 was higher in the high than the increasing and low religiosity classes (*W* = 215.28, *W* = 633.27, respectively, *p*s < .001), and higher in the increasing than the low religiosity class (*W* = 5.81, *p* = .016). A comparison of the adolescence slopes showed similar changes in religiosity in the high and low religiosity classes (*W* = 1.71, *p* = .191) and stronger changes in religiosity in the increasing religiosity class (*W* = 53.23, *W* = 39.35, respectively, *p*s < .001). A comparison of the early adulthood intercepts showed that the increasing religiosity class was as religious as the high religiosity class at age 19 (*W* = 1.22, *p* = .270). Both the high and increasing religiosity classes were more religious than the low religiosity class (*W* = 170.68 and *W* = 27.69, respectively, *p*s < .001). A comparison of the early adulthood slopes indicated similarly strong changes in religiosity across the three classes (*p*s ≥ .478). Together these findings point toward three distinct groups of Muslim adolescents characterized by either low, increasing, or high levels of religiosity.

#### Immigrant‐origin Christian youth

For immigrant‐origin Christian youth, BIC and BLRT indicated a 3‐class solution, whereas the LMR‐LRT indicated a 2‐class solution. The 3‐class solution contained a small class with 6% of the immigrant‐origin Christian sample that is 50 adolescents (before attrition). While this is above our cut‐off criteria of 5%, we considered this class too small for further analyses and focused on the 2‐class solution, which was supported by the LMR‐LRT. The 2‐class solution is shown in Panel (c) of Figure 1.

Most immigrant‐origin Christian youth (68%) were characterized by low religiosity at age 15 (*b*_1_ = 2.12, *SE* = 0.05, *p* < .001), stability during adolescence (*m*_1_ = −0.45, *SE* = 0.30, *p* = .127), low religiosity at age 19 (*b*_2_ = 1.70, *SE* = 0.06, *p* < .001), and stability during early adulthood (*m*_2_ = −0.22, *SE* = 0.16, *p* = .153). We labeled this class “low religiosity.” A comparison of the intercepts showed that adolescents in the “low religiosity” class were less religious at age 19 than they were at age 15 (*W* = 51.59, *p* < .001). A comparison of the slopes indicated that changes in religiosity during adolescence were similar to changes in religiosity during early adulthood (*W* = 0.31, *p* = .576). The second class of immigrant‐origin Christian youth (32%) was characterized by medium religiosity at age 15 (*b*_1_ = 3.19, *SE* = 0.11, *p* < .001), stability during adolescence (*m*_1_ = 0.22, *SE* = 0.53, *p* = .671), medium religiosity at age 19 (*b*_2_ = 3.32, *SE* = 0.08, *p* < .001), and stability during early adulthood (*m*_2_ = −0.51, *SE* = 0.32, *p* = .110). We labeled this class “medium religiosity.” A comparison of the intercepts showed that adolescents in the medium religiosity class were as religious at age 15 as they were at age 19 (*W* = 1.99, *p* = .158). A comparison of the slopes revealed similar changes in religiosity during adolescence and early adulthood (*W* = 0.93, *p* = .334).

Comparing the growth factors across the two classes of religious development showed that adolescents in the medium religiosity class were more religious at age 15 and 19 than adolescents in the low religiosity class (*W* = 75.19, *p* < .001 and *W* = 433.28, *p* < .001). The slopes for adolescence (*W* = 0.73, *p* = .392) and early adulthood (*W* = 0.46, *p* = .496) were similar. Together these findings point toward two distinct groups of immigrant‐origin Christian adolescents with either low or medium levels of religiosity.

#### Non‐immigrant Christian youth

For non‐immigrant Christian youth, BIC and BLRT pointed toward a 4‐class solution, which we rejected because of one small class containing only 3% of the sample. The LMR‐LRT pointed toward a 3‐class solution and BIC and BLRT also indicated that a 3‐class solution fit better than a 2‐class solution. So we settled on the 3‐class solution shown in Panel D of Figure 1.

The largest class (74%) was characterized by low religiosity at age 15 (*b*_1_ = 1.76, *SE* = 0.02, *p* < .001), increases during adolescence (*m*_1_ = 0.44, *SE* = 0.09, *p* < .001), low religiosity at age 19 (*b*_2_ = 1.70, *SE* = 0.03, *p* < .001), and decreases during early adulthood (*m*_2_ = −0.21, *SE* = 0.07, *p* = .004). We labeled this class “low religiosity.” A comparison of the intercepts showed that adolescents in the “low religiosity” class were marginally more religious at age 15 than they were at age 19 (*W* = 4.30, *p* = .038). A comparison of the slopes further pointed toward stronger changes in religiosity during adolescence (*W* = 27.81, *p* < .001). The second class (17%) was characterized by medium religiosity at age 15 (*b*_1_ = 3.05, *SE* = 0.06, *p* < .001), sharp decreases during adolescence (*m*_1_ = −3.16, *SE* = 0.41, *p* < .001), low religiosity at age 19 (*b*_2_ = 2.02, *SE* = 0.12, *p* < .001), and further decreases during early adulthood (*m*_2_ = −0.51, *SE* = 0.24, *p* = .034). We labeled this class “decreasing religiosity.” Comparing the intercepts showed that adolescents in the “decreasing religiosity” class were more religious at age 15 than they were at age 19 (*W* = 77.96, *p* < .001). Comparing the slopes showed that religiosity decreased more strongly during adolescence than early adulthood (*W* = 21.10, *p* < .001). The third class of non‐immigrant Christian youth (9%) was characterized by medium religiosity at age 15 (*b*_1_ = 3.39, *SE* = 0.10, *p* < .001), stability during adolescence (*m*_1_ = −0.59, *SE* = 0.34, *p* = .082), medium religiosity at age 19 (*b*_2_ = 3.40, *SE* = 0.11, *p* < .001), and stability during early adulthood (*m*_2_ = −0.79, *SE* = 0.54, *p* = .139). We labeled this class “medium religiosity.” A comparison of the intercepts showed that adolescents were as religious at age 15 as they were at age 19 (*W* = 0.01, *p* = .938). A comparison of the slopes indicated that the rates of change during adolescence and early adulthood were similar (*W* = 0.09, *p* = .763).

Comparing the growth factors across classes showed that the adolescent intercepts (*p*s ≤ .005) and slopes (*p*s ≤ .005) differed for all pairs of classes. This means that the three classes of religious development were distinct because religious change occurred at different levels and rates during adolescence. The early adulthood intercepts also differed for all pairs of classes (*p*s ≤ .006), but the early adulthood slopes were similar (*p*s ≥ .185). So, religiosity declined in each class during early adulthood to the same extent but at different levels. Together these findings indicate that the non‐immigrant Christian sample consists of three distinct subgroups characterized by low, medium, or decreasing levels of religiosity.

### Developmental outcomes of religiosity

To address our third research aim, we examined if class membership was associated with demographic characteristics, parent religiosity, and longitudinal changes in psychological well‐being, risky and unhealthy behavior, cultural values, and acculturation. To account for the imprecision of class membership, we used the BCH procedure for auxiliary variables measured at Wave 1 and the BCH procedure for 3‐step mixture modeling for variables measured at multiple occasions (Asparouhov & Muthén, 2021). We summarize the results in the following sections and report detailed findings in the supplementary materials (OSM S4).

#### Immigrant‐origin Muslim youth

For immigrant‐origin Muslim youth, the three classes of religious development did not differ in terms of immigrant generation, socioeconomic background (ISEI), father education, life satisfaction, smoking, and use of drugs. But differences emerged for gender, mother education, parent religiosity, anxiety, depression, health, alcohol consumption, cultural values, and acculturation (see OSM S4, Table D1).

In the high religiosity class, the number of girls and mother education was comparatively low while parent religiosity was high. Adolescents in the high religiosity class reported comparatively higher well‐being (e.g., lower anxiety and depression, better health) and were least likely to consume alcohol. They had more conservative cultural values (e.g., more traditional gender role values, lower tolerance of sexual liberties), and were less integrated (e.g., less in favor of immigrants adopting the German culture, fewer friends from the majority population, and weaker national identification) than adolescents in the other two classes.

In the low religiosity class, the number of girls and mother education was comparatively high while parent religiosity was low. Muslim‐identifying adolescents in the low religiosity class reported comparatively lower levels of well‐being (e.g., they were comparatively more anxious and depressed and reported poorer health) and were more likely to consume alcohol (at extremely low levels). The low religiosity class expressed less traditional attitudes and seemed better integrated into German society than adolescents in the other classes.

The increasing religiosity class fell in between the other two classes of religious development. Gender was more equally distributed and mother's education was neither comparatively low nor high. Adolescents in the increasing religiosity class scored comparatively high on well‐being and alcohol consumption. They were less traditional than the high religiosity class but more traditional than the low religiosity class. They endorsed heritage culture maintenance for immigrants more strongly than adolescents in the other classes and were more integrated into German society (e.g., comparatively strong support for German culture adoption among immigrants, more German friends, and higher national identification).

#### Immigrant‐origin Christian youth

For immigrant‐origin Christian youth, the two classes of religious development did not differ in terms of gender composition, parental education, psychological well‐being (i.e., anxiety, depression, and life satisfaction), and attitudes toward culture adoption (see OSM S4, Table D2). But differences emerged for immigrant generation, parents' occupational status, health, risky and unhealthy behavior, cultural values, and acculturation.

In the low religiosity class, the number of second‐generation immigrants and high‐status parents was higher while parent religiosity was lower than in the medium religiosity class. Adolescents in the low religiosity class also reported comparatively poorer health and engaged in more risky and unhealthy behavior (i.e., they consumed more alcohol, and showed stronger increases in smoking and in the use of drugs). The low religiosity class had less traditional gender role attitudes and was more tolerant of sexual liberties. They appeared more integrated into German society (e.g., adolescents had more German friends and identified more strongly as German), and, despite fluctuations, valued heritage culture maintenance less than the medium religiosity class.

#### Non‐immigrant Christian youth

For non‐immigrant Christian youth, the three classes of religious development did not differ in terms of father education, depressive symptoms, health, and gender role values. But the classes differed in terms of gender composition, mother education, parents' occupational status, parent religiosity, anxiety, life satisfaction, risky and unhealthy behavior, and tolerance of sexual liberties (see OSM S4, Table D3).

The low religiosity class comprised a comparatively high number of boys, lower educated mothers, lower social status parents, and less religious parents. Adolescents in the low religiosity class were characterized by increasing anxiety, comparatively lower life satisfaction, and more risky and unhealthy behavior (e.g., more drinking and smoking during adolescence and more drugs during early adulthood). The low religiosity class was more tolerant of sexual liberties than the medium religiosity class.

The medium religiosity class comprised a comparatively high number of girls, higher educated mothers, higher social status parents, and more religious parents. Adolescents in the medium religiosity class did not become more anxious over time and reported comparatively high satisfaction with life. They engaged less in risky and unhealthy behaviors (i.e., drinking, smoking, drugs) and were comparatively less tolerant of sexual liberties.

The decreasing religiosity class was characterized by increasing anxiety and lower levels of life satisfaction. At age 15, they reported relatively low engagement in risky and unhealthy behavior, but there were comparatively dramatic increases over time in drinking and smoking during adolescence and in the use of drugs during early adulthood. Adolescents in the decreasing religiosity class were comparatively more tolerant of sexual liberties.

## DISCUSSION

Immigration profoundly changed – and is still changing – the religious landscape of Western societies. Secularized countries such as Germany have a growing population of Muslim youth who are believed to maintain their religiosity across generations, showing little sign of assimilation in religiosity. This has raised important questions about the intricate links between religious development, sociocultural integration, and adjustment. Guided by a framework that contextualizes religious development, we examined changes in religiosity among immigrant‐origin Muslim, immigrant‐origin Christian, and non‐immigrant Christian youth during adolescence and early adulthood. We used a person‐oriented analytical approach to examine if there are subgroups of adolescents following different developmental trajectories and linked these religious trajectories to developmental outcomes such as psychological well‐being and risk behavior.

### Religious development during adolescence and early adulthood

Our initial findings—which did not look at subgroups of adolescents—showed that Muslim adolescents were on average more religious than Christian adolescents, although Muslim youths' religiosity was medium rather than exceptionally high. Both non‐immigrant and immigrant‐origin Christians became less religious during adolescence and beyond, indicating that they are in sync with their sociocultural context (Schnitker et al., 2021). In contrast, adolescence was characterized by stability among acculturating Muslim youth, as previously reported by Simsek et al. (2019). However, by extending the time frame, we showed that religiosity does decline among Muslim individuals, but the decline is delayed into early adulthood. Early adulthood thus seems to be a sensitive developmental period for religious change among acculturating Muslim youth. The delayed declines in religiosity could result from highly effective religious transmission in immigrant‐origin Muslim families (Jacob & Kalter, 2013) postponing religious decline until greater independence is experienced during early adulthood (Petts, 2009). Taken together, our findings indicate that religiosity declined among religiously affiliated young people in Germany, but the timing of the decline varied across religious groups.

### Heterogeneity in religious development

Behind those overall trends in religious development lies notable heterogeneity, which we captured by using a person‐oriented analytical approach. As expected, we found distinct classes of religious development in each religious group. Most Muslim adolescents (58%) were highly religious and experienced only small decreases in religiosity during adolescence and stability thereafter. This shows that religiosity is and continues to be important for the majority of Muslim youth in Western societies, which contrasts with the religious declines typically observed among North American Christian youth (King & Boyatzis, 2015; Namara Barry et al., 2010; Schnitker et al., 2021). It further corroborates theoretical claims that young people respond to stigmatization and discrimination with stronger ingroup identities to maintain a positive sense of self (e.g., Branscombe et al., 1999; Tajfel & Turner, 1979; Umaña‐Taylor et al., 2014). However, we also uncovered two other theoretically interesting classes of Muslim adolescents. The first (31%) scored relatively low on religiosity with no changes during adolescence followed by small decreases during early adulthood. Such consistently low levels of religiosity among Muslim youth in Western Europe have not been observed in prior research (Phalet et al., 2018, for a review). The size of this class contests prior notions of religiosity being unequivocally important for Muslim youth and points towards religious assimilation for a third of our Muslim sample.

We further detected a small class of Muslim individuals (11%) characterized by sharp increases in religiosity during adolescence, resulting in this class becoming indistinguishable from the high religiosity class by early adulthood. This class is of interest because it is in direct opposition to the religious declines normally observed in non‐Muslim samples, but in line with French et al. (2014) who found increases, albeit smaller, in religiosity among Muslim adolescents in a Muslim‐majority context (Indonesia). The rapid and sharp increase in religiosity during adolescence could be a sign of intensified exploration of and commitment to a salient feature of Muslim youth's identity (Peek, 2005) or a response to increasing religious commitments (e.g., prayer, fasting, attire) that become obligatory from puberty onwards. The increase in religiosity should not be interpreted as emerging fundamentalist religious beliefs (Verkuyten, 2018)as the analyses of the outcome variables (which we discuss below) provide no evidence for this. Importantly, the ability to uncover and measure these less common classes of Muslim adolescents highlights the value of person‐oriented analytical approaches.

The religious development of Christian youth did not follow a uniform path either. Most Christian adolescents (74% non‐immigrant and 68% immigrant‐origin) were characterized by continuously low levels of religiosity, despite some small fluctuations among the non‐immigrant sample (i.e., small increases during adolescence followed by small decreases during early adulthood). This indicates that religion is of little consequence for the majority of self‐identified Christian youth and mirrors the state of German society at large. It is also in line with prior person‐oriented research identifying groups of Christian adolescents with consistently low levels of religiosity (e.g., Goodman & Dyer, 2020; Petts, 2009; Wright et al., 2018). However, we also found a class with medium and stable religiosity in both Christian samples (9% non‐immigrant and 32% immigrant‐origin). This finding corroborates earlier studies (e.g., Goodman & Dyer, 2020; Petts, 2009) and emphasizes that religious decline is not a universal trend even in secularized societies. Among non‐immigrant Christians, we uncovered a third class of adolescents comprising 17% of the sample. This class was characterized by sharp declines in religiosity during adolescence and further decreases during early adulthood. The strong decline in adolescence could perhaps result from increasing freedom to explore and reevaluate the religious beliefs and practices acquired during childhood (King et al., 2021).

### Predictors and outcomes of religious development

We examined whether class membership was meaningfully related to factors including demographic characteristics and parent religiosity. Our findings point toward culture‐specific gender differences in religious development, with Muslim girls being overrepresented in the low religiosity class and Christian girls being overrepresented in the medium religiosity class. This asymmetric pattern has been documented before and can be explained by societal and religious norms (Schnabel, 2018) that emphasize, for example, the participation of Muslim boys in worship services but encourage Muslim girls to pray at home (Loewenthal et al., 2002). We further observed that immigrant generation was not related to class membership among Muslim adolescents, while Christian adolescents of higher immigrant generation were more likely to be in the low religiosity class. This is indicative of greater religious assimilation among Christian than Muslim youth (Molteni & van Tubergen, 2022). Finally, we observed similar effects of parent religiosity on class membership across religious groups, with children of more religious parents being overrepresented in the high and medium religiosity classes. Taken together, these findings show that demographic characteristics and parent religiosity predict youth's religious development.

To contribute to ongoing debates regarding religiosity, adjustment, and sociocultural integration, we examined whether class membership was related to long‐term changes in psychological well‐being, risky and unhealthy behavior, cultural values, and sociocultural integration. For immigrant‐origin Muslim adolescents, high religiosity was linked to better well‐being (including lower risk behavior) and lower levels of sociocultural integration (including more conservative attitudes), whereas low religiosity was linked to worse well‐being and higher levels of sociocultural integration. Muslim adolescents in the increasing religiosity class reported both positive well‐being and sociocultural integration while also endorsing that immigrants additionally maintain their heritage culture. This class demonstrates that increasing religiosity can occur alongside moderately liberal attitudes and successful sociocultural integration. From a developmental point of view, the differences between adolescents in the high and increasing religiosity classes are especially interesting as they show that developmental trajectories (i.e., how one came to be religious), rather than outcomes (i.e., how religious one is as a young adult), matter for Muslim youth's well‐being and acculturation (Spiegler et al., 2019).

Similar key patterns were found for Christian adolescents. Immigrant‐origin Christian youth in the medium religiosity class were healthier, less engaged in risky behavior, more traditional, and less acculturated than those in the low religiosity class. For non‐immigrant Christian youth, medium religiosity was also linked to better well‐being, less risky and unhealthy behavior, and more traditional attitudes. Non‐immigrant Christian adolescents in the decreasing religiosity class seemed overall similar to those in the low religiosity class. These results show that religiosity is a developmental resource for Muslim and Christian adolescents but is difficult to reconcile with liberal attitudes and sociocultural integration.

Our findings further indicate that the links between religiosity, well‐being, behavior, and cultural values vary across religious groups. Religiosity was, for example, more consistently linked to the well‐being trajectories of Muslim youth. As a result, religious Muslim adolescents were better off in terms of psychological well‐being than religious Christian adolescents. This suggests that religiosity is a more important developmental resource for acculturating Muslim youth than other immigrant‐origin and non‐immigrant youth in Germany. This finding is in line with identity development frameworks (e.g., Umaña‐Taylor et al., 2014) and social psychological theories (e.g., Branscombe et al., 1999) that draw attention to the benefits of strong identities as a buffer against discrimination. Moreover, religious development was more consistently linked to the risky and unhealthy behavior of Christian youth, as less religious Christian adolescents were less likely to abstain from such behaviors than less religious Muslim adolescents. This underscores that Islam provides more proscriptive norms for behavioral conduct than Christianity (Ghandour et al., 2009). Finally, religious development was more consistently related to the attitudes of Muslim than Christian youth, as religious Christian adolescents were more likely to have liberal attitudes than religious Muslim adolescents. This suggests that Islam is more difficult to reconcile with liberal attitudes toward gender and sexuality than Christianity (Lewis & Kashyap, 2013). The links between religiosity and sociocultural integration were similar for Christian and Muslim immigrant youth indicating that high levels of religiosity—rather than being Muslim—may present a barrier to integration in Europe (Foner & Alba, 2008).

### Strengths and limitations

One key strength of the present research was the seven‐year longitudinal design, which enabled us to effectively examine developmental trajectories of religiosity from adolescence into early adulthood. In so doing, we could identify an overall reduction in Muslim youths' religiosity during early adulthood, which was hitherto unobserved in prior research (e.g., Simsek et al., 2019). We further used a rigorous analytical approach (GMM) to uncover developmental heterogeneity and approximate the number of adolescents with more or less normative developmental paths. This approach enabled us to identify, for example, a subgroup of Muslim adolescents with consistently low levels of religiosity, thus challenging the belief that religiosity is unequivocally important for Muslim youth. Another key strength concerns the examination of three religious groups, which allowed for comparisons of Muslim and Christian and immigrant‐origin and non‐immigrant youth. These comparisons shed light on culture‐specific developmental changes and religious assimilation, such as the identification of a Christian subgroup with decreasing (but no subgroup with increasing) religiosity and a Muslim subgroup with increasing (but no subgroup with decreasing) religiosity. Moreover, we focused on immigrant‐origin Muslim youth, an understudied minority population in developmental science. Understanding how Muslim youth relate to their religion and how changes in religiosity relate to other life domains is vital for an informed discussion of youth's sociocultural integration.

The limitations of our study—which signal prime directions for future research—include the use of a composite score for religiosity. Future studies could explore if similar trajectory classes emerge when religious service attendance, prayer, and identity are analyzed individually. Moreover, some of the outcome measures were brief (e.g., a two‐item measure of anxiety). Using longer measures with better psychometric properties would be a more desirable approach for future research. Finally, we examined religious development in the context of Germany. Our findings might be generalizable to other Western European countries with similar immigration histories, but whether the findings can be generalized within and beyond such contexts remains an open question for future research.

### Conclusion

Research on religious change during the formative years of adolescence and early adulthood is predominantly based on Christian samples in North America, short developmental timeframes, and analytical approaches less suited to uncover heterogeneity within populations. This has raised important questions regarding the generalizability of findings, long‐term religious trajectories, and potential developmental heterogeneity. We addressed these concerns by studying religious development among immigrant‐origin Muslim, immigrant‐origin Christian, and non‐immigrant Christian youth from age 15 to 22 using a person‐oriented analytical approach. Our findings indicate that the religious declines commonly observed among Christian adolescents in North America cannot be generalized to acculturating Muslim youth in Germany. However, religious declines among Muslim youth might occur later, in early adulthood. Most importantly, we identified subgroups of Muslim adolescents following distinct religious trajectories. By considering developmental heterogeneity, we uncovered a sizeable proportion of Muslim adolescents with consistently low levels of religiosity. This challenges common beliefs and indicates notable assimilation in religiosity. The existence of another subgroup, albeit small in numbers, further suggests that increasing religiosity during adolescence must not necessarily come at the cost of Muslim youths' sociocultural integration. Despite the largely positive implications for psychological well‐being, both Muslim and Christian youth struggled to combine religiosity with liberal cultural attitudes. We conclude that a focus on often overlooked national contexts and religious groups, long developmental timeframes, and person‐oriented analytical approaches are needed to fully understand religious development and its implications for youth's integration and well‐being.

## FUNDING INFORMATION

This research was funded by NordForsk. Grant/Award Number: 95263.

## Supporting information


Data S1.

Data S2.

Data S3.

Data S4.

## Data Availability

The data and materials necessary to reproduce the analyses and findings are publicly accessible via the following URL: https://www.cils4.eu/. The analytic code is not publicly accessible. The analyses presented here were not preregistered.

## Footnotes

^1^
We use “very low” for values between 1 and 1.5, “low” for values between 1.5 and 2.5, “medium” for values between 2.5 and 3.5, “high” for values between 3.5 and 4.5, and “very high” for values between 4.5 and 5.

## APPENDIX

**TABLE A1:** Percentages or means and standard deviations of measures.

	Total *N* = 4080	Non‐immigrant Christian *n* = 2086	Immigrant‐origin Christian *n* = 797	Immigrant‐origin Muslim *n* = 1197
Immigrant‐origin	48.4%	0%	100%	98.2%
Non‐immigrant	51.6%	100%	0%	1.8%
Girls	49.8%	50.4%	50.7%	48.1%
Boys	49.5%	49.0%	48.6%	50.8%
Missing gender	0.7%	0.5%	0.8%	1.1%
W1 age in years	15.24 (0.69)	15.14 (0.63)	15.30 (0.70)	15.39 (0.73)
W2 age in years	16.19 (0.68)	16.10 (0.64)	16.25 (0.69)	16.36 (0.74)
W3 age in years	17.36 (0.70)	17.29 (0.63)	17.44 (0.74)	17.45 (0.78)
W4 age in years	18.35 (0.65)	18.26 (0.59)	18.42 (0.66)	18.51 (0.72)
W5 age in years	19.41 (0.66)	19.33 (0.60)	19.45 (0.65)	19.57 (0.74)
W6 age in years	20.69 (0.72)	20.61 (0.69)	20.74 (0.69)	20.81 (0.76)
W7 age in years	22.49 (0.64)	22.42 (0.59)	22.56 (0.70)	22.62 (0.68)
Mother primary education	10%	2%	8.4%	25%
Mother secondary education	69.6%	81.3%	69.9%	49.2%
Mother tertiary education	12%	14.2%	16.2%	5.2%
Missing mother education	8.4%	2.5%	5.5%	20.6%
Father primary education	8.2%	2.5%	9.8%	17%
Father secondary education	65.7%	73.2%	62.5%	54.8%
Father tertiary education	17%	20.5%	17.9%	10.4%
Missing father education	9%	3.7%	9.8%	17.8%
Parental ISEI	45.04 (19.84)	50.62 (19.17)	43.61 (20.03)	35.10 (16.70)
W1 parent religiosity	2.83 (0.93)	2.49 (0.80)	2.86 (0.94)	3.54 (0.76)
W1 religiosity	2.55 (1.04)	2.13 (0.84)	2.46 (0.92)	3.34 (0.98)
W2 religiosity	2.53 (1.04)	2.14 (0.82)	2.40 (0.96)	3.35 (0.99)
W3 religiosity	2.47 (1.03)	2.08 (0.80)	2.41 (0.96)	3.34 (0.98)
W5 religiosity	2.31 (1.05)	1.94 (0.80)	2.18 (0.95)	3.26 (1.06)
W6 religiosity	2.32 (1.02)	2.00 (0.79)	2.18 (0.95)	3.19 (1.07)
W7 religiosity	2.13 (0.99)	1.83 (0.76)	2.03 (0.91)	3.02 (1.10)
W1 anxiety	2.36 (0.64)	2.39 (0.62)	2.41 (0.67)	2.30 (0.65)
W3 anxiety	2.36 (0.69)	2.37 (0.68)	2.41 (0.69)	2.28 (0.72)
W7 anxiety	2.60 (0.70)	2.61 (0.70)	2.63 (0.70)	2.56 (0.68)
W1 depression	1.95 (0.75)	1.98 (0.72)	2.01 (0.76)	1.86 (0.77)
W2 depression	1.92 (0.77)	1.96 (0.75)	1.93 (0.82)	1.76 (0.72)
W3 depression	1.84 (0.70)	1.87 (0.68)	1.85 (0.68)	1.76 (0.72)
W7 depression	2.02 (0.74)	2.05 (0.74)	2.00 (0.73)	1.93 (0.76)
W1 life satisfaction	7.51 (2.25)	7.57 (2.17)	7.41 (2.19)	7.46 (2.41)
W2 life satisfaction	7.63 (2.22)	7.67 (2.11)	7.62 (2.20)	7.55 (2.42)
W3 life satisfaction	7.92 (1.76)	7.93 (1.70)	7.90 (1.68)	7.93 (1.92)
W4 life satisfaction	7.30 (2.15)	7.36 (2.04)	7.10 (2.16)	7.30 (2.35)
W5 life satisfaction	7.50 (2.06)	7.58 (1.97)	7.36 (2.05)	7.42 (2.26)
W6 life satisfaction	7.77 (1.60)	7.87 (1.48)	7.62 (1.68)	7.65 (1.78)
W7 life satisfaction	7.38 (1.96)	7.37 (1.97)	7.37 (1.96)	7.45 (1.93)
W1 health	4.07 (0.89)	4.03 (0.89)	3.98 (0.90)	4.18 (0.87)
W2 health	4.00 (0.91)	3.92 (0.91)	3.96 (0.88)	4.22 (0.88)
W4 health	3.90 (0.93)	3.82 (0.91)	3.86 (0.95)	4.11 (0.92)
W6 health	3.71 (0.92)	3.63 (0.89)	3.77 (0.96)	3.84 (0.94)
W1 alcohol	1.98 (1.03)	2.26 (1.03)	2.14 (1.05)	1.39 (0.76)
W2 alcohol	2.23 (1.11)	2.63 (1.03)	2.35 (1.05)	1.44 (0.85)
W3 alcohol	2.41 (1.12)	2.85 (0.87)	2.48 (1.05)	1.43 (0.83)
W4 alcohol	2.45 (1.02)	2.81 (0.87)	2.53 (0.95)	1.60 (0.89)
W5 alcohol	2.35 (1.00)	2.69 (0.88)	2.48 (0.93)	1.48 (0.79)
W7 alcohol	2.46 (1.02)	2.75 (0.91)	2.57 (0.93)	1.57 (0.84)
W1 smoking	1.60 (1.29)	1.63 (1.32)	1.66 (1.33)	1.50 (1.20)
W2 smoking	1.76 (1.44)	1.81 (1.45)	1.93 (1.57)	1.58 (1.32)
W3 smoking	1.83 (1.52)	1.86 (1.54)	1.97 (1.61)	1.67 (1.42)
W4 smoking	2.06 (1.60)	2.09 (1.58)	2.24 (1.70)	1.86 (1.54)
W5 smoking	2.18 (1.66)	2.17 (1.64)	2.38 (1.74)	2.04 (1.63)
W7 smoking	2.26 (1.69)	2.20 (1.65)	2.46 (1.73)	2.24 (1.72)
W1 drugs	1.10 (0.49)	1.10 (0.46)	1.08 (0.41)	1.12 (0.57)
W2 drugs	1.12 (0.54)	1.12 (0.52)	1.18 (0.64)	1.09 (0.51)
W3 drugs	1.09 (0.46)	1.09 (0.43)	1.12 (0.51)	1.09 (0.49)
W4 drugs	1.20 (0.63)	1.23 (0.67)	1.22 (0.65)	1.12 (0.53)
W5 drugs	1.20 (0.62)	1.22 (0.63)	1.27 (0.74)	1.09 (0.47)
W7 drugs	1.30 (0.78)	1.33 (0.79)	1.39 (0.88)	1.15 (0.63)
W1 gender role values	2.26 (1.38)	2.46 (1.37)	2.38 (1.32)	1.82 (1.35)
W2 gender role values	2.76 (1.29)	2.96 (1.23)	2.85 (1.23)	2.29 (1.33)
W4 gender role values	3.03 (1.38)	3.20 (1.16)	3.12 (1.19)	2.58 (1.31)
W6 gender role values	3.45 (0.94)	3.61 (0.81)	3.44 (0.97)	3.10 (1.11)
W1 tolerance sexual liberties	2.62 (1.00)	3.06 (0.87)	2.61 (0.94)	1.86 (0.78)
W3 tolerance sexual liberties	3.14 (1.05)	3.57 (0.84)	3.11 (0.99)	2.22 (0.89)
W5 tolerance sexual liberties	3.38 (1.07)	3.83 (0.75)	3.42 (1.01)	2.31 (0.98)
W7 tolerance sexual liberties	3.68 (1.04)	4.05 (0.74)	3.73 (0.97)	2.61 (1.10)
W1 culture adoption	3.53 (1.13)		3.65 (1.04)	3.46 (1.18)
W2 culture adoption	3.66 (1.07)		3.70 (1.05)	3.64 (1.08)
W3 culture adoption	3.85 (0.88)		3.77 (0.89)	3.92 (0.87)
W5 culture adoption	3.82 (0.91)		3.93 (0.84)	3.73 (0.95)
W7 culture adoption	3.88 (0.86)		3.95 (0.80)	3.82 (0.91)
W1 culture maintenance	3.92 (0.98)		3.52 (1.00)	4.19 (0.87)
W2 culture maintenance	3.91 (0.96)		3.57 (0.96)	4.15 (0.88)
W3 culture maintenance	3.83 (0.86)		3.55 (0.86)	4.04 (0.80)
W5 culture maintenance	3.77 (0.99)		3.45 (1.03)	4.03 (0.87)
W7 culture maintenance	3.62 (1.03)		3.33 (1.00)	3.89 (0.96)
W1 German friends	3.09 (1.31)		3.49 (1.27)	2.83 (1.28)
W2 German friends	2.91 (1.27)		3.31 (1.26)	2.63 (1.20)
W3 German friends	3.01 (1.31)		3.35 (1.29)	2.74 (1.27)
W4 German friends	2.97 (1.27)		3.37 (1.26)	2.66 (1.19)
W5 German friends	3.11 (1.24)		3.55 (1.18)	2.76 (1.18)
W6 German friends	3.08 (1.21)		3.50 (1.14)	2.75 (1.15)
W7 German friends	3.21 (1.30)		3.83 (1.13)	2.65 (1.18)
W1 National identification	2.50 (0.99)		2.76 (1.00)	2.32 (0.94)
W2 National identification	2.68 (1.00)		2.97 (0.98)	2.48 (0.97)
W3 National identification	2.88 (0.93)		3.13 (0.89)	2.68 (0.90)
W4 National identification	2.72 (0.94)		2.97 (0.91)	2.52 (0.92)
W5 National identification	2.69 (0.93)		2.98 (0.90)	2.45 (0.89)
W6 National identification	3.07 (0.78)		3.27 (0.76)	2.92 (0.76)
W7 National identification	2.97 (0.84)		3.12 (0.83)	2.83 (0.83)

Abbreviation: W, wave of measurement.

## References

[cdev14151-bib-1001] Alba, R. , & Nee, V. (1997). Rethinking assimilation theory for a new era of immigration. International Migration Review, 31(4), 826–874. 12293207

[cdev14151-bib-0001] Asparouhov, T. , & Muthén, B. (2021). Auxiliary variables in mixture modeling: Using the BCH method in Mplus to estimate a distal outcome model and an arbitrary secondary model. Mplus web . Notes: No. 21, Version 11. https://www.statmodel.com/examples/webnotes/webnote21.pdf

[cdev14151-bib-0002] Balkaya, M. , Cheah, C. S. , & Tahseen, M. (2019). The mediating role of multiple group identities in the relations between religious discrimination and Muslim‐American adolescents' adjustment. Journal of Social Issues, 75(2), 538–567. 10.1111/josi.12326

[cdev14151-bib-0003] Bell, D. A. , Valenta, M. , & Strabac, Z. (2021). A comparative analysis of changes in anti‐immigrant and anti‐Muslim attitudes in Europe: 1990–2017. Comparative Migration Studies, 9(1), 1–24. 10.1186/s40878-021-00266-w

[cdev14151-bib-1005] Branscombe, N. R. , Schmitt, M. T. , & Harvey, R. D. (1999). Perceiving pervasive discrimination among African Americans: Implications for group identification and well‐being. Journal of Personality and Social Psychology, 77(1), 135–149. 10.1037/0022-3514.77.1.135

[cdev14151-bib-0004] Chan, M. , Tsai, K. M. , & Fuligni, A. J. (2015). Changes in religiosity across the transition to young adulthood. Journal of Youth and Adolescence, 44(8), 1555–1566. 10.1007/s10964-014-0157-0 25104418

[cdev14151-bib-0005] Connor, P. (2010). Contexts of immigrant receptivity and immigrant religious outcomes: The case of Muslims in Western Europe. Ethnic and Racial Studies, 33(3), 376–403. 10.1080/01419870902935963

[cdev14151-bib-0006] Curran, P. J. , Obeidat, K. , & Losardo, D. (2010). Twelve frequently asked questions about growth curve modeling. Journal of Cognition and Development, 11(2), 121–136. 10.1080/15248371003699969 21743795 PMC3131138

[cdev14151-bib-0007] Davis, R. F. , & Kiang, L. (2016). Religious identity, religious participation, and psychological well‐being in Asian American adolescents. Journal of Youth and Adolescence, 45(3), 532–546. 10.1007/s10964-015-0350-9 26346036

[cdev14151-bib-0008] de Ganzeboom, H. B. G. , Graaf, P. M. , & Treiman, D. J. (1992). A standard international socio‐economic index of occupational status. Social Science Research, 21, 1–56. 10.1016/0049-089X(92)90017-B

[cdev14151-bib-0009] Dimitrova, R. , & Aydinli‐Karakulak, A. (2016). Acculturation orientations mediate the link between religious identity and adjustment of Turkish‐Bulgarian and Turkish‐German adolescents. Springerplus, 5(1), 1–11. 10.1186/s40064-016-2688-1 27441143 PMC4938830

[cdev14151-bib-0010] Dyer, W. J. , Hardy, S. A. , & Goodman, M. (2022). Religiosity from age 12 to 20: Stability, change, and bidirectional effects of attendance, prayer, and salience. The International Journal for the Psychology of Religion, 32(3), 177–195. 10.1080/10508619.2020.1834745

[cdev14151-bib-0011] Enders, C. K. , & Bandalos, D. L. (2001). The relative performance of full information maximum likelihood estimation for missing data in structural equation models. Structural Equation Modeling, 8, 430–457. 10.1207/S15328007SEM0803_5

[cdev14151-bib-0012] Eskelinen, V. , & Verkuyten, M. (2020). Support for democracy and liberal sexual mores among Muslims in Western Europe. Journal of Ethnic and Migration Studies, 46(11), 2346–2366. 10.1080/1369183X.2018.1521715

[cdev14151-bib-0013] Etengoff, C. , & Daiute, C. (2013). Sunni‐Muslim American religious development during emerging adulthood. Journal of Adolescent Research, 28(6), 690–714. 10.1177/0743558413477197

[cdev14151-bib-0014] Fleischmann, F. (2022). Researching religion and migration 20 years after ‘9/11’: Taking stock and looking ahead. Zeitschrift für Religion, Gesellschaft Und Politik, 1‐26, 347–372. 10.1007/s41682-022-00103-6 PMC8889873 35252743

[cdev14151-bib-0015] Foner, N. , & Alba, R. (2008). Immigrant religion in the US and Western Europe: Bridge or barrier to inclusion? International Migration Review, 42(2), 360–392. https://www.jstor.org/stable/27645255

[cdev14151-bib-0016] French, D. C. , Christ, S. , Lu, T. , & Purwono, U. (2014). Trajectories of Indonesian adolescents' religiosity, problem behavior, and friends' religiosity: Covariation and sequences. Child Development, 85(4), 1634–1646. 10.1111/cdev.12234 24673260

[cdev14151-bib-0017] Friedman, M. , & Saroglou, V. (2010). Religiosity, psychological acculturation to the host culture, self‐esteem and depressive symptoms among stigmatized and nonstigmatized religious immigrant groups in Western Europe. Basic and Applied Social Psychology, 32(2), 185–195. 10.1080/01973531003738387

[cdev14151-bib-0018] Ghandour, L. A. , Karam, E. G. , & Maalouf, W. E. (2009). Lifetime alcohol use, abuse and dependence among university students in Lebanon: Exploring the role of religiosity in different religious faiths. Addiction, 104(6), 940–948. 10.1111/j.1360-0443.2009.02575.x 19466919

[cdev14151-bib-0019] Goforth, A. N. , Oka, E. R. , Leong, F. T. , & Denis, D. J. (2014). Acculturation, acculturative stress, religiosity and psychological adjustment among Muslim Arab American adolescents. Journal of Muslim Mental Health, 8(2), 3–19. 10.3998/jmmh.10381607.0008.202

[cdev14151-bib-0020] Goodman, M. A. , & Dyer, W. J. (2020). From parent to child: Family factors that influence faith transmission. Psychology of Religion and Spirituality, 12(2), 178–190. 10.1037/rel0000283

[cdev14151-bib-0021] Güngör, D. , Bornstein, M. H. , & Phalet, K. (2012). Religiosity, values, and acculturation: A study of Turkish, Turkish‐Belgian, and Belgian adolescents. International Journal of Behavioral Development, 36(5), 367–373. 10.1177/0165025412448357 23155300 PMC3496254

[cdev14151-bib-0022] Hardy, S. A. , Nelson, J. M. , Moore, J. P. , & King, P. E. (2019). Processes of religious and spiritual influence in adolescence: A systematic review of 30 years of research. Journal of Research on Adolescence, 29(2), 254–275. 10.1111/jora.12486 31206875

[cdev14151-bib-0023] Hardy, S. A. , Pratt, M. W. , Pancer, S. M. , Olsen, J. A. , & Lawford, H. L. (2011). Community and religious involvement as contexts of identity change across late adolescence and emerging adulthood. International Journal of Behavioral Development, 35(2), 125–135. 10.1177/0165025410375920

[cdev14151-bib-0024] Hodapp, B. , & Zwingmann, C. (2019). Religiosity/spirituality and mental health: A meta‐analysis of studies from the German‐speaking area. Journal of Religion and Health, 58(6), 1970–1998. 10.1007/s10943-019-00759-0 30632002

[cdev14151-bib-1006] Hogg, M. A. (2007). Uncertainty‐identity theory. In M. P. Zanna (Ed.), Advances in experimental social psychology (Vol. 39, pp. 69–126). Elsevier Academic Press. 10.1016/S0065-2601(06)39002-8

[cdev14151-bib-0025] Jacob, K. , & Kalter, F. (2013). Intergenerational change in religious salience among immigrant families in four European countries. International Migration, 51(3), 38–56. 10.1111/imig.12108

[cdev14151-bib-0026] Jung, T. , & Wickrama, K. A. (2008). An introduction to latent class growth analysis and growth mixture modeling. Social and Personality Psychology Compass, 2(1), 302–317. 10.1111/j.1751-9004.2007.00054.x

[cdev14151-bib-0027] Kalter, F. , Heath, A. F. , Hewstone, M. , Jonsson, J. O. , Kalmijn, M. , Kogan, I. , & van Tubergen, F. (2016). Children of Immigrants Longitudinal Survey in Four European Countries (CILS4EU)—Full version. Data file for on‐site use. GESIS Data Archive, Cologne, ZA5353 Data file Version 1.2.0 . 10.4232/cils4eu.5353.3.3.0

[cdev14151-bib-0028] Kalter, F. , Kogan, I. , & Dollmann, J. (2021). Children of Immigrants Longitudinal Survey in Four European Countries—Germany (CILS4EU‐DE)—Full version. Data file for on‐site use. GESIS Data Archive, Cologne, ZA6655 Data file Version 6.0.0 . 10.4232/cils4eu-de.6655.6.0.0

[cdev14151-bib-0029] King, P. E. , & Boyatzis, C. J. (2015). Religious and spiritual development. In M. E. Lamb & R. M. Lerner (Eds.), Handbook of child psychology and developmental science: Socioemotional processes (pp. 975–1021). John Wiley & Sons, Inc. 10.1002/9781118963418.childpsy323

[cdev14151-bib-0030] King, P. E. , Hardy, S. A. , & Noe, S. (2021). Developmental perspectives on adolescent religious and spiritual development. Adolescent Research Review, 6(3), 253–264. 10.1007/s40894-021-00159-0 PMC8188949 34127947

[cdev14151-bib-0031] Koenig, L. B. , McGue, M. , & Iacono, W. G. (2008). Stability and change in religiousness during emerging adulthood. Developmental Psychology, 44(2), 532–543. 10.1037/0012-1649.44.2.532 18331142

[cdev14151-bib-0032] Kretschmer, D. (2018). Explaining differences in gender role attitudes among migrant and native adolescents in Germany: Intergenerational transmission, religiosity, and integration. Journal of Ethnic and Migration Studies, 44(13), 2197–2218. 10.1080/1369183X.2017.1388159

[cdev14151-bib-0033] Lee, D. B. , & Neblett, E. W. (2019). Religious development in African American adolescents: Growth patterns that offer protection. Child Development, 90(1), 245–259. 10.1111/cdev.12896 28708241 PMC5767549

[cdev14151-bib-0034] Lewis, V. A. , & Kashyap, R. (2013). Are Muslims a distinctive minority? An empirical analysis of religiosity, social attitudes, and Islam. Journal for the Scientific Study of Religion, 52(3), 617–626. 10.1111/jssr.12044

[cdev14151-bib-0035] Loewenthal, K. M. , MacLeod, A. K. , & Cinnirella, M. (2002). Are women more religious than men? Gender differences in religious activity among different religious groups in the UK. Personality and Individual Differences, 32(1), 133–139. 10.1016/S0191-8869(01)00011-3

[cdev14151-bib-0036] Lopez, A. B. , Huynh, V. W. , & Fuligni, A. J. (2011). A longitudinal study of religious identity and participation during adolescence. Child Development, 82(4), 1297–1309. 10.1111/j.1467-8624.2011.01609.x 21679174

[cdev14151-bib-0037] Maes, M. , Stevens, G. W. , & Verkuyten, M. (2014). Perceived ethnic discrimination and problem behaviors in Muslim immigrant early adolescents: Moderating effects of ethnic, religious, and national group identification. The Journal of Early Adolescence, 34(7), 940–966. 10.1177/0272431613514629

[cdev14151-bib-0038] McNamara Barry, C. , Nelson, L. , Davarya, S. , & Urry, S. (2010). Religiosity and spirituality during the transition to adulthood. International Journal of Behavioral Development, 34(4), 311–324. 10.1177/0165025409350964

[cdev14151-bib-0039] Molteni, F. , & van Tubergen, F. (2022). Immigrant generation and religiosity: A study of Christian immigrant groups in 33 European countries. European Societies, 24(5), 605–627. 10.1080/14616696.2022.2044067

[cdev14151-bib-0040] Muthén, B. , & Muthén, L. K. (2000). Integrating person‐centered and variable‐centered analyses: Growth mixture modeling with latent trajectory classes. Alcoholism: Clinical and Experimental Research, 24(6), 882–891. 10888079

[cdev14151-bib-0041] Muthén, L. K. , & Muthén, B. O. (1998‐2017). Mplus User's Guide (Eighth ed.). Muthén & Muthén.

[cdev14151-bib-1003] Nagin, D. S. (1999). Analyzing developmental trajectories: A semiparametric, group‐based approach. Psychological Methods, 4(2), 139–157. 10.1037/1082-989X.4.2.139 11285809

[cdev14151-bib-0042] Oberoi, A. K. , & Trickett, E. J. (2018). Religion in the hallways: Academic performance and psychological distress among immigrant origin Muslim adolescents in high schools. American Journal of Community Psychology, 61(3–4), 344–357. 10.1002/ajcp.12238 29578586

[cdev14151-bib-0043] Peek, L. (2005). Becoming Muslim: The development of a religious identity. Sociology of Religion, 66(3), 215–242. 10.2307/4153097

[cdev14151-bib-0044] Petts, R. J. (2009). Trajectories of religious participation from adolescence to young adulthood. Journal for the Scientific Study of Religion, 48(3), 552–571. 10.1111/j.1468-5906.2009.01465.x

[cdev14151-bib-0045] PEW . (2017). 5 facts about the Muslim population in Europe . https://www.pewresearch.org/fact‐tank/2017/11/29/5‐facts‐about‐the‐muslim‐population‐in‐europe/

[cdev14151-bib-0046] Phalet, K. , Fleischmann, F. , & Hillekens, J. (2018). Religious identity and acculturation of immigrant minority youth. European Psychologist, 23(1), 32–43. 10.1027/1016-9040/a000309

[cdev14151-bib-1007] Portes, A. , & Rumbaut, R. G. (2001). Legacies: The story of the immigrant second generation. University of California Press.

[cdev14151-bib-0047] Schnabel, L. (2018). More religious, less dogmatic: Toward a general framework for gender differences in religion. Social Science Research, 75, 58–72. 10.1016/j.ssresearch.2018.06.010 30080492

[cdev14151-bib-0048] Schnitker, S. A. , Medenwaldt, J. M. , & Williams, E. G. (2021). Religiosity in adolescence. Current Opinion in Psychology, 40, 155–159. 10.1016/j.copsyc.2020.09.012 33176270

[cdev14151-bib-0049] Simsek, M. , Fleischmann, F. , & van Tubergen, F. (2019). Similar or divergent paths? Religious development of Christian and Muslim adolescents in Western Europe. Social Science Research, 79, 160–180. 10.1016/j.ssresearch.2018.09.004 30857660

[cdev14151-bib-0050] Smith, C. , Denton, M. L. , Faris, R. , & Regnerus, M. (2002). Mapping American adolescent religious participation. Journal for the Scientific Study of Religion, 41(4), 597–612. 10.1111/1468-5906.00148

[cdev14151-bib-0051] Spiegler, O. , Wölfer, R. , & Hewstone, M. (2019). Dual identity development and adjustment in Muslim minority adolescents. Journal of Youth and Adolescence, 48, 1924–1937. 10.1007/s10964-019-01117-9 31520235 PMC6813286

[cdev14151-bib-1002] Sterba, S. K. , & Bauer, D. J. (2010). Matching method with theory in person‐oriented developmental psychopathology research. Development and Psychopathology, 22(2), 239–254. 10.1017/S0954579410000015 20423538

[cdev14151-bib-0052] Stoppa, T. M. , & Lefkowitz, E. S. (2010). Longitudinal changes in religiosity among emerging adult college students. Journal of Research on Adolescence, 20(1), 23–38. 10.1111/j.1532-7795.2009.00630.x 20209118 PMC2830663

[cdev14151-bib-0053] Stuart, J. , & Ward, C. (2018). The relationships between religiosity, stress, and mental health for Muslim immigrant youth. Mental Health, Religion and Culture, 21(3), 246–261. 10.1080/13674676.2018.1462781

[cdev14151-bib-1004] Tajfel, H. , & Turner, J. C. (1979). An integrative theory of intergroup conflict. In W. G. Austin & S. Worchel (Eds.), The social psychology of intergroup relations (pp. 33–47). Brooks/Cole.

[cdev14151-bib-0054] Umaña‐Taylor, A. J. , Quintana, S. M. , Lee, R. M. , Cross, W. E., Jr. , Rivas‐Drake, D. , Schwartz, S. J. , Syed, M. , Yip, T. , Seaton, E. , & Ethnic and Racial Identity in the 21st Century Study Group . (2014). Ethnic and racial identity during adolescence and into young adulthood: An integrated conceptualization. Child Development, 85(1), 21–39. 24490890 10.1111/cdev.12196 PMC6673642

[cdev14151-bib-0055] Verkuyten, M. (2018). Religious fundamentalism and radicalization among Muslim minority youth in Europe. European Psychologist, 23(1), 21–31. 10.1027/1016-9040/a000314

[cdev14151-bib-0056] Wright, A. W. , Yendork, J. S. , & Kliewer, W. (2018). Patterns of spiritual connectedness during adolescence: Links to coping and adjustment in low‐income urban youth. Journal of Youth and Adolescence, 47(12), 2608–2624. 10.1007/s10964-018-0886-6 29951730 PMC6246777

[cdev14151-bib-0057] Yonker, J. E. , Schnabelrauch, C. A. , & DeHaan, L. G. (2012). The relationship between spirituality and religiosity on psychological outcomes in adolescents and emerging adults: A meta‐analytic review. Journal of Adolescence, 35(2), 299–314. 10.1016/j.adolescence.2011.08.010 21920596

