# Supplementary material for: Religious development from adolescence to early adulthood among Muslim and Christian youth in Germany: A person‐oriented approach
Source: Child Dev. 2024 Aug 27;96(1):141–60. doi: 10.1111/cdev.14151 (PMC11693839; doi:10.1111/cdev.14151)
Supplement: Supplementary file 1 — Data S1. [file CDEV-96-141-s004.docx]

**Online Supplementary Materials (OSM)**

**OSM A – Descriptive statistics**

Table A1. Correlations of main variables among non-immigrant Christians.

|  | Religiosity | | | | | |
| --- | --- | --- | --- | --- | --- | --- |
|  | W1 | W2 | W3 | W5 | W6 | W7 |
| Gender (0=boys, 1=girls) | .097*** | .093*** | .081** | .102*** | .126*** | .115*** |
| W1 Age in years | -.158*** | -.109*** | -.094*** | -.057* | -.123*** | -.061 |
| W2 Age in years | -.168*** | -.114*** | -.119*** | -.077* | -.143*** | -.072* |
| W3 Age in years | -.146*** | -.115*** | -.113*** | -.060* | -.121*** | -.067* |
| W4 Age in years | -.133*** | -.091*** | -.094*** | -.070* | -.127*** | -.065 |
| W5 Age in years | -.133*** | -.099*** | -.109*** | -.079** | -.139*** | -.084* |
| W6 Age in years | -.181*** | -.139*** | -.142*** | -.133*** | -.161*** | -.083* |
| W7 Age in years | -.147*** | -.097** | -.130*** | -.106** | -.120*** | -.070* |
| Mother primary education | -.025 | -.053* | -.039 | -.014 | -.008 | -.076* |
| Mother secondary education | -.067** | -.038 | -.055* | -.067* | -.051 | -.023 |
| Mother tertiary education | .080*** | .057* | .066* | .071* | .053 | .039 |
| Father primary education | -.046* | -.039 | -.020 | -.002 | -.004 | -.001 |
| Father secondary education | -.062** | -.063** | -.065* | -.065* | -.040 | -.030 |
| Father tertiary education | .082*** | .077** | .072** | .067* | .042 | .030 |
| Parental ISEI | .130*** | .095*** | .083** | .060* | .088** | .065* |
| W1 Parent religiosity | .335*** | .321*** | .319*** | .335*** | .350*** | .338*** |
| W1 Anxiety | .076*** | .097*** | .095*** | .102*** | .066* | .092** |
| W1 Anxiety | .073** | .077** | .112*** | .084** | .104*** | .114*** |
| W3 Anxiety | .035 | .039 | .054 | .046 | .047 | .042 |
| W1 Depression | -.001 | .017 | .007 | .008 | .023 | .018 |
| W2 Depression | .027 | .043 | .058* | .064* | .031 | .005 |
| W3 Depression | .050 | .050 | .057* | .012 | .054 | .052 |
| W7 Depression | .038 | .025 | .038 | .018 | .019 | .013 |
| W1 Life satisfaction | .053* | .038 | .064* | .064* | .091** | .057 |
| W2 Life satisfaction | .023 | .045 | .064* | .035 | .057 | .072* |
| W3 Life satisfaction | .053* | .045 | .084*** | .080** | .113*** | .080* |
| W4 Life satisfaction | .002 | -.002 | .030 | .035 | .050 | .017 |
| W5 Life satisfaction | .031 | -.007 | -.005 | .056* | .063* | .021 |
| W6 Life satisfaction | .054 | .043 | .043 | .103*** | .122*** | .089** |
| W7 Life satisfaction | .007 | .034 | .055 | .057 | .058 | .048 |
| W1 Health | .063** | .037 | .057* | .051 | .055 | .035 |
| W2 Health | .082** | .075** | .108*** | .071* | .105** | .063 |
| W4 Health | .090*** | .065* | .093*** | .097*** | .090** | .077* |
| W6 Health | .008 | .011 | .017 | .030 | .056 | -.028 |
| W1 Alcohol | -.145*** | -.117*** | -.109*** | -.112*** | -.039 | -.072 |
| W2 Alcohol | -.079** | -.060* | -.078** | -.067* | -.051 | -.087* |
| W3 Alcohol | -.024 | -.045 | -.068** | -.036 | -.042 | -.052 |
| W4 Alcohol | -.004 | -.017 | .009 | .023 | .026 | -.002 |
| W5 Alcohol | .032 | .037 | .064* | .048 | .067* | -.004 |
| W7 Alcohol | .074* | .059 | .078* | .032 | .034 | -.009 |
| W1 Smoking | -.158*** | -.122*** | -.125*** | -.098** | -.091* | -.102* |
| W2 Smoking | -.151*** | -.104*** | -.101*** | -.068* | -.089** | -.081* |
| W3 Smoking | -.124*** | -.120*** | -.141*** | -.094*** | -.080* | -.073* |
| W4 Smoking | -.110*** | -.097*** | -.101*** | -.090** | -.067* | -.041 |
| W5 Smoking | -.136*** | -.117*** | -.120*** | -.120*** | -.100** | -.096** |
| W7 Smoking | -.094** | -.076* | -.120*** | -.108** | -.090** | -.098** |
| W1 Drugs | -.064* | -.038 | -.014 | -.107** | -.060 | -.072 |
| W2 Drugs | -.035 | .025 | .026 | .014 | -.025 | .010 |
| W3 Drugs | -.018 | -.019 | -.018 | .000 | -.015 | .043 |
| W4 Drugs | -.056* | -.071* | -.039 | -.031 | -.065* | -.075* |
| W5 Drugs | -.089** | -.077** | -.091** | -.078** | -.090** | -.092** |
| W7 Drugs | -.145*** | -.124*** | -.117*** | -.171*** | -.124*** | -.180*** |
| W1 Gender role values | .000 | -.013 | -.025 | -.046 | -.054 | -.048 |
| W2 Gender role values | -.043 | -.058* | -.067* | -.072* | -.083** | -.050 |
| W4 Gender role values | -.049 | -.043 | -.066* | -.076** | -.083** | -.071* |
| W6 Gender role values | -.056 | -.058 | -.079* | -.049 | -.049 | -.027 |
| W1 Tolerance sexual liberties | -.085*** | -.054* | -.096*** | -.081** | -.104*** | -.134*** |
| W3 Tolerance sexual liberties | -.099*** | -.103*** | -.141*** | -.147*** | -.175*** | -.188*** |
| W5 Tolerance sexual liberties | -.125*** | -.112*** | -.130*** | -.161*** | -.192*** | -.217*** |
| W7 Tolerance sexual liberties | -.131*** | -.146*** | -.146*** | -.203*** | -.195*** | -.247*** |

*Note.* W = wave of measurement.

Table A2. Correlations of main variables among immigrant-origin Christians.

|  | Religiosity | | | | | |
| --- | --- | --- | --- | --- | --- | --- |
|  | W1 | W2 | W3 | W5 | W6 | W7 |
| Gender (0=boys, 1=girls) | -.012 | .024 | .024 | .049 | .069 | .101 |
| W1 Age in years | .016 | .034 | .024 | .073 | .070 | .048 |
| W2 Age in years | .018 | .034 | .014 | .037 | .040 | .023 |
| W3 Age in years | -.021 | .024 | -.025 | .070 | .052 | .030 |
| W4 Age in years | .001 | .045 | .052 | .083 | .069 | .043 |
| W5 Age in years | .015 | .049 | .050 | .090 | .116* | .089 |
| W6 Age in years | -.007 | .028 | .069 | .085 | .089 | .098 |
| W7 Age in years | .038 | .086 | .106 | .049 | .052 | .058 |
| Mother primary education | .029 | .029 | .013 | .052 | -.016 | .012 |
| Mother secondary education | -.030 | -.019 | .004 | -.051 | -.022 | -.117* |
| Mother tertiary education | .014 | .003 | -.013 | .025 | .032 | .119* |
| Father primary education | -.011 | .011 | -.023 | -.017 | -.031 | -.065 |
| Father secondary education | -.012 | -.046 | .049 | .035 | .009 | -.072 |
| Father tertiary education | .022 | .044 | -.040 | -.028 | .008 | .116 |
| Parental ISEI | -.050 | -.060 | -.052 | -.050 | -.031 | .012 |
| W1 Parent religiosity | .442*** | .407*** | .373*** | .344*** | .302*** | .285*** |
| W1 Anxiety | -.023 | -.023 | -.059 | -.066 | -.026 | -.069 |
| W1 Anxiety | -.106* | -.082 | -.079 | -.079 | -.032 | -.106 |
| W3 Anxiety | .020 | .001 | -.032 | .032 | .032 | .020 |
| W1 Depression | -.013 | -.049 | -.057 | -.058 | .010 | -.047 |
| W2 Depression | -.042 | -.081 | -.118* | -.027 | -.027 | -.021 |
| W3 Depression | -.076 | -.109* | -.096* | -.078 | -.036 | -.085 |
| W7 Depression | .022 | -.012 | -.053 | -.044 | .015 | -.091 |
| W1 Life satisfaction | .057 | .039 | .056 | .024 | .026 | .068 |
| W2 Life satisfaction | .085* | .116** | .130** | .136** | .149** | .109 |
| W3 Life satisfaction | .150*** | .134** | .164*** | .138** | .085 | .088 |
| W4 Life satisfaction | .049 | .045 | .042 | .029 | -.072 | -.056 |
| W5 Life satisfaction | .012 | .004 | .053 | .132** | .050 | .103 |
| W6 Life satisfaction | .031 | -.015 | -.027 | -.004 | .015 | .009 |
| W7 Life satisfaction | -.038 | -.040 | .010 | -.014 | -.025 | .076 |
| W1 Health | .089* | .121** | .065 | .085 | .094 | .112 |
| W2 Health | .039 | .039 | .050 | .002 | -.035 | -.002 |
| W4 Health | .054 | .052 | .081 | .120* | .079 | .065 |
| W6 Health | -.049 | -.038 | .030 | -.079 | .014 | .026 |
| W1 Alcohol | -.131** | -.075 | -.060 | -.019 | -.099 | -.019 |
| W2 Alcohol | -.203*** | -.194*** | -.156*** | -.133** | -.173** | -.093 |
| W3 Alcohol | -.134** | -.140** | -.161*** | -.126* | -.143** | -.103 |
| W4 Alcohol | -.139** | -.143** | -.176*** | -.138** | -.158** | -.117* |
| W5 Alcohol | -.075 | -.075 | -.129** | -.158*** | -.183*** | -.120* |
| W7 Alcohol | -.049 | -.085 | -.129* | -.128* | -.130* | -.148* |
| W1 Smoking | -.038 | .012 | .011 | -.002 | -.042 | -.005 |
| W2 Smoking | -.082* | -.057 | -.036 | -.048 | -.086 | .024 |
| W3 Smoking | -.098* | -.037 | -.088* | -.089 | -.141* | -.070 |
| W4 Smoking | -.125** | -.024 | -.066 | -.111* | -.190*** | -.103 |
| W5 Smoking | -.074 | -.017 | -.056 | -.087 | -.166** | -.057 |
| W7 Smoking | -.105 | -.004 | -.011 | -.069 | -.155** | -.107 |
| W1 Drugs | -.030 | .016 | .035 | -.041 | -.057 | .018 |
| W2 Drugs | -.094* | -.080* | -.128** | -.091 | -.105 | -.011 |
| W3 Drugs | -.023 | -.066 | -.103* | -.040 | -.104 | -.059 |
| W4 Drugs | -.050 | -.061 | -.071 | -.099* | -.172** | -.141** |
| W5 Drugs | -.022 | -.075 | -.013 | -.136** | -.143* | -.100 |
| W7 Drugs | -.141* | -.180** | -.171** | -.198*** | -.208*** | -.198*** |
| W1 Gender role values | -.049 | -.068 | -.070 | -.108* | -.095 | -.015 |
| W2 Gender role values | -.048 | -.063 | -.084 | -.061 | -.084 | .040 |
| W4 Gender role values | -.045 | -.111* | -.138** | -.092 | -.152** | -.087 |
| W6 Gender role values | -.033 | -.038 | -.127* | -.049 | -.156** | -.075 |
| W1 Tolerance sexual liberties | -.274*** | -.266*** | -.297*** | -.300*** | -.301*** | -.254*** |
| W3 Tolerance sexual liberties | -.273*** | -.330*** | -.362*** | -.329*** | -.400*** | -.306*** |
| W5 Tolerance sexual liberties | -.235*** | -.261*** | -.362*** | -.367*** | -.428*** | -.344*** |
| W7 Tolerance sexual liberties | -.166** | -.236*** | -.321*** | -.317*** | -.384*** | -.416*** |
| W1 Culture adoption | -.015 | -.054 | -.039 | -.031 | .002 | -.053 |
| W2 Culture adoption | -.007 | .014 | .047 | .042 | .027 | -.029 |
| W3 Culture adoption | .037 | .095* | .064 | .086 | .037 | .030 |
| W5 Culture adoption | -.034 | .029 | -.011 | .009 | .002 | -.048 |
| W7 Culture adoption | -.007 | .028 | .094 | .134* | .129* | .113 |
| W1 Heritage culture maintenance | .221*** | .192*** | .245*** | .213*** | .224*** | .187*** |
| W2 Heritage culture maintenance | .150*** | .197*** | .152*** | .143** | .145** | .132* |
| W3 Heritage culture maintenance | .124** | .196*** | .176*** | .115* | .051 | .088 |
| W5 Heritage culture maintenance | .103* | .092 | .120* | .111* | .110* | .098 |
| W7 Heritage culture maintenance | .113 | .155** | .161** | .136* | .156** | .185** |
| W1 German friends | -.029 | -.059 | -.094* | -.070 | -.033 | -.059 |
| W2 German friends | -.065 | -.137*** | -.155*** | -.155*** | -.118* | -.135* |
| W3 German friends | -.049 | -.105* | -.158*** | -.122* | -.101 | -.139* |
| W4 German friends | -.061 | -.094* | -.132** | -.133** | -.094 | -.128* |
| W5 German friends | -.055 | -.062 | -.131** | -.099* | -.145** | -.140* |
| W6 German friends | -.047 | -.083 | -.144** | -.145** | -.150** | -.165** |
| W7 German friends | -.078 | -.070 | -.168** | -.137* | -.128* | -.121* |
| W1 National identification | -.152*** | -.165*** | -.210*** | -.228*** | -.145** | -.129* |
| W2 National identification | -.097* | -.130*** | -.152*** | -.123* | -.112* | -.146* |
| W3 National identification | -.166*** | -.193*** | -.214*** | -.164*** | -.174** | -.151* |
| W4 National identification | -.111* | -.136** | -.165*** | -.142** | -.196*** | -.154** |
| W5 National identification | -.158** | -.157** | -.204*** | -.191*** | -.248*** | -.230*** |
| W6 National identification | -.091 | -.124* | -.139* | -.144** | -.153** | -.120* |
| W7 National identification | -.064 | -.075 | -.096 | -.095 | -.105 | -.068 |

*Note.* W = wave of measurement.

Table A3. Correlations of main variables among Muslims.

|  | Religiosity | | | | | |
| --- | --- | --- | --- | --- | --- | --- |
|  | W1 | W2 | W3 | W5 | W6 | W7 |
| Gender (0=boys, 1=girls) | -.112*** | -.084* | -.070 | -.091* | -.019 | .025 |
| W1 Age in years | .019 | .008 | .009 | -.003 | -.047 | -.098 |
| W2 Age in years | .009 | -.008 | -.058 | -.045 | -.024 | -.037 |
| W3 Age in years | -.018 | -.049 | -.037 | -.073 | -.073 | -.109 |
| W4 Age in years | .022 | .000 | .006 | -.010 | -.048 | -.081 |
| W5 Age in years | -.001 | -.051 | -.016 | -.017 | -.079 | -.094 |
| W6 Age in years | .008 | -.065 | -.042 | -.054 | -.113* | -.123* |
| W7 Age in years | -.010 | -.025 | -.024 | -.075 | -.118* | -.120* |
| Mother primary education | .085* | .151*** | .050 | .044 | .008 | .088 |
| Mother secondary education | -.052 | -.113** | -.052 | -.041 | -.016 | -.066 |
| Mother tertiary education | -.060 | -.062 | .009 | -.001 | .017 | -.031 |
| Father primary education | -.014 | .039 | -.037 | -.021 | -.117* | -.044 |
| Father secondary education | .039 | -.005 | .051 | .061 | .107* | .087 |
| Father tertiary education | -.038 | -.039 | -.028 | -.061 | -.012 | -.072 |
| Parental ISEI | -.029 | -.013 | -.021 | -.026 | -.013 | -.077 |
| W1 Parent religiosity | .404*** | .409*** | .380*** | .351*** | .356*** | .414*** |
| W1 Anxiety | -.030 | -.004 | -.045 | .014 | .032 | .086 |
| W1 Anxiety | -.131*** | -.145*** | -.209*** | -.153*** | -.123* | -.133* |
| W3 Anxiety | -.068 | -.102 | -.180** | -.058 | -.100 | -.079 |
| W1 Depression | -.080** | -.076* | -.087* | -.054 | -.020 | .039 |
| W2 Depression | -.081* | -.076* | -.077 | -.008 | -.113* | -.038 |
| W3 Depression | -.087* | -.113** | -.126*** | -.090* | -.085 | -.128* |
| W7 Depression | -.101 | -.111* | -.151** | -.084 | -.174** | -.134* |
| W1 Life satisfaction | .075* | .050 | .039 | .065 | .035 | .001 |
| W2 Life satisfaction | .103** | .086** | .108** | .087* | .148** | .071 |
| W3 Life satisfaction | .071 | .058 | .150*** | .084 | .107* | .070 |
| W4 Life satisfaction | .063 | .037 | .078 | .075 | .082 | .112* |
| W5 Life satisfaction | .121** | .084 | .136** | .127** | .143** | .135* |
| W6 Life satisfaction | -.022 | .012 | .072 | .010 | .087 | .041 |
| W7 Life satisfaction | .053 | .053 | .110* | .013 | .028 | .078 |
| W1 Health | .052 | .049 | .020 | .019 | -.006 | .024 |
| W2 Health | .084* | .087* | .019 | .020 | -.001 | .052 |
| W4 Health | .094* | .094* | .090* | .111* | .111* | .181** |
| W6 Health | .090 | .087 | .077 | .124* | .099 | .164* |
| W1 Alcohol | -.199*** | -.190*** | -.171*** | -.134** | -.168** | -.202** |
| W2 Alcohol | -.238*** | -.242*** | -.161*** | -.192*** | -.280*** | -.246*** |
| W3 Alcohol | -.270*** | -.241*** | -.262*** | -.258*** | -.315*** | -.330*** |
| W4 Alcohol | -.273*** | -.229*** | -.239*** | -.239*** | -.291*** | -.359*** |
| W5 Alcohol | -.335*** | -.325*** | -.333*** | -.366*** | -.406*** | -.384*** |
| W7 Alcohol | -.253*** | -.254*** | -.311*** | -.250*** | -.387*** | -.431*** |
| W1 Smoking | -.042 | -.052 | -.008 | -.047 | -.015 | -.019 |
| W2 Smoking | -.094** | -.088** | -.040 | -.050 | -.103** | -.040 |
| W3 Smoking | -.093* | -.088* | -.063 | -.047 | -.113* | -.122* |
| W4 Smoking | -.058 | -.076 | -.033 | -.067 | -.103 | -.126* |
| W5 Smoking | -.048 | -.114** | -.050 | -.068 | -.135** | -.128* |
| W7 Smoking | -.062 | -.068 | -.017 | -.006 | -.099 | -.102 |
| W1 Drugs | -.011 | -.023 | -.023 | .013 | -.015 | .009 |
| W2 Drugs | -.094** | -.069* | -.033 | -.071 | -.123* | -.104 |
| W3 Drugs | -.100** | -.104** | -.030 | -.053 | -.027 | -.106 |
| W4 Drugs | -.105* | -.069 | -.006 | -.120** | -.107* | -.151** |
| W5 Drugs | -.085* | -.055 | -.033 | -.045 | -.039 | -.193*** |
| W7 Drugs | -.089 | -.044 | -.059 | -.044 | -.031 | -.098 |
| W1 Gender role values | -.173*** | -.191*** | -.201*** | -.170*** | -.151** | -.165** |
| W2 Gender role values | -.195*** | -.215*** | -.192*** | -.124** | -.100* | -.183*** |
| W4 Gender role values | -.104* | -.169*** | -.178*** | -.182*** | -.080 | -.149** |
| W6 Gender role values | -.113* | -.118* | -.085 | -.077 | -.106* | -.201*** |
| W1 Tolerance sexual liberties | -.329*** | -.284*** | -.289*** | -.300*** | -.295*** | -.334*** |
| W3 Tolerance sexual liberties | -.328*** | -.334*** | -.362*** | -.323*** | -.293*** | -.325*** |
| W5 Tolerance sexual liberties | -.373*** | -.371*** | -.377*** | -.444*** | -.377*** | -.457*** |
| W7 Tolerance sexual liberties | -.411*** | -.431*** | -.431*** | -.447*** | -.441*** | -.492*** |
| W1 Culture adoption | -.064* | -.064 | -.059 | .002 | -.017 | -.010 |
| W2 Culture adoption | -.114*** | -.151*** | -.158*** | -.119** | -.119* | -.093 |
| W3 Culture adoption | -.071 | -.102** | -.117** | -.133** | -.151** | -.134* |
| W5 Culture adoption | -.171*** | -.156*** | -.100* | -.140*** | -.128* | -.178** |
| W7 Culture adoption | -.118* | -.161** | -.130* | -.195*** | -.193*** | -.185*** |
| W1 Heritage culture maintenance | .183*** | .171*** | .192*** | .157*** | .158*** | .224*** |
| W2 Heritage culture maintenance | .177*** | .211*** | .243*** | .265*** | .227*** | .296*** |
| W3 Heritage culture maintenance | .178*** | .164*** | .249*** | .169*** | .194*** | .244*** |
| W5 Heritage culture maintenance | .174*** | .225*** | .212*** | .233*** | .237*** | .278*** |
| W7 Heritage culture maintenance | .264*** | .200*** | .196*** | .210*** | .229*** | .309*** |
| W1 German friends | -.059 | -.086* | -.060 | -.078 | -.001 | -.046 |
| W2 German friends | -.079* | -.085* | -.044 | .026 | .072 | .025 |
| W3 German friends | -.029 | -.093* | -.061 | -.019 | -.017 | -.036 |
| W4 German friends | -.065 | -.109** | -.049 | -.042 | -.054 | -.030 |
| W5 German friends | -.059 | -.060 | -.060 | -.068 | -.064 | -.082 |
| W6 German friends | .014 | -.005 | .020 | .015 | .007 | .011 |
| W7 German friends | -.032 | -.012 | -.072 | -.041 | -.059 | -.085 |
| W1 National identification | -.117*** | -.137*** | -.069 | -.063 | -.004 | -.053 |
| W2 National identification | -.181*** | -.217*** | -.175*** | -.139** | -.129** | -.203*** |
| W3 National identification | -.088* | -.118** | -.143*** | -.088* | -.127** | -.161** |
| W4 National identification | -.124** | -.130** | -.147*** | -.146*** | -.173*** | -.176** |
| W5 National identification | -.161*** | -.160*** | -.129** | -.184*** | -.135** | -.135* |
| W6 National identification | -.116* | -.095* | -.116* | -.098* | -.138** | -.140* |
| W7 National identification | -.149** | -.120* | -.163** | -.131* | -.143* | -.215*** |

*Note.* W = wave of measurement.
