# Supplementary material for: Religious development from adolescence to early adulthood among Muslim and Christian youth in Germany: A person‐oriented approach
Source: Child Dev. 2024 Aug 27;96(1):141–60. doi: 10.1111/cdev.14151 (PMC11693839; doi:10.1111/cdev.14151)
Supplement: Supplementary file 2 — Data S2. [file CDEV-96-141-s002.docx]

**OSM B – Attrition and non-responses**

Here we report the results of detailed drop-out analyses (Attrition) and descriptive statistics of non-responses among participating adolescents (Non-responses).

**Attrition**

The drop-out analyses were guided by Asendorpf and colleagues’ work on systematic attrition in longitudinal studies (Asendorpf et al., 2014).

The majority of longitudinal studies suffer from an increasing drop-out of respondents. The question is whether or not drop-out is systematic, because systematic attrition can bias the results. It would, for example, be problematic if the most religious adolescents dropped out of our study while less religious adolescents continued to participate. In that case, attrition would have restricted the range of observed religiosity, thereby underestimating levels in religiosity across time.

To analyze missing data, we first checked if the data were missing completely at random (MACR). To do this, we used Little’s test implemented in SPSS, and tested all variables of interest to our research (i.e., demographic characteristics, religiosity, outcomes). Little’s MCAR test was significant: *Χ^2^(df)* = 47,532.89 (42.798), *p* < .001, which means that the data were not MCAR and that attrition was associated with observed and unobserved variables.

In the next step, we therefore compared adolescents who dropped out with those who continued to participate. Asendorpf and colleagues argued that even small and non-significant selective drop-out effects can accumulate over the course of a study. Accordingly, we examined drop-out from wave to wave and across the entire study period. As recommended by Asendorpf and colleagues, we did not only rely on significance but also on effect sizes as even very small effects tend to be significant in very large samples, such as ours. As an indicator of effect size, we report Eta-squared (η^2^) which ranges between 0 and 1. An effect is small when η^2^ ≥ 0.01, medium when η^2^ ≥ 0.06, and large when η^2^ ≥ 0.14 (Cohen, 1988). For ease of interpretation, we highlighted small effects (there were no medium or large effects) in the Tables below in bold.

The results showed that attrition from wave to wave (Tables 1-6) and across the entire study period (Table 7) was unrelated to parents’ religiosity and adolescents’ generational status, religiosity, psychological well-being, general health, and risky and unhealthy behaviors. Drop-out from wave to wave was however associated, albeit inconsistently, with parents’ education and occupational status (ISEI) and adolescents’ age, gender, religious affiliation, cultural values and acculturation. These infrequent effects accumulated over time such that older adolescents, boys, adolescents of lower educated parents, adolescents of parents with a lower occupational status, first generation immigrants and Muslim adolescents were more likely to drop out; as were adolescents with more traditional gender role values, lower levels of tolerance, a stronger emphasis on heritage culture maintenance, lower national identification and fewer German friends. The effects from wave to wave (η^2^s ≤ .021), and even the effects across the entire study period (η^2^s ≤ .040), were however small.

As drop-out was related to some of the observed variables we assumed data to be Missing at Random (MAR), which is a reasonable assumption for longitudinal studies that include a broad range of variables (Asendorpf et al., 2014). We corrected for selective attrition by using Full Information Maximum Likelihood (FIML) estimation.

Table B1. Dropout Wave 1 to Wave 2

|  | Wave 2 | |  |  |
| --- | --- | --- | --- | --- |
|  | Participation | No participation | *F* | η^2^ |
| **Age W1** | **15.21 (0.67)** | **15.42 (0.73)** | **57.07***** | **.014** |
| Female | 0.51 (0.50) | 0.46 (0.50) | 5.63* | .001 |
| **Father education** | **2.12 (0.52)** | **1.96 (0.50)** | **48.53***** | **.013** |
| **Mother education** | **2.04 (0.49)** | **1.91 (0.49)** | **36.78***** | **.010** |
| Parents’ highest ISEI | 45.82 (20.03) | 41.09 (18.35) | 29.75*** | .008 |
| Parent religiosity W1 | 2.82 (0.92) | 2.89 (0.99) | 2.35 | .001 |
| First generation | 0.11 (0.31) | 0.14 (0.35) | 6.99** | .002 |
| Second generation | 0.36 (0.48) | 0.41 (0.49) | 5.51* | .001 |
| Third generation | 0.10 (0.30) | 0.09 (0.28) | 1.73 | .000 |
| Native | 0.43 (0.50) | 0.36 (0.48) | 10.21** | .002 |
| Non-immigrant Christian | 0.53 (0.50) | 0.44 (0.50) | 17.08*** | .004 |
| Immigrant-origin Christian | 0.20 (0.40) | 0.19 (0.39) | 0.07 | .000 |
| Immigrant-origin Muslims | 0.28 (0.45) | 0.37 (0.48) | 22.71*** | .006 |
| Religiosity W1 | 2.53 (1.03) | 2.60 (1.10) | 2.70 | .001 |
| Life satisfaction W1 | 7.49 (2.22) | 7.58 (2.36) | 0.89 | .000 |
| Anxiety W1 | 2.38 (0.64) | 2.27 (0.64) | 16.89*** | .004 |
| Depression W1 | 1.96 (0.75) | 1.89 (0.75) | 5.25* | .001 |
| General health W1 | 4.06 (0.89) | 4.09 (0.88) | 0.67 | .000 |
| Drinking W1 | 1.97 (1.02) | 2.05 (1.10) | 2.18 | .001 |
| Smoking W1 | 1.55 (1.24) | 1.84 (1.50) | 20.32*** | .007 |
| Drugs W1 | 1.09 (0.45) | 1.15 (0.63) | 5.45* | .002 |
| Gender role values W1 | 2.34 (1.43) | 2.18 (1.44) | 7.35** | .002 |
| **Tolerance W1** | **2.67 (1.00)** | **2.37 (0.97)** | **54.50***** | **.013** |
| Heritage culture maintenance W1 | 3.50 (1.05) | 3.67 (1.09) | 14.65*** | .004 |
| German culture adoption W1 | 3.69 (1.07) | 3.58 (1.15) | 5.44* | .001 |
| National identification W1 | 3.14 (0.98) | 2.91 (1.09) | 30.42*** | .008 |
| German friends W1 | 3.74 (1.27) | 3.51 (1.31) | 16.94*** | .004 |

*Notes.* Table shows means and standard deviations (in parentheses) for participating and non-participating adolescents, the results of ANOVAs and effects size η^2^. Small effects highlighted in bold. Variables are described in Appendix A.

* *p* < .05, ** *p* < .01, *** *p* < .001.

Table B2. Dropout Wave 2 to Wave 3

|  | Wave 3 | | |  |
| --- | --- | --- | --- | --- |
|  | Participation | No participation | *F* | η^2^ |
| Age W1 | 15.18 (0.65) | 15.32 (0.71) | 23.10*** | .007 |
| **Female** | **0.54 (0.50)** | **0.41 (0.49)** | **39.63***** | **.012** |
| **Father education** | **2.15 (0.51)** | **2.00 (0.52)** | **36.75***** | **.012** |
| Mother education | 2.06 (0.48) | 1.94 (0.51) | 28.82*** | .009 |
| Parents’ highest ISEI | 46.69 (20.06) | 42.06 (19.52) | 26.18*** | .008 |
| Parent religiosity W1 | 2.81 (0.92) | 2.85 (0.92) | 0.87 | .000 |
| First generation | 0.09 (0.29) | 0.16 (0.37) | 31.66*** | .009 |
| Second generation | 0.36 (0.48) | 0.38 (0.49) | 0.84 | .000 |
| Third generation | 0.11 (0.31) | 0.08 (0.28) | 3.43 | .001 |
| Native | 0.44 (0.50) | 0.38 (0.49) | 10.55** | .003 |
| Non-immigrant Christian | 0.44 (0.50) | 0.38 (0.49) | 10.55** | .003 |
| Immigrant-origin Christian | 0.20 (0.40) | 0.19 (0.39) | 0.47 | .000 |
| **Immigrant-origin Muslims** | **0.26 (0.44)** | **0.36 (0.48)** | **33.94***** | **.010** |
| Religiosity W2 | 2.51 (1.02) | 2.59 (1.11) | 3.65 | .001 |
| Life satisfaction W2 | 7.62 (2.19) | 7.64 (2.33) | 0.03 | .001 |
| Depression W2 | 1.96 (0.77) | 1.80 (0.74) | 21.19*** | .007 |
| General health W2 | 3.98 (0.91) | 4.09 (0.90) | 7.43** | .003 |
| Drinking W2 | 2.22 (1.09) | 2.25 (1.16) | 0.39 | .000 |
| Smoking W2 | 1.72 (1.40) | 1.95 (1.59) | 13.43*** | .004 |
| Drugs W2 | 1.11 (0.50) | 1.17 (0.67) | 5.59* | .002 |
| Gender role values W2 | 2.85 (1.30) | 2.56 (1.36) | 26.61*** | .008 |
| Heritage culture maintenance W2 | 3.52 (1.01) | 3.62 (1.08) | 5.26* | .002 |
| German culture adoption W2 | 3.77 (0.98) | 3.76 (1.05) | 0.20 | .000 |
| **National identification W2** | **3.25 (0.93)** | **3.01 (1.03)** | **35.54***** | **.010** |
| German friends W2 | 3.64 (1.26) | 3.35 (1.30) | 30.20*** | .009 |

*Notes.* Table shows means and standard deviations (in parentheses) for participating and non-participating adolescents, the results of ANOVAs and effects size η^2^. Small effects highlighted in bold. Variables are described in Appendix A.

* *p* < .05, ** *p* < .01, *** *p* < .001.

Table B3. Dropout Wave 3 to Wave 4

|  | Wave 4 | | |  |
| --- | --- | --- | --- | --- |
|  | Participation | No participation | *F* | η^2^ |
| **Age W1** | **15.14 (0.63)** | **15.42 (0.73)** | **55.01***** | **.021** |
| Female | 0.55 (0.50) | 0.47 (0.50) | 7.98** | .003 |
| Father education | 2.16 (0.51) | 2.06 (0.53) | 10.66** | .004 |
| Mother education | 2.08 (0.48) | 1.99 (0.48) | 9.99** | .004 |
| **Parents’ highest ISEI** | **47.57 (20.14)** | **40.85 (18.44)** | **33.52***** | **.013** |
| Parent religiosity W1 | 2.80 (0.92) | 2.89 (0.92) | 2.07 | .001 |
| First generation | 0.08 (0.28) | 0.13 (0.34) | 8.12** | .003 |
| Second generation | 0.35 (0.48) | 0.41 (0.49) | 4.79* | .002 |
| Third generation | 0.11 (0.31) | 0.09 (0.28) | 1.37 | .001 |
| Native | 0.46 (0.50) | 0.37 (0.48) | 9.23** | .003 |
| Non-immigrant Christian | 0.46 (0.50) | 0.37 (0.48) | 9.23** | .003 |
| Immigrant-origin Christian | 0.20 (0.40) | 0.22 (0.41) | 0.86 | .000 |
| Immigrant-origin Muslims | 0.24 (0.43) | 0.33 (0.47) | 10.91*** | .004 |
| Religiosity W3 | 2.46 (1.02) | 2.52 (1.08) | 1.05 | .000 |
| Life satisfaction W3 | 7.96 (1.71) | 7.85 (1.84) | 1.32 | .001 |
| Anxiety W3 | 2.36 (0.69) | 2.32 (0.70) | 0.97 | .000 |
| Depression W3 | 1.83 (0.68) | 1.85 (0.75) | 0.15 | .000 |
| Drinking W3 | 2.43 (1.12) | 2.29 (1.12) | 5.09* | .002 |
| Smoking W3 | 1.77 (1.47) | 2.12 (1.69) | 17.16*** | .006 |
| Tolerance W3 | 3.18 (1.04) | 2.88 (1.08) | 23.63*** | .009 |
| Heritage culture maintenance W3 | 3.62 (0.86) | 3.60 (0.89) | 0.13 | .000 |
| German culture adoption W3 | 3.81 (0.84) | 3.76 (0.87) | 1.21 | .000 |
| National identification W3 | 3.31 (0.84) | 3.14 (0.93) | 11.95*** | .004 |
| German friends W3 | 3.62 (1.25) | 3.41 (1.37) | 8.27** | .003 |

*Notes.* Table shows means and standard deviations (in parentheses) for participating and non-participating adolescents, the results of ANOVAs and effects size η^2^. Small effects highlighted in bold. Variables are described in Appendix A.

* *p* < .05, ** *p* < .01, *** *p* < .001.

Table B4. Dropout Wave 4 to Wave 5

|  | Wave 5 | | |  |
| --- | --- | --- | --- | --- |
|  | Participation | No participation | *F* | η^2^ |
| Age W1 | 15.13 (0.62) | 15.31 (0.68) | 16.29*** | .007 |
| **Female** | **0.57 (0.50)** | **0.36 (0.48)** | **34.61***** | **.015** |
| Father education | 2.17 (0.51) | 2.07 (0.50) | 7.06** | .003 |
| Mother education | 2.08 (0.48) | 2.05 (0.40) | 0.80 | .000 |
| Parents’ highest ISEI | 47.91 (20.15) | 44.30 (19.82) | 6.04* | .003 |
| Parent religiosity W1 | 2.79 (0.92) | 2.89 (0.93) | 1.85 | .001 |
| First generation | 0.08 (0.27) | 0.12 (0.33) | 4.08* | .002 |
| Second generation | 0.35 (0.48) | 0.41 (0.49) | 3.26 | .001 |
| Third generation | 0.11 (0.31) | 0.11 (0.31) | 0.02 | .000 |
| Native | 0.46 (0.50) | 0.37 (0.48) | 7.66** | .003 |
| Non-immigrant Christian | 0.46 (0.50) | 0.37 (0.48) | 7.66** | .003 |
| Immigrant-origin Christian | 0.19 90.39) | 0.24 (0.43) | 2.48 | .001 |
| Immigrant-origin Muslims | 0.24 (0.43) | 0.30 (0.46) | 3.60 | .002 |
| Life satisfaction W4 | 7.24 (2.16) | 7.73 (2.01) | 9.85** | .004 |
| General health W4 | 3.88 (0.92) | 3.99 (0.91) | 2.59 | .001 |
| Drinking W4 | 2.46 (1.03) | 2.41 (1.01) | 0.48 | .000 |
| Smoking W4 | 2.02 (1.57) | 2.29 (1.76) | 5.64* | .002 |
| Drugs W4 | 1.19 (0.62) | 1.23 (0.79) | 0.80 | .000 |
| Gender role values W4 | 3.08 (1.25) | 3.00 (1.38) | 0.80 | .000 |
| National identification W4 | 3.20 (0.90) | 3.53 (1.23) | 0.00 | .000 |
| German friends W4 | 3.71 (1.24) | 3.53 (1.23) | 4.07* | .002 |

*Notes.* Table shows means and standard deviations (in parentheses) for participating and non-participating adolescents, the results of ANOVAs and effects size η^2^. Small effects highlighted in bold. Variables are described in Appendix A.

* *p* < .05, ** *p* < .01, *** *p* < .001.

Table B5. Dropout Wave 5 to Wave 6

|  | Wave 6 | | |  |
| --- | --- | --- | --- | --- |
|  | Participation | No participation | *F* | η^2^ |
| Age W1 | 15.11 (0.61) | 15.19 (0.68) | 5.99* | .003 |
| Female | 0.57 (0.50) | 0.55 (0.50) | 0.83 | .000 |
| Father education | 2.18 (0.51) | 2.12 (0.50) | 4.94* | .002 |
| Mother education | 2.09 (0.49) | 2.04 (0.48) | 3.29 | .002 |
| Parents’ highest ISEI | 48.43 (20.24) | 45.79 (19.67) | 5.54* | .003 |
| Parent religiosity W1 | 2.79 (0.92) | 2.82 (0.91) | 0.40 | .000 |
| First generation | 0.08 (0.26) | 0.10 (0.30) | 2.31 | .001 |
| Second generation | 0.34 (0.47) | 0.38 (0.49) | 2.54 | .001 |
| Third generation | 0.12 (0.32) | 0.09 (0.28) | 2.98 | .001 |
| Native | 0.44 (0.50) | 0.47 (0.50) | 1.61 | .001 |
| Non-immigrant Christian | 0.47 (0.50) | 0.44 (0.50) | 1.61 | .001 |
| Immigrant-origin Christian | 0.19 (0.39) | 0.22 (0.41) | 1.93 | .001 |
| Immigrant-origin Muslims | 0.23 (0.42) | 0.26 (0.44) | 1.65 | .001 |
| Religiosity W5 | 2.31 (1.04) | 2.30 (1.10) | 0.01 | .000 |
| Life satisfaction W5 | 7.54 (2.01) | 7.43 (2.26) | 0.94 | .000 |
| Drinking W5 | 2.39 (1.00) | 2.28 (1.00) | 3.97* | .002 |
| Smoking W5 | 2.14 (1.62) | 2.16 (1.70) | 0.02 | .000 |
| Drugs W5 | 1.22 (0.64) | 1.14 (0.54) | 5.95* | .003 |
| Tolerance W5 | 3.41 (1.07) | 3.34 (1.08) | 1.61 | .001 |
| Heritage culture maintenance W5 | 3.48 (0.98) | 3.48 (1.04) | 0.01 | .000 |
| German culture adoption W5 | 3.92 (0.82) | 3.95 (0.86) | 0.48 | .000 |
| National identification W5 | 3.17 (0.88) | 3.06 (0.93) | 5.00* | .002 |
| German friends W5 | 3.80 (1.16) | 3.66 (1.24) | 4.97* | .002 |

*Notes.* Table shows means and standard deviations (in parentheses) for participating and non-participating adolescents, the results of ANOVAs and effects size η^2^. Small effects highlighted in bold. Variables are described in Appendix A.

* *p* < .05, ** *p* < .01, *** *p* < .001.

Table B6. Dropout Wave 6 to Wave 7

|  | Wave 7 | | |  |
| --- | --- | --- | --- | --- |
|  | Participation | No participation | *F* | η^2^ |
| Age W1 | 15.09 (0.59) | 15.24 (0.68) | 13.94*** | .009 |
| **Female** | **0.60 (0.49)** | **0.44 (0.50)** | **23.82***** | **.014** |
| Father education | 2.20 (0.51) | 2.11 (0.52) | 6.17* | .004 |
| **Mother education** | **2.12 (0.48)** | **1.96 (0.47)** | **24.61***** | **.015** |
| **Parents’ highest ISEI** | **49.38 (20.17)** | **43.58 (19.95)** | **18.62***** | **.011** |
| Parent religiosity W1 | 2.77 (0.91) | 2.88 (0.96) | 2.92 | .002 |
| First generation | 0.07 (0.25) | 0.11 (0.31) | 4.18* | .003 |
| Second generation | 0.32 (0.47) | 0.41 (0.49) | 8.01** | .005 |
| Third generation | 0.12 (0.32) | 0.10 (0.30) | 0.62 | .000 |
| Native | 0.49 (0.50) | 0.38 (0.49) | 10.64** | .006 |
| Non-immigrant Christian | 0.49 (0.50) | 0.38 (0.49) | 10.64** | .006 |
| Immigrant-origin Christian | 0.19 (0.39) | 0.17 (0.37) | 0.71 | .000 |
| **Immigrant-origin Muslims** | **0.21 (0.41)** | **0.36 (0.48)** | **29.12***** | **.017** |
| Religiosity W6 | 2.30 (1.00) | 2.49 (1.08) | 7.99** | .005 |
| Life satisfaction W6 | 7.80 (1.54) | 7.79 (1.61) | 0.02 | .000 |
| General health W6 | 3.68 (0.93) | 3.74 (0.84) | 0.64 | .001 |
| Gender role values W6 | 3.53 (0.91) | 3.36 (1.02) | 7.63** | .005 |
| National identification W6 | 3.36 (0.73) | 3.30 (0.77) | 1.81 | .001 |
| German friends W6 | 3.76 (1.13) | 3.52 (1.14) | 10.77** | .006 |

*Notes.* Table shows means and standard deviations (in parentheses) for participating and non-participating adolescents, the results of ANOVAs and effects size η^2^. Small effects highlighted in bold. Variables are described in Appendix A.

* *p* < .05, ** *p* < .01, *** *p* < .001.

Table B7. Dropout Wave 1 to Wave 7

|  | Wave 2 | |  |  |
| --- | --- | --- | --- | --- |
|  | Participation | No participation | *F* | η^2^ |
| **Age W1** | **15.11 (0.60)** | **15.33 (0.72)** | **105.93***** | **.026** |
| **Female** | **0.60 (0.49)** | **0.44 (0.50)** | **91.95***** | **.022** |
| **Father education** | **2.19 (0.52)** | **2.03 (0.51)** | **84.72***** | **.022** |
| **Mother education** | **2.12 (0.49)** | **1.95 (0.48)** | **105.65***** | **.028** |
| **Parents’ highest ISEI** | **49.02 (20.30)** | **42.29 (19.05)** | **10.853***** | **.028** |
| Parent religiosity W1 | 2.77 (0.91) | 2.88 (0.94) | 11.24*** | .004 |
| **First generation** | **0.07 (0.26)** | **0.14 (0.34)** | **43.94***** | **.011** |
| Second generation | 0.33 (0.47) | 0.40 (0.49) | 15.54*** | .004 |
| Third generation | 0.12 (0.32) | 0.09 (0.28) | 9.68** | .002 |
| Native | 0.48 (0.50) | 0.38 90.49) | 38.65*** | .009 |
| **Non-immigrant Christian** | **0.59 (0.49)** | **0.46 90.50)** | **67.66***** | **.016** |
| Immigrant-origin Christian | 0.19 (0.39) | 0.20 (0.40) | 0.31 | .000 |
| **Immigrant-origin Muslims** | **0.22 (0.41)** | **0.34 (0.47)** | **73.03***** | **.018** |
| Religiosity W1 | 2.49 (0.98) | 2.58 (1.07) | 7.94** | .002 |
| Life satisfaction W1 | 7.48 (2.13) | 7.53 (2.31) | 0.48 | .000 |
| Anxiety W1 | 2.43 (0.64) | 2.32 (0.64) | 26.42*** | .006 |
| Depression W1 | 2.02 (0.75) | 1.91 (0.75) | 20.59*** | .005 |
| General health W1 | 4.01 (0.91) | 4.10 (0.87) | 9.81** | .002 |
| Drinking W1 | 1.96 (1.00) | 2.00 (1.05) | 0.96 | .000 |
| Smoking W1 | 1.46 (1.12) | 1.69 (1.38) | 23.83*** | .008 |
| Drugs W1 | 1.08 (0.40) | 1.12 (0.53) | 4.76* | .002 |
| **Gender role values W1** | **2.49 (1.42)** | **2.20 (1.42)** | **39.47***** | **.010** |
| **Tolerance W1** | **2.87 (0.99)** | **2.46 (0.98)** | **165.45***** | **.040** |
| **Heritage culture maintenance W1** | **3.38 (1.05)** | **3.62 (1.06)** | **47.44***** | **.012** |
| German culture adoption W1 | 3.73 (1.05) | 3.63 (1.10) | 8.37** | .002 |
| **National identification W1** | **3.27 90.92)** | **2.99 (1.04)** | **72.25***** | **.018** |
| **German friends W1** | **3.92 (1.19)** | **3.56 (1.31)** | **73.70***** | **.019** |

*Notes.* Table shows means and standard deviations (in parentheses) for participating and non-participating adolescents, the results of ANOVAs and effects size η^2^. Small effects highlighted in bold. Variables are described in Appendix A.

* *p* < .05, ** *p* < .01, *** *p* < .001.

**Non-responses**

Table 8 shows the non-responses among adolescents who participated in the study. For about a fifth of the parents (22.2%), we had no information on parental religiosity. Among participating adolescents, non-responses for variables relevant to our research ranged from 0.4% to 27.5% at Wave 1, 0.2% to 12.2% at Wave 2, 0.0004% to 0.8% at Wave 3, 0.2% to 1.4% at Wave 4, 0.4% to 2.3% at Wave 5, 0.1% to 31.9% at Wave 6, and 0.5% to 2.5% at Wave 7. Larger non-responses occurred because of the study’s design. The relatively high number of non-responses for drinking, smoking and drug use at Wave 1 and Wave 2 are because these questions were not asked in one of Germany’s federal states. The non-responses for depression and general health at Wave 2 are because these questions were not asked in the German post questionnaire and the short version of the German questionnaire. The high number of non-responses to the general health item in Wave 6 are because this question was only asked in the long version of the questionnaire.

Table B8. Non-responses among participating adolescents.

|  |  | Missing |  |
| --- | --- | --- | --- |
|  | N | Count | Percent |
| Age W1 | 4019 | 61 | 1.5 |
| Female | 4050 | 30 | 0.7 |
| Father education | 3711 | 369 | 9.0 |
| Mother education | 3736 | 344 | 8.4 |
| Parents’ highest ISEI | 3800 | 280 | 6.9 |
| W1 Parent religiosity | 3173 | 907 | 22.2 |
| W1 Religiosity | 4001 | 79 | 1.9 |
| W1 Life satisfaction | 4055 | 25 | 0.6 |
| W1 Anxiety | 4062 | 18 | 0.4 |
| W1 Depression | 4059 | 21 | 0.5 |
| W1 General health | 4051 | 29 | 0.7 |
| W1 Drinking | 2974 | 1106 | 27.1 |
| W1 Smoking | 2971 | 1109 | 27.2 |
| W1 Drugs | 2960 | 1120 | 27.5 |
| W1 Gender role values | 4042 | 38 | 0.9 |
| W1 Tolerance | 4014 | 66 | 1.6 |
| W1 Heritage culture maintenance | 3932 | 148 | 3.6 |
| W1 German culture adoption | 3939 | 141 | 3.5 |
| W1 National identification | 4006 | 74 | 1.8 |
| W1 German friends | 3873 | 207 | 5.1 |
| W2 Religiosity | 3360 | 6 | 0.2 |
| W2 Life satisfaction | 3337 | 29 | 0.9 |
| W2 Depression | 2963 | 403 | 12.0 |
| W2 General health | 2957 | 409 | 12.2 |
| W2 Drinking | 3051 | 315 | 9.4 |
| W2 Smoking | 3049 | 317 | 9.4 |
| W2 Drugs | 3051 | 315 | 9.4 |
| W2 Gender role values | 3354 | 12 | 0.4 |
| W2 Heritage culture maintenance | 3334 | 32 | 1.0 |
| W2 German culture adoption | 3345 | 21 | 0.6 |
| W2 National identification | 3352 | 14 | 0.4 |
| W2 German friends | 3333 | 33 | 1.0 |
| W3 Religiosity | 2750 | 1 | 0.0 |
| W3 Life satisfaction | 2732 | 19 | 0.7 |
| W3 Anxiety | 2730 | 21 | 0.8 |
| W3 Depression | 2728 | 23 | 0.8 |
| W3 Drinking | 2740 | 11 | 0.4 |
| W3 Smoking | 2739 | 12 | 0.4 |
| W3 Tolerance | 2728 | 23 | 0.8 |
| W3 Heritage culture maintenance | 2731 | 20 | 0.7 |
| W3 German culture adoption | 2741 | 10 | 0.4 |
| W3 National identification | 2741 | 10 | 0.4 |
| W3 German friends | 2730 | 21 | 0.8 |
| W4 Life satisfaction | 2436 | 19 | 0.8 |
| W4 General health | 2438 | 17 | 0.7 |
| W4 Drinking | 2438 | 17 | 0.7 |
| W4 Smoking | 2438 | 17 | 0.7 |
| W4 Drugs | 2434 | 21 | 0.9 |
| W4 Gender role values | 2438 | 17 | 0.7 |
| W4 National identification | 2420 | 35 | 1.4 |
| W4 German friends | 2449 | 6 | 0.2 |
| W5 Religiosity | 2259 | 13 | 0.6 |
| W5 Life satisfaction | 2259 | 13 | 0.6 |
| W5 Drinking | 2263 | 9 | 0.4 |
| W5 Smoking | 2260 | 12 | 0.5 |
| W5 Drugs | 2246 | 26 | 1.1 |
| W5 Tolerance | 2242 | 30 | 1.3 |
| W5 Heritage culture maintenance | 2220 | 52 | 2.3 |
| W5 German culture adoption | 2237 | 35 | 1.5 |
| W5 National identification | 2230 | 42 | 1.8 |
| W5 German friends | 2251 | 21 | 0.9 |
| W6 Religiosity | 1849 | 2 | 0.1 |
| W6 Life satisfaction | 1847 | 4 | 0.2 |
| W6 General health | 1261 | 590 | 31.9 |
| W6 Gender role values | 1850 | 1 | 0.1 |
| W6 National identification | 1844 | 7 | 0.4 |
| W6 German friends | 1848 | 3 | 0.2 |
| W7 Religiosity | 1557 | 14 | 0.9 |
| W7 Life satisfaction | 1563 | 8 | 0.5 |
| W7 Anxiety | 1562 | 9 | 0.6 |
| W7 Depression | 1559 | 12 | 0.8 |
| W7 Drinking | 1562 | 9 | 0.6 |
| W7 Smoking | 1557 | 14 | 0.9 |
| W7 Drugs | 1553 | 18 | 1.1 |
| W7 Tolerance | 1561 | 10 | 0.6 |
| W7 Heritage culture maintenance | 1552 | 19 | 1.2 |
| W7 German culture adoption | 1558 | 13 | 0.8 |
| W7 National identification | 1531 | 40 | 2.5 |
| W7 German friends | 1543 | 28 | 1.8 |

**References**

Asendorpf, J. B., Van De Schoot, R., Denissen, J. J., & Hutteman, R. (2014). Reducing bias due to systematic attrition in longitudinal studies: The benefits of multiple imputation. *International Journal of Behavioral Development, 38*(5), 453-460. <https://doi.org/10.1177/0165025414542713>

Cohen, J. (1988). *Statistical power analysis for the behavioral sciences* (Second ed.). New York; London.
