# Supplementary material for: Religious development from adolescence to early adulthood among Muslim and Christian youth in Germany: A person‐oriented approach
Source: Child Dev. 2024 Aug 27;96(1):141–60. doi: 10.1111/cdev.14151 (PMC11693839; doi:10.1111/cdev.14151)
Supplement: Supplementary file 3 — Data S3. [file CDEV-96-141-s001.docx]

**OSM C – Growth models of religiosity**

We started our analyses by estimating six different growth models separately for each religious group: a linear model, a quadratic model, a piecewise model with two intercepts, two piecewise models with a turning point either at Wave 3 or 5, and a latent basis model with free time scores. A linear change would indicate a continuous change in mean levels of religiosity over time. A quadratic change would indicate non-linearity meaning that the rate of change differs across time. For example, changes in religiosity may dampen or accelerate in early adulthood or turn with decreases following increases in religiosity or vice versa. Piecewise models allow us to directly compare changes during adolescence with changes during early adulthood and therefore test which developmental period is more sensitive to changes in religiosity. Latent basis models are flexible non-linear models with free time scores, in which the mean of the slope growth factor describes an average linear change from one time point to another.

Wave 1 data were collected in November 2010, Wave 2 data 13 months later in December 2011, Wave 3 data 13 months later in January 2013, Wave 5 data 25 months later in February 2015, Wave 6 data 16 months later in June 2016, and Wave 7 data 22 months later in April 2018. Given the relatively larger gaps between the later waves, we specified growth models with non-equidistant time points using months between waves / 100. The factor loadings of the intercepts were set to 1 in all models. The time scores for the growth change factors are shown in Table C1.

*Table C1*. Time scores

| Waves |  | 1 | 2 | 3 | 5 | 6 | 7 |
| --- | --- | --- | --- | --- | --- | --- | --- |
| Months between waves |  | 0 | 13 | 13 | 25 | 16 | 22 |
| Months between waves cumulative | | 0 | 13 | 26 | 51 | 67 | 89 |
| 1. Linear | Slope | 0 | 0.13 | 0.26 | 0.51 | 0.67 | 0.89 |
| 1. Quadratic | Slope | 0 | 0.13 | 0.26 | 0.51 | 0.67 | 0.89 |
| 1. Piecewise 2 intercepts | Slope 1 | 0 | 0.13 | 0.26 |  |  |  |
|  | Slope 2 |  |  |  | 0 | 0.16 | 0.38 |
| 1. Piecewise turning point W3 | Slope 1 | 0 | 0.13 | 0.26 | 0.26 | 0.26 | 0.26 |
|  | Slope 2 | 0 | 0 | 0 | 0.25 | 0.41 | 0.63 |
| 1. Piecewise turning point W5 | Slope 1 | 0 | 0.13 | 0.26 | 0.51 | 0.51 | 0.51 |
|  | Slope 2 | 0 | 0 | 0 | 0 | 0.16 | 0.38 |
| 1. Latent basis | Slope | 0 | * | * | * | * | 1 |

Notes. W = Wave; * = freely estimated.

The linear model estimates two growth factors: an intercept (i.e., initial level at Wave 1) and a slope (i.e., linear rate of change from one wave to another). In the quadratic model the factor loadings are the same as in the linear model. The only difference between the linear and the quadratic model is that the quadratic model estimates three (instead of two) growth factors: an intercept, a linear growth factor and a quadratic growth factor. The piecewise growth model with two intercepts estimates four growth factors: an intercept for adolescence (i.e., initial level at Wave 1), a slope for adolescence (i.e., linear rate of change from Wave 1 to Wave 3), an intercept for early adulthood (i.e., initial level at Wave 5), and a slope for early adulthood (i.e., linear rate of change from Wave 5 to Wave 7). The piecewise growth model with a turning point at Wave 3 estimates three growth factors: an intercept (i.e., initial level at Wave 1), a slope for adolescence (i.e., linear rate of change from Wave 1 to Wave 3), and a slope for early adulthood (i.e., linear change from Wave 3 to Wave 7). The piecewise growth model with a turning point at Wave 5 estimates three growth factors: an intercept (i.e., initial level at Wave 1), a slope for adolescence (i.e., linear rate of change from Wave 1 to Wave 5), and a slope for early adulthood (i.e., linear change from Wave 5 to Wave 7). In the latent basis model with free time scores, the factor loadings of the slope were freely estimated by setting them to 0, *, *, *, * and 1. The latent basis model estimates an intercept (i.e., initial level at Wave 1) and a slope (i.e., linear rate of change over the entire study period).

As we used maximum likelihood estimation with robust standard errors (MLR), we compared the fit of the growth models with an adjusted χ^2^ difference tests (Satorra & Bentler, 2001). A visual inspection of the model fit indices (Table C2) indicated that the piecewise growth model with two intercepts fit the data best across the three religious groups. We therefore limited the model comparisons to the piecewise growth model with two intercepts against the other models.

For immigrant-origin Muslim youth, the piecewise model with two intercepts fit better than the linear model: χ^2^ (*df*) = 53.08 (9), *p* < .001, the piecewise model with a turning point at W3: χ^2^ (*df*) = 17.48 (5), *p* = .004, the piecewise model with a turning point at W5: χ^2^ (*df*) = 19.73 (5), *p* = .001, the latent basis model: χ^2^ (*df*) = 56.90 (5), *p* < .001, and by trend the quadratic model: χ^2^ (*df*) = 10.90 (5), *p* = .053. For immigrant-origin Christian youth, the piecewise model with two intercepts fit better than the linear model: χ^2^ (*df*) = 59.71 (9), *p* < .001, the quadratic model: χ^2^ (*df*) = 23.50 (5), *p* < .001, the piecewise model with a turning point at W3: χ^2^ (*df*) = 16.39 (5), *p* = .006, the piecewise model with a turning point at W5: χ^2^ (*df*) = 28.22 (5), *p* < .001, and the latent basis model: χ^2^ (*df*) = 52.37 (5), *p* < .001. For non-immigrant Christian youth, the piecewise model with two intercepts fit better than the linear model: χ^2^ (*df*) = 85.04 (9), *p* < .001, the quadratic model: χ^2^ (*df*) = 28.59 (5), *p* = .005, the piecewise model with a turning point at W3: χ^2^ (*df*) = 34.06 (5), *p* < .001, the piecewise model with a turning point at W5: χ^2^ (*df*) = 30.60 (5), *p* = .006, and the latent basis model: χ^2^ (*df*) = 43.47 (5), *p* < .001. Taken together, these comparisons indicated that a piecewise growth model with two intercepts was the optimal functional form to describe changes in religiosity in this dataset.

*Table C2.* Model fit indices for growth functions of religious development.

|  | X^2^(*df*) | CFI | TLI | RMSEA, 90% CI | SRMR |
| --- | --- | --- | --- | --- | --- |
| Immigrant Muslim |  |  |  |  |  |
| Linear | 66.03 (16)*** | 0.971 | 0.972 | .051 (.039, .064) | .034 |
| Quadratic | 23.29 (12)* | 0.993 | 0.992 | .028 (.010, .045) | .018 |
| Piecewise 2 intercepts | 12.22 (7)* | 0.997 | 0.993 | .025 (.000, .048) | .010 |
| Piecewise turning point W3 | 30.00 (12)** | 0.989 | 0.987 | .035 (.020, .052) | .018 |
| Piecewise turning point W5 | 32.94 (12)** | 0.988 | 0.985 | .038 (.023, .054) | .023 |
| Latent basis | 57.54 (12)*** | 0.973 | 0.966 | .056 (.042, .071) | .037 |
| Immigrant Christian |  |  |  |  |  |
| Linear | 69.04 (16)*** | 0.963 | 0.966 | .065 (.049, .081) | .050 |
| Quadratic | 32.05 (12)** | 0.986 | 0.983 | .046 (.027, .065) | .023 |
| Piecewise 2 intercepts | 8.49 (7) | 0.999 | 0.998 | .016 (.000, .049) | .012 |
| Piecewise turning point W3 | 25.11 (12)* | 0.991 | 0.989 | .037 (.016, .057) | .023 |
| Piecewise turning point W5 | 36.03 (12)*** | 0.983 | 0.979 | .050 (.032, .069) | .027 |
| Latent basis | 57.51 (12)*** | 0.969 | 0.961 | .069 (.052, .087) | .049 |
| Non-immigrant Christian |  |  |  |  |  |
| Linear | 139.98 (16)*** | 0.963 | 0.966 | .061 (.052, .070) | .046 |
| Quadratic | 81.72 (12)*** | 0.979 | 0.974 | .053 (.042, .064) | .034 |
| Piecewise 2 intercepts | 53.95 (7)*** | 0.986 | 0.970 | .057 (.043, .071) | .029 |
| Piecewise turning point W3 | 87.58 (12)*** | 0.978 | 0.972 | .055 (.044, .066) | .034 |
| Piecewise turning point W5 | 84.16 (12)*** | 0.979 | 0.973 | .054 (.043, .065) | .035 |
| Latent basis | 97.80 (12)*** | 0.975 | 0.068 | .059 (.048, .070) | .045 |
