# Supplementary material for: Religious development from adolescence to early adulthood among Muslim and Christian youth in Germany: A person‐oriented approach
Source: Child Dev. 2024 Aug 27;96(1):141–60. doi: 10.1111/cdev.14151 (PMC11693839; doi:10.1111/cdev.14151)
Supplement: Supplementary file 4 — Data S4. [file CDEV-96-141-s003.docx]

**OSM D – Class differences auxiliary variables**

To validate the classes of religious development, we examined if they differed in terms of demographic characteristics, parent religiosity and longitudinal changes in well-being, risky and unhealthy behavior, cultural values and acculturation. To account for the imprecision of class membership, we used the BCH procedure for auxiliary variables measured at Wave 1 (Asparouhov & Muthén, 2014) and the BCH procedure for 3-step mixture modeling for variables measured at multiple occasions (Asparouhov & Muthén, 2021). For the 3-step procedure, we saved the BCH weights for each individual and latent class together with the latent class indicators (i.e., religiosity) and the auxiliary variables (e.g., health). We then specified our auxiliary models and used the BCH weights as training data. Our auxiliary models were latent growth curve models. For each auxiliary model, we explored which functional form or type of change fit best (see section Auxiliary models below). To avoid estimation and convergence problems, we freely estimated the means of the growth factors (e.g., intercepts, slopes) but constrained the variances-covariances of the growth factors and time-specific residuals to be equal across classes. The results for immigrant-origin Muslims are shown in Table D1, the results for immigrant-origin Christians in Table D2 and those for non-immigrant Christians in Table D3. Given the sheer amount of possible effects, we further report the percentage of significant differences between classes of religious development in Table D4.

**Auxiliary models**

We determined the optimal type of growth for our auxiliary variables. Our auxiliary variables were assessed between four and seven times across the study period. A minimum of three measurement points is required to specify a linear type of change, a minimum of four to specify a quadratic change, and a minimum of five for a piecewise model with a turning point. For six measurement points, we also specified a piecewise model with two intercepts. Finally, we examined the fit of a latent basis model in which only two measurement points were fixed, and the other freely estimated.

The linear model estimates two growth factors: an intercept (i.e., initial level at Wave 1) and a slope (i.e., linear rate of change between waves). The quadratic model estimates three growth factors: an intercept, a linear growth factor and a quadratic growth factor. The piecewise growth model with a turning point estimates three growth factors: an intercept, a slope for adolescence and a slope for early adulthood. The piecewise growth model with two intercepts estimates four growth factors: an intercept and a slope for adolescence and an intercept and a slope for early adulthood. The latent basis model estimates an intercept and a slope indicating change across the entire study period.

Wave 1 data were collected in November 2010, Wave 2 data 13 months later in December 2011, Wave 3 data 13 months later in January 2013, Wave 4 data 13 months later in February 2014, Wave 5 data 12 months later in February 2015, Wave 6 data 16 months later in June 2016, and Wave 7 data 22 months later in April 2018. Given the relatively larger gap especially between the later waves, we specified growth models with non-equidistant time points using months between waves / 100. The factor loadings of the intercepts were set to 1 in all models. The time scores for the growth change factors (factor loadings for the slopes) are shown in Table D5.

Model fit was assessed using goodness-of-fit indices including a Root Mean Square Error of Approximation (RMSEA) of less than .05, a Comparative Fit Index (CFI) above .90, a Tucker-Lewis index (TLI) greater than .95, and the Standardized Root Mean square Residual (SRMR) of less than .08 (Hu & Bentler, 1999; Kline, 2010). The model fit statistics for immigrant-origin Muslims are shown in Table D6, the model fit statistics for immigrant-origin Christians in Table D7 and those for non-immigrant Christians in Table D8. To compare the model fit we used an adjusted χ^2^ difference test (Satorra & Bentler, 2001), which is necessary when using maximum likelihood estimation with robust standard errors (MLR). In the comparisons we neglected models that yielded warning messages and focused on the model with the lowest Chi-square value as the reference model.

**Immigrant-origin Muslims**

For anxiety, the linear model had a relatively poor fit. So, we also estimated a latent basis model in which we fixed the variance of the manifest W7 variable to 0. This model fit the data better and was used for further analyses.

For depression, the quadratic model yielded a problem with the quadratic term and the latent basis model could not compute the standard errors. The linear model had an acceptable fit and was used for further analyses.

For life satisfaction, the linear model had an acceptable fit and was used for further analyses. All other models for life satisfaction yielded warnings.

For health, the quadratic model yielded a problem with the quadratic term and the latent basis model did not converge. The linear model of health fit the data well and was used for further analyses.

For gender role values, the latent basis model fit better than the linear model: χ^2^ (*df*) = 29.89 (2), *p* < .001. The comparison of the latent basis and quadratic model was not suitable because of a negative value for the Satorra-Bentler Scaled Chi Square. A closer look at the fit indices of the quadratic model indicated poor fit according to TLI and RMSEA. We thus used the latent basis model of gender role values for further analyses.

For tolerance, the quadratic model produced a warning involving the quadratic term. The latent basis model for tolerance fit the data well and better than the linear model: χ^2^ (*df*) = 22.57 (2), *p* < .001. We therefore used the latent basis model for tolerance.

For drinking, the piecewise model with two intercepts fit better than the linear model: χ^2^ (*df*) = 33.54 (9), *p* < .001, the quadratic model: χ^2^ (*df*) = 14.94 (5), *p* = .011, the piecewise model with a turning point: χ^2^ (*df*) = 11.22 (5), *p* = .047, and the latent basis model: χ^2^ (*df*) = 20.32 (5), *p* = .001. We thus initially used the piecewise model with two intercepts to model changes in drinking. However, in the further analyses, the piecewise model with two intercepts yielded warnings so we used the piecewise model with a turning point instead.

For smoking, the piecewise model with two intercepts fit better than the linear model: χ^2^ (*df*) = 67.96 (9), *p* < .001, the quadratic model: χ^2^ (*df*) = 29.60 (5), *p* < .001, the piecewise model with a turning point: χ^2^ (*df*) = 24.91 (5), *p* < .001, and the latent basis model: χ^2^ (*df*) = 45.03 (5), *p* < .001. We thus used the piecewise model with two intercept to model changes in smoking.

For drugs, the linear model yielded a problem because of a negative variance for the slope (*VAR* = -0.042, *p* = .739), the quadratic model yielded a problem because of a negative residual variance for the manifest Wave 7 variable (*VAR* = -0.006, *p* = .977), and the piecewise model with a turning point yielded a problem involving the slope for early adulthood. The latent basis model did not converge. As the negative residual variance for the manifest Wave 7 variable in the quadratic model was very small, we adjusted the model by fixing the variance to 0. The resulting model fit the data well: χ^2^ (*df*) = 7.11 (7), *p* = .417, CFI = 0.997, TLI = 0.996, RMSEA [90% CI] = .004 [.000, .037], SRMR = .058, and was used for further analyses.

For German culture adoption, the quadratic model yielded a warning due to a problem involving the quadratic term. The linear model did not fit the data. When comparing the piecewise model with a turning point and the latent basis model, the Satorra-Bentler Scaled Chi Square value was negative and thus not helpful. A visual comparison of the fit indices showed that both models fit the data well. We settled on the latent basis model for further analyses.

For heritage culture maintenance, the piecewise model with a turning point and the linear model fit the data equally well: χ^2^ (*df*) = 3.34 (4), *p* = .503. We could not compare the piecewise and the quadratic model (same degrees of freedom) and the piecewise and the latent basis model (negative Satorra-Bentler Scaled Chi Square). We used the linear model for further analyses as a visual inspection of the fit indices suggested that the linear model fit slightly better than the piecewise model in terms of TLI and RMSEA.

For German friends, the latent basis model did not converge. The piecewise model with two intercepts fit better than the linear model: χ^2^ (*df*) = 66.83 (9), *p* < .001, the quadratic model: χ^2^ (*df*) = 20.77 (5), *p* = .001, and the piecewise model with a turning point: χ^2^ (*df*) = 19.34 (5), *p* = .002. So, we used the piecewise model with two intercepts for further analyses.

For national identification, the latent basis model was the only model with an acceptable fit, which is why we used the latent basis model for further analyses.

**Immigrant-origin Christians**

For anxiety, the linear model yielded an error message but the latent basis model in which we fixed the variance of the manifest W7 variable to 0 fit the data well and was used for further analyses.

For depression, the liner and latent basis models yielded error messages. The quadratic model fit the data well and was initially used for further analyses. However, when using the quadratic model to examine if the classes of religious development differed in terms of depression trajectories, both the quadratic and the linear models yielded errors. We therefore referred to the latent basis model which did not cause any issues.

For life satisfaction, only the piecewise model with two intercepts had a good fit and was therefore used for further analyses.

For health, the quadratic model fit better than the linear model: χ^2^ (*df*) = 11.22 (4), *p* = .024, and the latent basis model: χ^2^ (*df*) = 13.85 (2), *p* = .001, and was thus used for further analyses.

For gender role values, the quadratic model yielded an error and the latent basis model fit better than the linear model: χ^2^ (*df*) = 33.58 (2), *p* < .001. So, we used the latent basis model for further analyses.

For tolerance, the quadratic model produced a warning involving the linear slope. The latent basis model for tolerance fit the data well and better than the linear model: χ^2^ (*df*) = 24.84 (2), *p* < .001. We therefore used the latent basis model for tolerance.

For drinking, the piecewise model with two intercepts fit better than the linear model: χ^2^ (*df*) = 100.72 (9), *p* < .001, the quadratic model: χ^2^ (*df*) = 38.60 (5), *p* < .001, and the piecewise model with a turning point: χ^2^ (*df*) = 35.32 (5), *p* < .001. The latent basis model yielded an error message. We therefore used the piecewise model with two intercepts for further analyses.

For smoking, the linear model had a poor fit and the quadratic and piecewise models yielded error messages. The latent basis model had an acceptable fit and was thus used for further analyses.

For drugs, the linear model had an acceptable fit. The quadratic model yielded an error. The piecewise model with a turning point fit the data well and better than the latent basis model: χ^2^ (*df*) = 5.05 (1), *p* = .025. For further analyses, we used the piecewise model with a turning point.

For German culture adoption, the latent basis model fit the data well and better than the linear model: χ^2^ (*df*) = 11.09 (3), *p* = .011. We could not compare the latent basis model with the quadratic and the piecewise model with a turning point due to negative Satorra-Bentler Chi-Square values. A visual inspection of the fit indices suggested that the latent basis model had the best fit, which is why we used it for further analyses.

For heritage culture maintenance, the piecewise model with a turning point fit the data well and better than the linear model: χ^2^ (*df*) = 13.56 (4), *p* = .009. We could not compare the piecewise model to the quadratic model because they had the same degrees of freedom. A visual inspection of the fit indices suggested that the piecewise model fit the data slightly better than the quadratic model. The latent basis model did not converge. We thus used the piecewise model for further analyses.

For German friends, the piecewise model with two intercepts fit the data well and better than the linear model: χ^2^ (*df*) = 85.96 (9), *p* < .001, the quadratic model: χ^2^ (*df*) = 17.43 (5), *p* = .004, and the piecewise model with a turning point: χ^2^ (*df*) = 26.54 (5), *p* < .001. The latent basis model did not converge. So, we used the piecewise model with two intercepts for further analyses.

For national identification, the latent basis model was the only model with an acceptable fit, which is why we used the latent basis model for further analyses.

**Non-immigrant Christians**

For anxiety, the linear model had an acceptable fit. The latent basis model in which we fixed the variance of the manifest W7 variable to 0 fit the data better and was used for further analyses.

For depression, the quadratic and latent basis models yielded error messages. The linear model had an acceptable fit and was used for further analyses.

For life satisfaction, the piecewise model with two intercepts had the most acceptable fit and was therefore used for further analyses.

For health, all models fit the data well and no model fit better than another: quadratic and linear: χ^2^ (*df*) = 6.85 (4), *p* = .144, quadratic and latent basis: χ^2^ (*df*) = 3.14 (2), *p* = .208, and linear and latent basis: χ^2^ (*df*) = 3.74 (2), *p* = .154. We used the linear model for further analyses.

For gender role values, the latent basis model had a good and better fit than the linear model: χ^2^ (*df*) = 75.81 (2), *p* < .001. The quadratic model yielded an error message. So, we used the latent basis model for further analyses.

For tolerance, the linear model had a poor fit but the quadratic model fit well and better than the latent basis model: χ^2^ (*df*) = 7.90 (2), *p* = .019. We therefore used the quadratic model for tolerance.

For drinking, the linear model fit poorly and the other models yielded errors. A visual inspection of the fit indices suggested that the piecewise model with two intercepts fit the data best. The error message of the piecewise model resulted from a negative residual variance of the manifest Wave 7 variable which we addressed by fixing it to zero. This adjusted piecewise model with two intercepts had a good fit: χ^2^ *(df)* = 63.46(8), *p* < .001, CFI = 0.970, TLI = 0.944, RMSEA [90% CI] = .059 [.046, .073], SRMR = .025, and was used for further analyses.

For smoking, the piecewise model with two intercepts fit well and better than the linear model: χ^2^ (*df*) = 194.71 (9), *p* < .001, the quadratic model: χ^2^ (*df*) = 42.55 (5), *p* < .001, the piecewise model with a turning point: χ^2^ (*df*) = 42.85 (5), *p* < .001, and the latent basis model: χ^2^ (*df*) = 89.25 (5), *p* < .001. We therefore used the piecewise model with two intercepts for further analyses.

For drugs, the quadratic model prompted an error. The piecewise model with a turning point had a good and better fit than the linear model: χ^2^ (*df*) = 36.46 (4), *p* < .001, and fit as well as the latent basis model: χ^2^ (*df*) = 0.72 (1), *p* = .395. For further analyses, we used the piecewise model with a turning point.

*Table D1.* Classes of religious development and changes in well-being, risk behavior, cultural values and acculturation among Muslim immigrant-origin adolescents

|  | C1 High religiosity (58%) | C2 Low religiosity (31%) | C3 Increasing religiosity (11%) |
| --- | --- | --- | --- |
| Female | 0.44 (0.02)_a_ | 0.56 (0.03)_b_ | 0.52 (0.07)_a,b_ |
| Immigrant generation | 1.84 (0.02) | 1.87 (0.03) | 1.83 (0.08) |
| Mother education | 1.70 (0.03)_a_ | 1.84 (0.04)_b_ | 1.75 (0.09)_a,b_ |
| Father education | 1.91 (0.03) | 1.93 (0.04) | 1.91 (0.09) |
| ISEI | 34.87 (0.76) | 35.78 (1.12) | 34.38 (2.71) |
| Parent religiosity | 3.82 (0.03)_a_ | 3.03 (0.07)_b_ | 3.45 (0.13)_c_ |
| Anxiety |  |  |  |
| Intercept | 2.24 (0.03)***_a_ | 2.37 (0.04)***_b_ | 2.20 (0.08)***_a,b_ |
| Slope | 0.28 (0.07)***_a_ | 0.37 (0.09)***_a_ | -0.20 (0.20)_b_ |
| Depression |  |  |  |
| Intercept | 1.78 (0.03)***_a_ | 1.97 (0.05)***_b_ | 1.77 (0.09)***_a,b_ |
| Slope | 0.02 (0.07) | 0.09 (0.10) | -0.32 (0.19) |
| Life satisfaction |  |  |  |
| Intercept | 7.71 (0.08)*** | 7.43 (0.13)*** | 8.02 (0.30)*** |
| Slope | -0.17 (0.16) | -0.07 (0.23) | 0.40 (0.56) |
| Health |  |  |  |
| Intercept | 4.25 (0.04)*** | 4.14 (0.05)*** | 4.29 (0.11)*** |
| Slope | -0.29 (0.10)**_a_ | -0.67 (0.15)***_b_ | -0.23 (0.31)_a,b_ |
| Gender role values |  |  |  |
| Intercept | 1.61 (0.05)***_a_ | 2.21 (0.09)***_b_ | 1.94 (0.20)***_a,b_ |
| Slope | 1.35 (0.09)*** | 1.18 (0.11)*** | 0.98 (0.34)** |
| Tolerance |  |  |  |
| Intercept | 1.49 (0.02)***_a_ | 1.91 (0.04)***_b_ | 1.70 (0.07 )***_c_ |
| Slope | 0.43 (0.05)***_a_ | 0.81 (0.08)***_b_ | 0.35 (0.16)*_a_ |
| Drinking |  |  |  |
| Intercept | 1.27 (0.03)***_a_ | 1.61 (0.06)***_b_ | 1.55 (0.12)***_b_ |
| Slope adolescence | 0.12 (0.12)_a_ | 1.07 (0.21)***_b_ | 0.29 (0.44)_a,b_ |
| Slope early adulthood | 0.10 (0.12) | -0.02 (0.17) | -0.18 (0.51) |
| Smoking |  |  |  |
| Intercept adolescence | 1.46 (0.5)*** | 1.57 (0.09)*** | 1.82 (0.20)*** |
| Slope adolescence | 0.23 (0.28) | 0.99 (0.46)* | 1.56 (0.87) |
| Intercept early adulthood | 1.82 (0.08)*** | 2.10 (0.14)*** | 2.20 (0.26)*** |
| Slope early adulthood | 0.79 (0.25)** | 0.68 (0.35) | 2.55 (0.93)** |
| Drugs |  |  |  |
| Intercept | 1.12 (0.03)*** | 1.12 (0.04)*** | 1.14 (0.08)*** |
| Slope | -0.35 (0.16)* | -0.09 (0.22) | 0.75 (0.52) |
| Quadratic term | 0.44 (0.19)* | 0.15 (0.25) | -0.71 (0.55) |
| German culture adoption |  |  |  |
| Intercept | 3.32 (0.05)***_a_ | 3.62 (0.07)***_b_ | 3.80 (0.18)***_b_ |
| Slope | 0.44 (0.07)*** | 0.44 (0.10)*** | 0.03 (0.21) |
| Heritage culture maintenance |  |  |  |
| Intercept | 4.28 (0.03)***_a_ | 3.94 (0.05)***_b_ | 4.56 (0.10)***_c_ |
| Slope | -0.25 (0.08)** | -0.42 (0.12)** | -0.76 (0.28)** |
| German friends |  |  |  |
| Intercept adolescence | 2.75 (0.05)*** | 2.93 (0.08)*** | 2.96 (0.18)*** |
| Slope adolescence | -0.30 (0.30) | -0.30 (0.45) | -1.24 (0.99) |
| Intercept early adulthood | 2.60 (0.06)***_a_ | 2.90 (0.10)***_b_ | 2.80 (0.20)***_a,b_ |
| Slope early adulthood | 0.09 (0.18) | -0.31 (0.27) | 0.50 (0.76) |
| National identification |  |  |  |
| Intercept | 2.23 (0.04)***_a_ | 2.51 (0.06)***_b_ | 2.59 (0.12)***_b_ |
| Slope | 0.49 (0.06)*** | 0.48 (0.07)*** | 0.23 (0.16) |

*Notes.* Table shows Means (*M*) and Standard Errors (*SE*) in parentheses. Different subscripts in a row indicate a significant (*p* < .05) difference between two classes. Female coded 0 (*male*), 1 (*female*). Parental education ranged from 1 (*primary education*), 2 (*secondary education*) to 3 (*tertiary education*). Immigrant generation ranged from 1 (*first*), 2 (*second*) to 3 (*third or higher*).

* *p* < .05, ** *p* < .01, *** *p* < .001.

*Table D2.* Classes of religious development and changes in well-being, risk behavior, cultural values and acculturation among Christian immigrant-origin adolescents

|  | C1 Low religiosity (68%) | C2 High religiosity (32%) |
| --- | --- | --- |
| Female | 0.50 (0.03) | 0.53 (0.04) |
| Immigrant generation | 1.79 (0.02)_a_ | 1.65 (0.04)_b_ |
| Mother education | 2.08 (0.03) | 2.09 (0.04) |
| Father education | 2.11 (0.03) | 2.06 (0.05) |
| ISEI | 45.34 (1.00)_a_ | 39.86 (1.66)_b_ |
| Parent religiosity | 2.55 (0.05)_a_ | 3.48 (0.07)_b_ |
| Anxiety |  |  |
| Intercept | 2.45 (0.03)*** | 2.35 (0.05)*** |
| Slope | 0.17 (0.05)** | 0.27 (0.09)** |
| Depression |  |  |
| Intercept | 2.02 (0.05)*** | 1.99 (0.08)*** |
| Slope | -0.05 (.16) | -0.10 (0.32) |
| Life satisfaction |  |  |
| Intercept adolescence | 7.37 (0.10)*** | 7.53 (0.18)*** |
| Slope adolescence | 1.36 (0.48)** | 2.89 (0.84)** |
| Intercept early adulthood | 7.19 (0.12)*** | 7.51 (0.18)*** |
| Slope early adulthood | 0.69 (0.33)* | 0.44 (0.65) |
| Health |  |  |
| Intercept | 3.92 (0.57)***_a_ | 4.13 (0.07)***_b_ |
| Slope | 0.09 (0.32) | -0.65 (0.60) |
| Quadratic term | -0.60 (0.50) | 0.60 (1.01) |
| Gender role values |  |  |
| Intercept | 2.45 (0.06)***_a_ | 2.20 (0.10)***_b_ |
| Slope | 0.99 (0.08)*** | 0.86 (0.1 )*** |
| Tolerance |  |  |
| Intercept | 2.84 (0.05)***_a_ | 2.13 (0.07)***_b_ |
| Slope | 1.12 (0.06)***_a_ | 0.67 (0.11)***_b_ |
| Drinking |  |  |
| Intercept adolescence | 2.30 (0.06)***_a_ | 1.88 (0.10)***_b_ |
| Slope adolescence | 1.42 90.29)*** | 1.34 (0.47)** |
| Intercept early adulthood | 2.67 (0.05)***_a_ | 2.26 (0.09)***_b_ |
| Slope early adulthood | 0.12 (0.14) | -0.10 90.26) |
| Smoking |  |  |
| Intercept | 1.71 (0.08)*** | 1.73 (0.13)*** |
| Slope | 1.01 (0.13)***_a_ | 0.49 (0.16)**_b_ |
| Drugs |  |  |
| Intercept | 1.09 (0.13) | 1.09 (0.04)*** |
| Slope adolescence | 0.49 (0.12)*** | 0.16 (0.17) |
| Slope early adulthood | 0.50 (0.14)***_a_ | -0.14 (0.15)_b_ |
| German culture adoption |  |  |
| Intercept | 3.65 (0.05)*** | 3.72 (0.08)*** |
| Slope | 0.24 (0.07)** | 0.41 (0.09)*** |
| Heritage culture maintenance |  |  |
| Intercept | 3.38 (0.05)***_a_ | 3.90 (0.07)***_b_ |
| Slope adolescence | 0.66 (0.24)**_a_ | -0.78 (0.35)*_b_ |
| Slope early adulthood | -0.55 (0.13)***_a_ | 0.04 (0.21)_b_ |
| German friends |  |  |
| Intercept adolescence | 3.49 (0.06)*** | 3.42 (0.10)*** |
| Slope adolescence | -0.50 (0.28) | -1.43 (0.50)** |
| Intercept early adulthood | 3.45 (0.07)***_a_ | 3.13 (0.11)***_b_ |
| Slope early adulthood | 0.74 (0.18)*** | 0.51 (0.29) |
| National identification |  |  |
| Intercept | 2.94 (0.05)***_a_ | 2.37 (0.08)***_b_ |
| Slope | 0.26 (0.05)*** | 0.43 (0.09)*** |

*Notes.* Table shows Means (*M*) and Standard Errors (*SE*) in parentheses. Different subscripts in a row indicate a significant (*p* < .05) difference between two classes. Female coded 0 (*male*), 1 (*female*). Parental education ranged from 1 (*primary education*), 2 (*secondary education*) to 3 (*tertiary education*). Immigrant generation ranged from 1 (*first*), 2 (*second*) to 3 (*third or higher*).

* *p* < .05, ** *p* < .01, *** *p* < .001.

*Table D3.* Classes of religious development and changes in well-being, risk behavior, cultural values and acculturation among non-immigrant Christian adolescents

|  | C1 Low religiosity (74%) | C3 Decreasing religiosity (17%) | C2 High religiosity (9%) |
| --- | --- | --- | --- |
| Female | 0.48 (0.01)_a_ | 0.55 (0.04)_a,b_ | 0.64 (0.05)_b_ |
| Mother education | 2.12 (0.01)_a_ | 2.11 (0.03)_a,b_ | 2.22 (0.04)_b_ |
| Father education | 2.18 (0.01) | 2.18 (0.04) | 2.26 (0.05) |
| ISEI | 49.53 (0.54)_a_ | 51.97 (1.60)_a,b_ | 56.51 (1.94)_b_ |
| Parent religiosity | 2.35 (0.02)_a_ | 2.59 (0.07)_b_ | 3.35 (0.09)_c_ |
| Anxiety |  |  |  |
| Intercept | 2.35 (0.02)***_a_ | 2.39 (0.05)***_a,b_ | 2.49 (0.06)***_b_ |
| Slope | 0.22 (0.03)***_a_ | 0.34 (0.08)***_a_ | 0.01 (0.08)_b_ |
| Depression |  |  |  |
| Intercept | 1.93 (0.02)*** | 1.98 (0.06)*** | 1.96 (0.06)*** |
| Slope | 0.00 (0.04) | 0.16 (0.10) | -0.01 (0.10) |
| Life satisfaction |  |  |  |
| Intercept adolescence | 7.52 (0.06)***_a_ | 7.44 (0.17)***_a_ | 8.08 (0.20)***_b_ |
| Slope adolescence | 1.60 (0.28)*** | 1.15 (0.86) | 0.83 (0.90) |
| Intercept early adulthood | 7.56 (0.06)***_a_ | 7.25 (0.21)***_a_ | 7.99 (0.19)***_b_ |
| Slope early adulthood | 0.30 (0.18) | 0.62 (0.59) | 0.45 (0.56) |
| Health |  |  |  |
| Intercept | 4.01 (0.02)*** | 4.13 (0.07)*** | 4.00 (0.09)*** |
| Slope | -0.58 (0.06)*** | -0.61 (0.18)** | -0.42 (0.19)* |
| Gender role values |  |  |  |
| Intercept | 2.49 (0.04)*** | 2.47 (0.11)*** | 2.35 (0.14)*** |
| Slope | 1.14 (0.04)*** | 0.99 (0.13)*** | 1.31 (0.14)*** |
| Tolerance |  |  |  |
| Intercept | 3.10 (0.02)***_a_ | 3.02 (0.07)***_a,b_ | 2.89 (0.10)***_b_ |
| Slope | 2.04 (0.10)***_a_ | 2.67 90.30)***_a_ | 1.23 (0.36)**_b_ |
| Quadratic | -1.16 (0.11)*** | -1.47 (0.32)*** | -0.58 (0.36) |
| Drinking |  |  |  |
| Intercept adolescence | 2.34 (0.03)***_a_ | 2.14 (0.10)***_a,b_ | 2.06 (0.12)***_b_ |
| Slope adolescence | 2.02 (0.15)***_a_ | 3.45 (0.45)***_b_ | 2.65 (0.53)***_a,b_ |
| Intercept early adulthood | 2.79 (0.03)*** | 2.72 (0.09)*** | 2.78 (0.09)*** |
| Slope early adulthood | -0.16 (0.08)*_a_ | 0.32 (0.21)_b_ | -0.06 (0.20)_a,b_ |
| Smoking |  |  |  |
| Intercept adolescence | 1.73 (0.04)***_a_ | 1.45 (0.11)***_b_ | 1.37 (0.12)***_b_ |
| Slope adolescence | 1.08 (0.17)*** | 1.65 (0.54)** | 1.22 (0.51)* |
| Intercept early adulthood | 2.26 (0.05)***_a_ | 2.19 (0.15)***_a,b_ | 1.80 (0.14)***_b_ |
| Slope early adulthood | 0.11 (0.11) | 0.77 (0.33)* | 0.31 (0.26) |
| Drugs |  |  |  |
| Intercept | 1.11 (0.01)*** | 1.05 (0.04)*** | 1.08 (0.04)*** |
| Slope adolescence | 0.28 (0.06)*** | 0.45 (0.20)* | 0.32 (0.19) |
| Slope early adulthood | 0.34 (0.07)***_a_ | 0.25 (0.15)_a_ | -0.29 (0.13)*_b_ |

*Notes.* Table shows Means (*M*) and Standard Errors (*SE*) in parentheses. Different subscripts in a row indicate a significant (*p* < .05) difference between two classes. Female coded 0 (*male*), 1 (*female*). Parental education ranged from 1 (*primary education*), 2 (*secondary education*) to 3 (*tertiary education*).

* *p* < .05, ** *p* < .01, *** *p* < .001.

*Table D4.* Percentage of significant differences between classes of religious development

|  | Immigrant-origin Muslim | Immigrant-origin Christian | Non-immigrant Christian |
| --- | --- | --- | --- |
| Overall | 41% | 34% | 46% |
| Wellbeing | 50% | 9% | 40% |
| Risky and unhealthy behavior | 20% | 44% | 55% |
| Cultural values | 75% | 50% | 40% |
| Acculturation | 40% | 46% | - |

*Table D5.* Time scores for growth change factors

| Waves |  | 1 | 2 | 3 | 4 | 5 | 6 | 7 |
| --- | --- | --- | --- | --- | --- | --- | --- | --- |
| Months between waves |  | 0 | 13 | 13 | 13 | 12 | 16 | 22 |
| Months between waves cumulative | | 0 | 13 | 26 | 39 | 51 | 67 | 89 |
| Anxiety (W1, W3, W7) | |  |  |  |  |  |  |  |
| Linear |  | 0 | - | 0.26 | - | - | - | 0.89 |
| Latent basis |  | 0 | - | * | - | - | - | 1 |
| Depression (W1-3, W7) |  |  |  |  |  |  |  |  |
| Linear |  | 0 | 0.13 | 0.26 | - | - | - | 0.89 |
| Quadratic |  | 0 | 0.13 | 0.26 | - | - | - | 0.89 |
| Latent basis |  | 0 | * | * | - | - | - | 1 |
| Health, gender role values (W1, W2, W4, W6) | | | | | | | | |
| Linear |  | 0 | 0.13 | - | 0.39 | - | 0.67 | - |
| Quadratic |  | 0 | 0.13 | - | 0.39 | - | 0.67 | - |
| Latent basis |  | 0 | * | - | * | - | 1 | - |
| Tolerance (W1, W3, W5, W7) |  |  |  |  |  |  |  |  |
| Linear |  | 0 | - | 0.26 | - | 0.51 | - | 0.89 |
| Quadratic |  | 0 | - | 0.26 | - | 0.51 | - | 0.89 |
| Latent basis |  | 0 | - | * | - | * | - | 1 |
| Drugs (W1, W2, W4, W5, W7) |  |  |  |  |  |  |  |  |
| Linear |  | 0 | 0.13 | - | 0.39 | 0.51 | - | 0.89 |
| Quadratic |  | 0 | 0.13 | - | 0.39 | 0.51 | - | 0.89 |
| Piecewise turning point W4 | Slope 1 | 0 | 0.13 | - | 0.39 | 0.39 | - | 0.39 |
|  | Slope 2 | 0 | 0 | - | 0 | 0.12 | - | 0.50 |
| Latent basis |  | 0 | * | - | * | * | - | 1 |
| German culture adoption, heritage culture maintenance (W1-3, W5, W7) | | | | | | | | |
| Linear |  | 0 | 0.13 | 0.26 | - | 0.51 | - | 0.89 |
| Quadratic |  | 0 | 0.13 | 0.26 | - | 0.51 | - | 0.89 |
| Piecewise turning point W3 | Slope 1 | 0 | 0.13 | 0.26 | - | 0.26 | - | 0.26 |
|  | Slope 2 | 0 | 0 | 0 | - | 0.25 | - | 0.63 |
| Latent basis |  | 0 | * | * | - | * | - | 1 |
| Drinking, Smoking (W1-5, W7) |  |  |  |  |  |  |  |  |
| Linear |  | 0 | 0.13 | 0.26 | 0.39 | 0.51 | - | 0.89 |
| Quadratic |  | 0 | 0.13 | 0.26 | 0.39 | 0.51 | - | 0.89 |
| Piecewise turning point W4 | Slope 1 | 0 | 0.13 | 0.26 | 0.39 | 0.39 | - | 0.39 |
|  | Slope 2 | 0 | 0 | 0 | 0 | 0.12 | - | 0.50 |
| Piecewise 2 intercepts | Slope 1 | 0 | 0.13 | 0.26 | - | - | - | - |
|  | Slope 2 | - | - | - | 0 | 0.12 | - | 0.50 |
| Latent basis |  | 0 | * | * | * | * | - | 1 |
| Life satisfaction, German friends, national identification (W1-7) | | | | | | | | |
| Linear | Slope | 0 | 0.13 | 0.26 | 0.39 | 0.51 | 0.67 | 0.89 |
| Quadratic | Slope | 0 | 0.13 | 0.26 | 0.39 | 0.51 | 0.67 | 0.89 |
| Piecewise turning point W4 | Slope 1 | 0 | 0.13 | 0.26 | 0.39 | 0.39 | 0.39 | 0.39 |
|  | Slope 2 | 0 | 0 | 0 | 0 | 0.12 | 0.28 | 0.50 |
| Piecewise 2 intercepts | Slope 1 | 0 | 0.13 | 0.26 | - | - | - | - |
|  | Slope 2 | - | - | - | 0 | 0.12 | 0.28 | 0.50 |
| Latent basis | Slope | 0 | * | * | * | * | * | 1 |

Notes. W = Wave; * = freely estimated, - = no measurement available.

*Table D6*. Model fit indices for growth functions of auxiliary variables among immigrant-origin Muslims.

|  | X^2^(*df*) | CFI | TLI | RMSEA, 90% CI | SRMR |
| --- | --- | --- | --- | --- | --- |
| Anxiety |  |  |  |  |  |
| Linear | 18.43(1)*** | 0.863 | 0.589 | .121 (.077, .172) | .049 |
| Latent basis | 7.28(1) | 0.951 | 0.852 | .072 (.030, .126) | .038 |
| Depression |  |  |  |  |  |
| Linear | 32.81(5)*** | 0.924 | 0.909 | .068 (.047, .091) | .042 |
| Quadratic^1^ | 7.08(1)** | 0.983 | 0.900 | .071 (.029, .125) | .020 |
| Latent basis^2^ | - | - | - | - | - |
| Life satisfaction |  |  |  |  |  |
| Linear | 67.03(23)*** | 0.841 | 0.854 | .040 (.029, 0.51) | .074 |
| Quadratic^1^ | 56.42(19)*** | 0.865 | 0.850 | .041 (.029, .053) | .069 |
| Piecewise turning point^1^ | 17.73(14) | 0.987 | 0.980 | .015 (.000, .033) | .033 |
| Piecewise 2 intercepts^1^ | 57.72(19)*** | 0.860 | 0.845 | .041 (.029, .054) | .070 |
| Latent basis^1^ | 26.08(18) | 0.971 | 0.966 | .019 (.000, .035) | .054 |
| Health |  |  |  |  |  |
| Linear | 16.15(5)** | 0.965 | 0.958 | .043 (.021, .068) | .037 |
| Quadratic^1^ | 0.23(1) | 1.000 | 1.000 | .000 (.000, .060) | .004 |
| Latent basis^3^ | - | - | - | - | - |
| Gender role values |  |  |  |  |  |
| Linear | 42.82(5)*** | 0.929 | 0.915 | .079 (.059, .102) | .043 |
| Quadratic | 30.21(1)*** | 0.945 | 0.670 | .156 (.111, .206) | .037 |
| Latent basis | 9.22(3)* | 0.988 | 0.977 | .042 (.013, .074) | .024 |
| Tolerance |  |  |  |  |  |
| Linear | 27.45(5)*** | 0.959 | 0.951 | .062 (.040, .085) | .039 |
| Quadratic^1^ | 14.13(1)*** | 0.976 | 0.857 | .105 (.061, .157) | .028 |
| Latent basis | 3.31(3) | 0.999 | 0.999 | .009 (.000, .051) | .030 |
| Drinking |  |  |  |  |  |
| Linear | 52.70(16)*** | 0.926 | 0.931 | .045 (.032, .059) | .062 |
| Quadratic | 33.09(12)*** | 0.958 | 0.947 | .040 (.024, .056) | .047 |
| Piecewise turning point | 28.64(12)** | 0.966 | 0.958 | .035 (.019, .052) | .045 |
| Piecewise 2 intercepts | 17.71(7)* | 0.978 | 0.954 | .037 (.016, .059) | .036 |
| Latent basis | 37.85(12)*** | 0.948 | 0.935 | .044 (.029, .060) | .061 |
| Smoking |  |  |  |  |  |
| Linear | 90.32(16)*** | 0.891 | 0.897 | .065 (.052, .078) | .126 |
| Quadratic | 50.57(12)*** | 0.943 | 0.929 | .054 (.039, .070) | .043 |
| Piecewise turning point | 44.88(12)*** | 0.952 | 0.939 | .050 (.035, .066) | .035 |
| Piecewise 2 intercepts | 17.73(7)* | 0.984 | 0.966 | .037 (.016, .059) | .028 |
| Latent basis | 62.05(12)*** | 0.926 | 0.908 | .061 (.047, .077) | .093 |
| Drugs |  |  |  |  |  |
| Linear^1^ | 20.25(10)*** | 0.765 | 0.765 | .031 (.010, .050) | .087 |
| Quadratic^1^ | 8.32(6) | 0.947 | 0.911 | .019 (.000, .046) | .058 |
| Piecewise turning point^1^ | 8.72(6) | 0.938 | 0.896 | .020 (.000, .047) | .067 |
| Latent basis^3^ | - | - | - | - | - |
| German culture adoption |  |  |  |  |  |
| Linear | 98.75(10)*** | 0.582 | 0.582 | .087 (.071, .102) | .149 |
| Quadratic^1^ | 51.11(6)*** | 0.778 | 0.631 | .081 (.062, .102) | .091 |
| Piecewise turning point | 15.76(6)* | 0.954 | 0.923 | .037 (.015, .060) | .032 |
| Latent basis | 16.09(7)* | 0.957 | 0.939 | .033 (.011, .055) | .035 |
| Heritage culture maintenance |  |  |  |  |  |
| Linear | 18.65(10)* | 0.959 | 0.959 | .027 (.004, .046) | .046 |
| Quadratic | 17.41(6)** | 0.946 | 0.909 | .040 (.019, .063) | .044 |
| Piecewise turning point | 14.78(6)* | 0.958 | 0.930 | .035 (.012, .058) | .042 |
| Latent basis | 16.64(7)* | 0.954 | 0.934 | .034 (.013, .056) | .052 |
| German friends |  |  |  |  |  |
| Linear | 106.17(23)*** | 0.902 | 0.911 | .055 (.045, .066) | .064 |
| Quadratic | 60.67(19)*** | 0.951 | 0.946 | .043 (.031, .056) | .050 |
| Piecewise turning point | 59.31(19)*** | 0.953 | 0.948 | .042 (.031, .055) | .044 |
| Piecewise 2 intercepts | 39.87(14)*** | 0.970 | 0.954 | .040 (.025, .054) | .035 |
| Latent basis^3^ | - | - | - | - | - |
| National identification |  |  |  |  |  |
| Linear | 184.32(23)*** | 0.823 | 0.839 | .077 (.067, .087) | .125 |
| Quadratic | 169.02(19)*** | 0.836 | 0.818 | .081 (.070, .093) | .125 |
| Piecewise turning point | 165.98(19)*** | 0.839 | 0.822 | .081 (.070, .092) | .120 |
| Piecewise 2 intercepts | 99.10(14)*** | 0.907 | 0.860 | .071 (.059, .085) | .112 |
| Latent basis | 88.16(18)*** | 0.923 | 0.910 | .057 (.046, .069) | .064 |

*Notes.* Error messages: ^1^ Covariance matrix not positive definite. ^2^ Standard errors could not be computed. ^3^ No convergence, ^4^ Chi-square could not be computed.

*Table D7.* Model fit indices for growth functions of auxiliary variables among immigrant-origin Christian.

|  | X^2^(*df*) | CFI | TLI | RMSEA, 90% CI | SRMR |
| --- | --- | --- | --- | --- | --- |
| Anxiety |  |  |  |  |  |
| Linear^1^ | 4.55(1)* | 0.981 | 0.944 | .067 (.015, .134) | .025 |
| Latent basis | 0.42(1) | 1.000 | 1.000 | .000 (.000, .081) | .011 |
| Depression |  |  |  |  |  |
| Linear^1^ | 40.16(5)*** | 0.898 | 0.878 | .094 (.068, .122) | .074 |
| Quadratic | 0.81(1) | 1.000 | 1.000 | .000 (.000, .090) | .007 |
| Latent basis^4^ | - | - | - | - | - |
| Life satisfaction |  |  |  |  |  |
| Linear | 90.63(23)*** | 0.788 | 0.806 | .061 (.048, .074) | .098 |
| Quadratic | 78.16(19)*** | 0.814 | 0.795 | .063 (.048, .077) | .088 |
| Piecewise turning point | 31.50(14)** | 0.945 | 0.918 | .040 (.021, .058) | .036 |
| Piecewise 2 intercepts | 84.34(19)*** | 0.795 | 0.773 | .066 (.052, .080) | .095 |
| Latent basis^3^ | - | - | - | - | - |
| Health |  |  |  |  |  |
| Linear | 12.07(5)* | 0.973 | 0.967 | .042 (.011, .073) | .046 |
| Quadratic | 0.60(1) | 1.000 | 1.000 | .000 (.000, .084) | .006 |
| Latent basis | 13.30(3)** | 0.960 | 0.921 | .066 (.033, .103) | .050 |
| Gender role values |  |  |  |  |  |
| Linear | 34.25(5)*** | 0.924 | 0.909 | .086 (.060, .114) | .051 |
| Quadratic^1^ | 20.22(1)*** | 0.950 | 0.701 | .155 (.101, .218) | .040 |
| Latent basis | 4.06(3) | 0.997 | 0.995 | .021 (.000, .067) | .025 |
| Tolerance |  |  |  |  |  |
| Linear | 38.86(5)*** | 0.933 | 0.920 | .092 (.067, .120) | .057 |
| Quadratic^1^ | 2.21(1) | 0.998 | 0.986 | .039 (.000, .111) | .015 |
| Latent basis | 12.42(3)** | 0.981 | 0.963 | .063 (.029, .101) | .075 |
| Drinking |  |  |  |  |  |
| Linear | 117.06(16)*** | 0.853 | 0.863 | .092 (.077, .108) | .084 |
| Quadratic | 51.66(12)*** | 0.942 | 0.928 | .067 (.049, .086) | .050 |
| Piecewise turning point | 48.33(12)*** | 0.947 | 0.934 | .064 (.046, .083) | .054 |
| Piecewise 2 intercepts | 11.65(7) | 0.993 | 0.986 | .030 (.000, .059) | .028 |
| Latent basis^1^ | 60.20(12)*** | 0.930 | 0.913 | .073 (.056, .092) | .108 |
| Smoking |  |  |  |  |  |
| Linear | 116.61(16)*** | 0.878 | 0.886 | .092 (.007, .108) | .086 |
| Quadratic^1^ | 49.20(12)*** | 0.955 | 0.944 | .065 (.046, .084) | .039 |
| Piecewise turning point^1^ | 43.10(12)*** | 0.962 | 0.953 | .059 (.041, .079) | .037 |
| Piecewise 2 intercepts^1^ | 17.14(7)* | 0.988 | 0.974 | .044 (.018, .071) | .033 |
| Latent basis | 74.44(12)*** | 0.924 | 0.905 | .084 (.066, .102) | .067 |
| Drugs |  |  |  |  |  |
| Linear | 22.72(10)* | 0.858 | 0.858 | .042 (.019, .064) | .108 |
| Quadratic^1^ | 6.43(6) | 0.995 | 0.992 | .010 (.000, .050) | .056 |
| Piecewise turning point | 7.54(6) | 0.983 | 0.971 | .019 (.000, .054) | .057 |
| Latent basis | 8.78(7) | 0.980 | 0.972 | .019 (.000, .051) | .067 |
| German culture adoption |  |  |  |  |  |
| Linear | 24.13(10)** | 0.956 | 0.956 | .042 (.021, .064) | .078 |
| Quadratic | 15.56(6)* | 0.967 | 0.945 | .047 (.021, .075) | .056 |
| Piecewise turning point | 13.24(6)* | 0.977 | 0.962 | .039 (.008, .068) | .057 |
| Latent basis | 11.76(7) | 0.985 | 0.979 | .029 (.000, .058) | .035 |
| Heritage culture maintenance |  |  |  |  |  |
| Linear | 22.90(10)* | 0.956 | 0.956 | .040 (.018, .062) | .073 |
| Quadratic | 13.18(6)* | 0.975 | 0.959 | .039 (.008, .068) | .059 |
| Piecewise turning point | 9.47(6) | 0.988 | 0.980 | .027 (.000, .058) | .047 |
| Latent basis^3^ | - | - | - | - | - |
| German friends |  |  |  |  |  |
| Linear | 118.80(23)*** | 0.912 | 0.919 | .073 (.060, .086) | .130 |
| Quadratic | 49.66(19)*** | 0.972 | 0.969 | .045 (.030, .061) | .066 |
| Piecewise turning point | 59.07(19)*** | 0.963 | 0.959 | .052 (.037, .067) | .064 |
| Piecewise 2 intercepts | 32.06(14)** | 0.983 | 0.975 | .040 (.022, .059) | .039 |
| Latent basis^3^ | - | - | - | - | - |
| National identification |  |  |  |  |  |
| Linear | 146.52(23)*** | 0.892 | 0.901 | .082 (.070, .095) | .141 |
| Quadratic^1^ | 110.36(19)*** | 0.920 | 0.912 | .078 (.064, .092) | .134 |
| Piecewise turning point^1^ | 116.96(19)*** | 0.914 | 0.905 | .080 (.067, .095) | .136 |
| Piecewise 2 intercepts^1^ | 51.70(14)*** | 0.967 | 0.951 | .058 (.042, .076) | .115 |
| Latent basis | 90.89(18)*** | 0.936 | 0.926 | .071 (.057, .086) | .087 |

*Notes.* Error messages: ^1^ Covariance matrix not positive definite. ^2^ Standard errors could not be computed. ^3^ No convergence, ^4^ Chi-square could not be computed.

*Table D8.* Model fit indices for growth functions of auxiliary variables among non-immigrant Christians.

|  | X^2^(*df*) | CFI | TLI | RMSEA, 90% CI | SRMR |
| --- | --- | --- | --- | --- | --- |
| Anxiety |  |  |  |  |  |
| Linear | 24.81(1)*** | 0.950 | 0.850 | .107 (.073, .145) | .032 |
| Latent basis | 8.75(1)** | 0.984 | 0.951 | .061 (.029, .101) | .030 |
| Depression |  |  |  |  |  |
| Linear | 82.34(5)*** | 0.914 | 0.896 | .086 (.070, .103) | .052 |
| Quadratic^1^ | 10.71(1)** | 0.989 | 0.935 | .068 (.036, .108) | .017 |
| Latent basis^2^ | - | - | - | - | - |
| Life satisfaction |  |  |  |  |  |
| Linear | 192.97(23)*** | 0.784 | 0.803 | .060 (.052, .067) | .089 |
| Quadratic | 159.19(19)*** | 0.822 | 0.803 | .060 (.051, .068) | .086 |
| Piecewise turning point | 168.11(19)*** | 0.810 | 0.790 | .061 (.053, .070) | .083 |
| Piecewise 2 intercepts | 105.27(14)*** | 0.884 | 0.826 | .056 (.046, .066) | .077 |
| Latent basis^3^ | - | - | - | - | - |
| Health |  |  |  |  |  |
| Linear | 12.99(5)* | 0.991 | 0.989 | .028 (.009, .047) | .033 |
| Quadratic | 6.70(1)** | 0.993 | 0.961 | .052 (.021, .093) | .012 |
| Latent basis | 9.19(3)* | 0.993 | 0.986 | .031 (.009, .056) | .028 |
| Gender role values |  |  |  |  |  |
| Linear | 106.30(5)*** | 0.882 | 0.858 | .099 (.083, .115) | .060 |
| Quadratic^1^ | 78.77(1)*** | 0.909 | 0.456 | .193 (.158, .230) | .043 |
| Latent basis | 31.40(3)*** | 0.967 | 0.934 | .067 (.047, .090) | .040 |
| Tolerance |  |  |  |  |  |
| Linear | 202.73(5)*** | 0.830 | 0.796 | .138 (.122, .154) | .049 |
| Quadratic | 5.43(1)* | 0.996 | 0.977 | .046 (.015, .087) | .015 |
| Latent basis | 13.23(3)** | 0.991 | 0.982 | .040 (.020, .064) | .061 |
| Drinking |  |  |  |  |  |
| Linear | 567.11(16)*** | 0.705 | 0.723 | .132 (.123, .141) | .084 |
| Quadratic^1^ | 282.50(12)*** | 0.855 | 0.819 | .107 (.096, .118) | .049 |
| Piecewise turning point^1^ | 261.04(12)*** | 0.867 | 0.833 | .102 (.092, .113) | .053 |
| Piecewise 2 intercepts^1^ | 64.57(7)*** | 0.969 | 0.934 | .064 (.051, .079) | .025 |
| Latent basis^1^ | 231.13(12)*** | 0.883 | 0.853 | .096 (.085, .107) | .070 |
| Smoking |  |  |  |  |  |
| Linear | 227.47(16)*** | 0.896 | 0.902 | .082 (.072, .091) | .083 |
| Quadratic | 59.48(12)*** | 0.977 | 0.971 | .045 (.034, .056) | .022 |
| Piecewise turning point | 59.28(12)*** | 0.977 | 0.971 | .045 (.034, .056) | .023 |
| Piecewise 2 intercepts | 9.69(7) | 0.999 | 0.997 | .014 (.000, .033) | .009 |
| Latent basis | 97.91(12)*** | 0.958 | 0.947 | .060 (.049, .071) | .058 |
| Drugs |  |  |  |  |  |
| Linear | 68.30(10)*** | 0.825 | 0.825 | .054 (.043, .067) | .089 |
| Quadratic^1^ | 31.52(6)*** | 0.923 | 0.872 | .046 (.031, .063) | .061 |
| Piecewise turning point | 25.44(6)*** | 0.942 | 0.903 | .041 (.025, .057) | .048 |
| Latent basis | 34.58(7)*** | 0.917 | 0.882 | .045 (.031, .060) | .049 |

*Notes.* Error messages: ^1^ Covariance matrix not positive definite. ^2^ Standard errors could not be computed. ^3^ No convergence, ^4^ Chi-square could not be computed.
